# Design and synthesis of novel 3-(thiophen-2-yl)-1,5-dihydro-2*H*-pyrrol-2-one derivatives bearing a hydrazone moiety as potential fungicides

**DOI:** 10.1186/s13065-018-0452-z

**Published:** 2018-07-17

**Authors:** Xiaobin Wang, Zhengjiao Ren, Mengqi Wang, Min Chen, Aiming Lu, Weijie Si, Chunlong Yang

**Affiliations:** 1 Jiangsu Key Laboratory of Pesticide Science, College of Sciences, Nanjing Agricultural University, Nanjing, 210095 China; 2 Key Laboratory of Monitoring and Management of Crop Diseases and Pest Insects, Ministry of Agriculture, Nanjing Agricultural University, Nanjing, 210095 China

**Keywords:** Tetramic acid, Hydrazone, Thiophene, Synthesis, Antifungal activity

## Abstract

**Background:**

Tetramic acid, thiophene and hydrazone derivatives were found to exhibit favorable antifungal activity. Aiming to discover novel template molecules with potent antifungal activity, a series of novel 3-(thiophen-2-yl)-1,5-dihydro-2*H*-pyrrol-2-one derivatives containing a hydrazone group were designed, synthesized, and evaluated for their antifungal activity.

**Results:**

The structures of 3-(thiophen-2-yl)-1,5-dihydro-2*H*-pyrrol-2-one derivatives bearing a hydrazone group were confirmed by FT-IR, ^1^H NMR, ^13^C NMR, ^1^H-^1^H NOESY, EI-MS and elemental analysis. Antifungal assays indicated that some title compounds exhibited antifungal activity against *Fusarium graminearum* (*Fg*), *Rhizoctorzia solani* (*Rs*), *Botrytis cinerea* (*Bc*) and *Colletotrichum capsici* (*Cc*) in vitro. Strikingly, the EC_50_ value of **5e** against *Rs* was 1.26 µg/mL, which is better than that of drazoxolon (1.77 µg/mL). Meanwhile, title compounds **5b**, **5d**, **5e**–**5g**, **5n**–**5q** and **5t** exhibited remarkable anti-*Cc* activity, with corresponding EC_50_ values of 7.65, 9.97, 6.04, 6.66, 7.84, 7.59, 9.47, 5.52, 6.41 and 7.53 µg/mL, respectively, which are better than that of drazoxolon (19.46 µg/mL).

**Conclusions:**

A series of 3-(thiophen-2-yl)-1,5-dihydro-2*H*-pyrrol-2-one derivatives bearing a hydrazone group were designed, synthesized and evaluated for their antifungal activity against *Fg*, *Rs*, *Bc* and *Cc*. Bioassays indicated that some target compounds exhibited obvious antifungal activity against the above tested fungi. These results provide a significant basis for the further structural optimization of tetramic acid derivatives as potential fungicides.

**Electronic supplementary material:**

The online version of this article (10.1186/s13065-018-0452-z) contains supplementary material, which is available to authorized users.

## Background

An emergence of resistant fungi is a huge impetus to the development of agricultural fungicides with novel molecular structures and unique mechanisms [1]. In this regard, the structural optimization of natural heterocycles plays a important role in the searching for bioactive lead compounds [2, 3]. As attractive nitrogenous heterocycles, tetramic acid derivatives are widely researched for some reasons. First, tetramic acid derivatives exist in secondary metabolites from various terrestrial and marine organisms and have favorable compatibility with the environment [4]. Second, tetramic acid derivatives contain a unique pyrroline-2-one or pyrrolidine-2,4-dione substructure that is easy to synthesize to some extent [5]. Third, tetramic acid derivatives are reported to exhibit various agricultural bioactivities including fungicidal [6], herbicidal [7], insecticidal [8], antibacterial and antiviral [9] properties. Encouraged by the above findings, series of tetramic acid derivatives bearing amino [10], strobilurin [6], phenylhydrazine [11], oxime ether [12] and pyrrole [13] groups were synthesized and reported for their antifungal activity against plant fungi in our previous work. However, the potential application of tetramic acid derivatives as agricultural fungicides was greatly limited by their unsatisfactory curative rates [6, 10–13].

Thiophene is an important sulphureous compound that was widely studied for the development of novel fungicides due to their wide and satisfactory antifungal activity [14–17]. As important thiophene derivatives, thicyofen, ethaboxam, silthiopham and penthiopyrad were commercialized as agricultural fungicides in the past decades. Meanwhile, hydrazone is a widely researchful substructure that exists in commercialized agrochemicals including ferimzone, hydramethylnon, diflufenzopyr, pymetrozine, metaflumizone and benquinox [18, 19]. Recently, scholars found introducing a hydrazone group into salicylaldehyde [20], nalidixic acid [21], tetrahydro-*β*-carboline [22], 1,2,3-triazole [23], benzimidazole [24], diphenyl ether [25], pyrazole amide [26] quinoxaline [27] and carbonic acid ester [28] could effectively improve and broaden their antifungal activity. Obviously, further structural modifications of thiophene and hydrazone derivatives are significant for the development of novel fungicides.

**Fig. 1:**
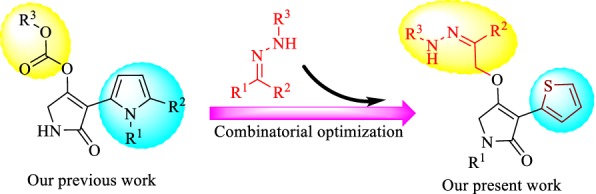
Design strategy of title compounds

**Scheme 1:**
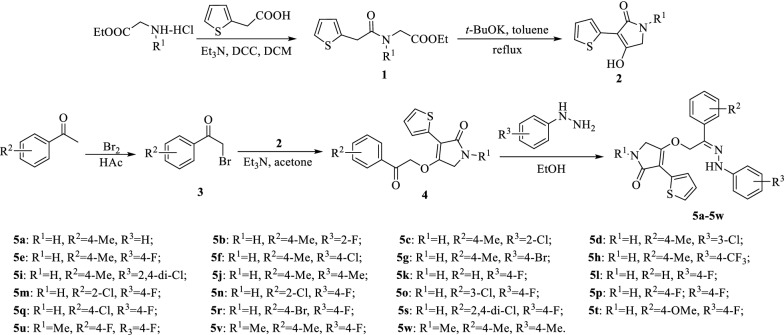
Synthesis route to title compounds

Aiming to extend our previous works on searching for pyrroline-2-one derivatives as agricultural fungicides [6, 10–13, 29], we theorized that introducing a hydrazone group into pyrroline-2-one structure might generate novel lead molecules with better antifungal activity (Fig. 1). Thus, in this study, a thiophene group was firstly neatly combined with pyrroline-2-one scaffold in one molecule by a Dieckmann cyclization. Subsequently, a hydrazone group was introduced into the 4-position of the obtained 3-(thiophen-2-yl)-1,5-dihydro-2*H*-pyrrol-2-one substructure to generate a series of novel tetramic acid derivatives (Scheme 1). In addition, the fungi *Fusarium graminearum* (*Fg*), *Rhizoctorzia solani* (*Rs*), *Botrytis cinerea* (*Bc*) and *Colletotrichum capsici* (*Cc*), which seriously restricted agricultural outputs of wheat, rice, strawberries and pepper, were selected as tested fungi to evaluate the antifungal activity of 3-(thiophen-2-yl)-1,5-dihydro-2*H*-pyrrol-2-one derivatives bearing a hydrazone group. To the best of our knowledge, it is the first report on the synthesis and antifungal activity of 3-(thiophen-2-yl)-1,5-dihydro-2*H*-pyrrol-2-one derivatives bearing a hydrazone group.

## Results and discussion

### Chemistry

The synthetic route to 3-(thiophen-2-yl)-1,5-dihydro-2*H*-pyrrol-2-one derivatives containing a hydrazone group is shown in Scheme 1. Using a substituted glycine ethyl ester hydrochloride as a starting material, the key intermediate **2** (substituted 4-hydroxy-3-(thiophen-2-yl)-1,5-dihydro-2*H*-pyrrol-2-one) was synthesized by two steps including amidation and cyclization reactions. The intermediate **2** was reacted with substituted 2-bromo-1-phenylethan-1-one **3** in acetone containing triethylamine to obtain the substituted 4-(2-oxo-2-phenylethoxy)-3-(thiophen-2-yl)-1,5-dihydro-2*H*-pyrrol-2-one **4**. Subsequently, the obtained intermediate **4** was reacted with substituted phenylhydrazine in acetonitrile to yield the title compound **5** with a good yield. The structures of title compounds were confirmed by FT-IR, ^1^H NMR, ^13^C NMR, EI-MS, and elemental analysis. In the IR spectra of title compounds, two obvious peaks at 3294–3447 and 3171–3263 cm^−1^ are attributed to the N–H stretching vibrations at pyrroline-2-one and phenylhydrazone fragments. The absorption peak of the carbonyl group at 2-position of pyrroline-2-one appears at 1682–1667 cm^−1^. In ^1^H NMR spectra, two singlets at δ 9.12–10.35 and 7.83–8.00 ppm are assigned to the NH protons at phenylhydrazone and pyrroline-2-one fragments. Two singlets at δ 4.26–4.49 and 5.36–5.58 ppm mean that the structure of title compounds has two –CH_2_– fragments. A typical carbon resonance at δ 169.51–172.01 ppm in the ^13^C NMR spectra confirms the presence of a carbonyl group at 2-position of pyrroline-2-one. Meanwhile, singlets at 43.51–43.77 and 61.73–66.02 ppm confirm the existence of two –CH_2_– fragments in the molecular structure of title compounds. In the EI-MS spectra of title compounds, the value of [M]^+^ ion absorption signal is consistent with the calculated value of molecular weight.

### Configuration confirmation of title compounds

**Fig. 2:**
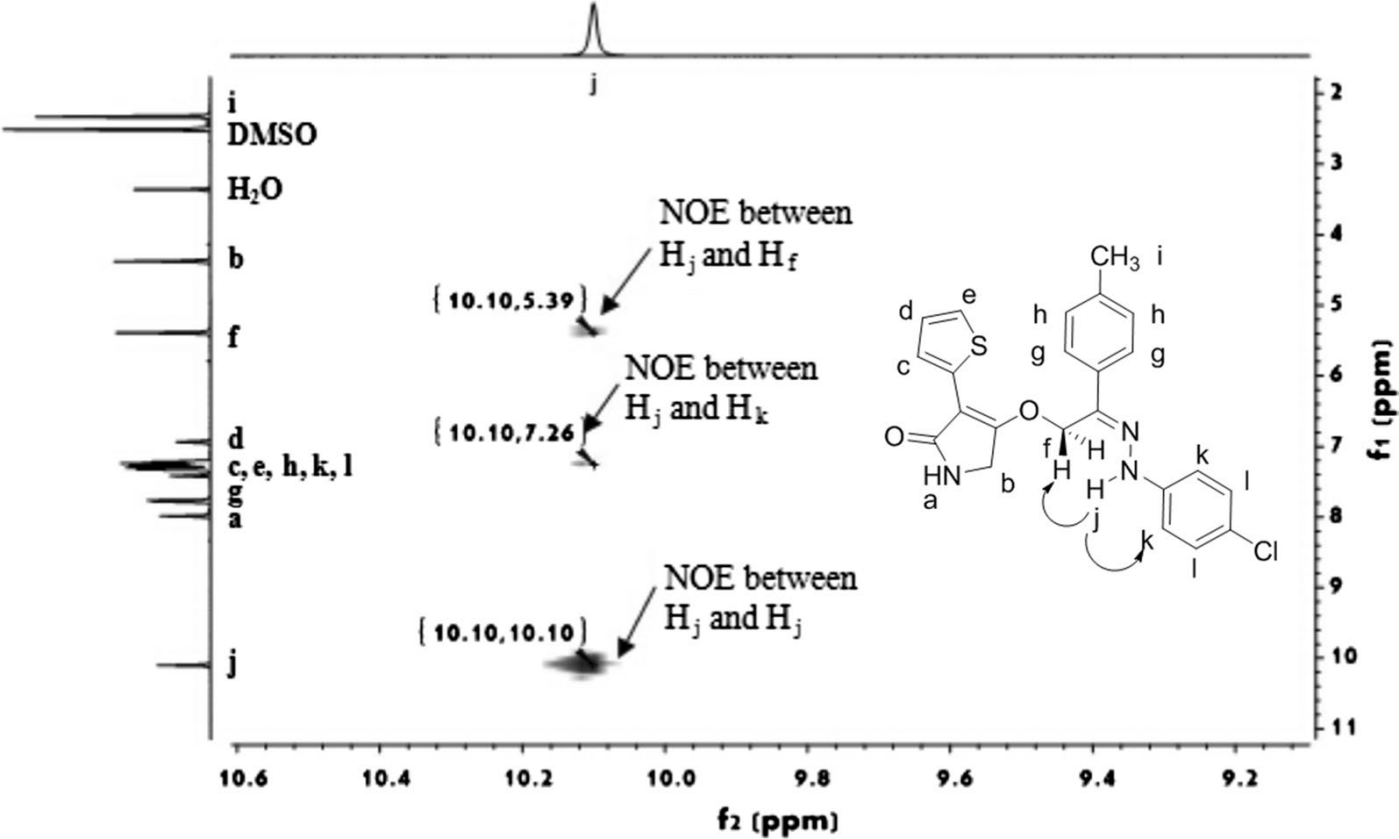
NOESY spectrum of the title compound **5f**

As shown in the ^1^H NMR and ^13^C NMR spectra of title compounds, these 3-(thiophen-2-yl)-1,5-dihydro-2*H*-pyrrol-2-one derivatives containing a hydrazone group does present itself via one single molecular structure. Aiming to further understand the structural characteristics of title compounds, the configuration of compound **5f** was studied as an example by a ^1^H-^1^H NOESY analysis [30]. As shown in Fig. 2, the chemical shifts of H_f_, H_j_ and H_k_ protons were 5.39, 10.10 and 7.26 ppm in the NOESY spectrum of compound **5f** (DMSO-*d*_6_), respectively. The obvious NOE phenomena between H_j_ and H_f_, and between H_j_ and H_k_ indicated that these protons close with each other, which typically revealed the double bond C=NNH of title compound **5f** possesses the *cis*-configuration.

### Antifungal activity screening of title compounds

Using a mycelial growth rate method [6, 10–13, 21–28], the antifungal effects of title compounds **5a**–**5w** against *Rs*, *Bc*, *Cc* and *Fg* were evaluated at 10 μg/mL and are shown in Table 1. A agricultural fungicide drazoxolon was used as a positive control of antifungal effects under same conditions. As shown in Table 1, the compounds **5n**, **5p** and **5u** exhibited fine activity against *Rs*, with inhibitory rates of 91.5, 100.0 and 84.7%, respectively, which are better than that of drazoxolon (84.5%). The compounds **5g**, **5p** and **5t** obviously inhibited the mycelium growth of *Bc,* with inhibitory rates of 66.4, 61.1 and 51.3%, respectively. The inhibition rates of compounds **5b**, **5d**–**5g**, **5m**–**5r**, **5t** and **5u** against *Cc* ranged from 48.5 to 100.0%, which are better than that of drazoxolon (46.8%). Table 1 also shown that the anti-*Fg* effects of target compounds **5e**–**5g**, **5o**–**5r** and **5t** at 10 μg/mL were 98.6, 69.0, 67.4, 74.6, 100.0, 68.6, 67.6 and 92.7%, respectively, which are apparently better than that of drazoxolon (67.2%).

**Table 1:** Antifungal effects of title compounds **5a–5w** at 10 μg/mL

Compd.	R^1^	R^2^	R^3^	*Rs*	*Bc*	*Cc*	*Fg*
**5a**	H	4-CH_3_	H	10.5 ± 0.5	0.0 ± 8.2	16.4 ± 2.6	7.5 ± 0.5
**5b**	H	4-CH_3_	2-F	67.7 ± 0.5	32.3 ± 0.9	57.3 ± 2.7	28.4 ± 1.1
**5c**	H	4-CH_3_	2-Cl	12.6 ± 1.7	25.3 ± 1.8	21.1 ± 1.3	12.2 ± 1.7
**5d**	H	4-CH_3_	3-Cl	70.6 ± 2.1	49.0 ± 1.6	61.3 ± 1.8	40.6 ± 2.3
**5e**	H	4-CH_3_	4-F	83.9 ± 1.2	49.5 ± 2.2	90.7 ± 1.7	98.6 ± 0.5
**5f**	H	4-CH_3_	4-Cl	76.7 ± 1.5	34.4 ± 0.5	74.7 ± 1.2	69.0 ± 0.6
**5g**	H	4-CH_3_	4-Br	66.7 ± 0.6	66.4 ± 2.1	74.7 ± 1.9	67.4 ± 1.7
**5h**	H	4-CH_3_	4-CF_3_	44.1 ± 3.0	38.4 ± 2.4	43.2 ± 2.1	42.3 ± 1.2
**5i**	H	4-CH_3_	2,4-di-Cl	47.5 ± 2.4	40.2 ± 3.1	18.9 ± 2.7	10.8 ± 3.5
**5j**	H	4-CH_3_	4-CH_3_	40.7 ± 1.8	27.8 ± 0.7	38.9 ± 1.8	40.5 ± 1.9
**5k**	H	4-CH_3_	4-OCH_3_	20.0 ± 3.5	7.3 ± 1.4	25.1 ± 1.0	9.4 ± 2.8
**5l**	H	H	4-F	52.8 ± 1.1	30.5 ± 3.2	40.7 ± 2.1	38.1 ± 2.1
**5m**	H	2-Cl	4-F	66.3 ± 1.4	37.5 ± 2.2	50.2 ± 2.8	47.3 ± 1.3
**5n**	H	2-Br	4-F	91.5 ± 2.0	47.2 ± 1.0	93.0 ± 1.3	53.1 ± 1.1
**5o**	H	3-Cl	4-F	70.2 ± 1.8	36.1 ± 0.9	73.8 ± 1.2	74.6 ± 3.2
**5p**	H	4-F	4-F	100.0 ± 0.3	61.1 ± 3.5	100.0 ± 0.2	100.0 ± 0.3
**5q**	H	4-Cl	4-F	76.4 ± 0.5	37.5 ± 1.4	83.8 ± 1.6	68.6 ± 2.3
**5r**	H	4-Br	4-F	55.0 ± 1.8	34.7 ± 2.2	48.5 ± 1.6	67.6 ± 3.0
**5s**	H	2,4-di-Cl	4-F	70.6 ± 3.3	45.8 ± 1.6	43.8 ± 3.0	47.8 ± 1.6
**5t**	H	4-OCH_3_	4-F	67.5 ± 1.2	51.3 ± 2.9	86.0 ± 3.6	92.7 ± 2.5
**5u**	4-CH_3_	4-F	4-F	84.7 ± 1.1	48.5 ± 2.1	53.8 ± 3.3	47.9 ± 1.4
**5v**	4-CH_3_	4-CH_3_	4-F	64.7 ± 2.1	36.3 ± 1.8	36.1 ± 1.1	39.6 ± 3.1
**5w**	4-CH_3_	4-CH_3_	4-CH_3_	37.1 ± 0.1	25.8 ± 0.4	31.2 ± 1.1	35.2 ± 2.1
**Drazoxolon** ^a^	/	/	/	84.5 ± 1.8	91.2 ± 2.2	46.8 ± 1.9	67.2 ± 0.9

Average of three replicates

^a^ A commercial agricultural fungicide drazoxolon was used for comparison of antifungal activity

Encouraged by the above preliminary bioassays, the EC_50_ values of some compounds that exhibited fine antifungal activity against *Rs*, *Cc* and *Fg* at 10 μg/mL were determined and are summarized in Table 2. Table 2 shown that the EC_50_ values of the selected compounds ranged from 1.26 to 9.89 µg/mL against *Rs*, from 5.52 to 9.97 µg/mL against *Cc* and from 6.02 to 8.85 µg/mL against *Fg*. Strikingly, the EC_50_ value of the title compound **5e** against *Rs* was 1.26 µg/mL, which is better than that of drazoxolon (1.77 µg/mL). Meanwhile, the title compounds **5b**, **5d**, **5e**–**5g**, **5n**–**5q** and **5t** had remarkable EC_50_ values of 7.65, 9.97, 6.04, 6.66, 7.84, 7.59, 9.47, 5.52, 6.41 and 7.53 µg/mL against *Cc*, respectively, which are better than that of drazoxolon (19.46 µg/mL). The above results also indicates that 3-(thiophen-2-yl)-1,5-dihydro-2*H*-pyrrol-2-one derivatives containing a hydrazone group can serve as potential structural templates in the search for novel and highly efficient fungicides.

**Table 2:** EC_50_ values of some title compounds against *Rs*, *Cc* and *Fg*

Compd.	Tested fungus	Regression equation	R	EC_50_ (µg/mL)
**5b**	*Rs*	y = 0.76x + 4.73	0.99	2.28 ± 3.00
	*Cc*	y = 0.81x + 4.28	0.95	7.65 ± 5.31
**5d**	*Rs*	y = 1.42x + 3.57	0.98	5.23 ± 3.74
	*Cc*	y = 1.60x + 2.95	0.98	9.97 ± 8.90
**5e**	*Rs*	y = 0.87x + 4.91	0.99	1.26 ± 1.12
	*Cc*	y = 1.42x + 3.89	0.99	6.04 ± 5.35
	*Fg*	y = 2.32x + 3.17	0.97	6.13 ± 4.49
**5f**	*Rs*	y = 0.50x + 4.74	0.99	3.32 ± 2.74
	*Cc*	y = 1.25x + 3.97	0.99	6.66 ± 5.33
	*Fg*	y = 1.74x + 3.54	0.99	6.90 ± 4.96
**5g**	*Rs*	y = 0.38x + 4.77	0.96	4.13 ± 2.83
	*Cc*	y = 1.32x + 3.82	0.99	7.84 ± 7.03
	*Fg*	y = 1.25x + 3.87	0.96	8.03 ± 5.01
**5n**	*Rs*	y = 1.26x + 4.31	0.99	3.56 ± 3.16
	*Cc*	y = 1.35x + 3.81	0.97	7.59 ± 5.12
**5o**	*Rs*	y = 1.42x + 3.79	0.98	7.15 ± 5.62
	*Cc*	y = 1.47x + 3.56	0.99	9.47 ± 8.02
	*Fg*	y = 1.97x + 3.10	0.99	7.22 ± 6.01
**5p**	*Rs*	y = 2.41x + 3.49	0.99	2.22 ± 1.68
	*Cc*	y = 4.22x + 1.87	0.99	5.52 ± 5.49
	*Fg*	y = 3.56x + 2.04	0.98	6.77 ± 5.14
**5q**	*Rs*	y = 1.76x + 3.73	0.99	5.29 ± 4.54
	*Cc*	y = 1.68x + 3.29	0.99	6.41 ± 4.96
	*Fg*	y = 3.79x + 1.30	0.99	7.63 ± 5.81
**5r**	*Rs*	y = 1.13x + 3.70	0.98	9.89 ± 7.18
	*Fg*	y = 1.33x + 3.47	0.99	8.85 ± 8.26
**5t**	*Rs*	y = 1.27x + 3.81	0.99	8.62 ± 7.06
	*Cc*	y = 1.39x + 3.24	0.98	7.53 ± 6.89
	*Fg*	y = 1.37x + 3.85	0.97	6.02 ± 5.26
**Drazoxolon** ^a^	*Rs*	y = 2.54x + 4.37	0.99	1.77 ± 1.62
	*Cc*	y = 0.82x + 3.94	0.99	19.46 ± 3.93
	*Fg*	y = 2.04x + 3.88	0.99	3.53 ± 2.72

Average of three replicates

^a^ A commercial agricultural fungicide drazoxolon was used for comparison of antifungal activity

### Structure–activity relationships

As indicated in Tables 1 and 2, the antifungal effects of title compounds were greatly affected by structural variations. Some structure–activity relationships (SAR) analyses were discussed as below. First, Tables 1 and 2 show that most of title compounds exhibited better antifungal activity against *Rs* than that against *Bc*, *Cc* and *Fg*. For example, Table 1 presents that the anti-*Rs* effects of title compounds **5b**, **5d**, **5f**, **5h**, **5i**, **5j**, **5l**, **5m**, **5p**, **5s**, **5u**, **5v** and **5w** are better than the corresponding effects against *Bc*, *Cc* and *Fg* at 10 μg/mL. Table 2 also exhibits that title compounds **5b**, **5d**, **5e**, **5f**, **5g**, **5n**, **5o**, **5p** and **5q** have better EC_50_ values against *Rs* than that against *Cc* and *Fg*. Second, introducing methyl into the R^1^ position is disadvantageous for the antifungal activity of title compounds against the tested four fungi. For instance, Table 1 shows that the inhibition rates of compounds **5e**, **5j** and **5p** (R^1^ = H) are obviously better than that of compounds **5v**, **5w** and **5u** (R^1^ = Me) against the tested four fungi at 10 μg/mL. Third, when the R^2^ was substituted by 4-Me, 4-F, 2-Br and 4-OMe groups, the corresponding title compounds **5e**, **5n**, **5p** and **5t** exhibited overall better antifungal activity than that of compounds **5l**, **5m**, **5o** and **5q**–**5s** against *Rs*, *Bc* and *Fg* at 10 μg/mL. Finally, a presence of 4-F, 4-Cl and 4-Br groups at the R^3^ position can effectively enhance the antifungal activity of title compounds against *Rs*, *Bc* and *Fg*. For example, the inhibition effects of compounds **5e**, **5f** and **5g** were overall better than that of compounds **5a**–**5d** and **5h**–**5k** against *Rs*, *Bc* and *Fg* at 10 μg/mL.

## Methods and materials

### General

Reagents and solvents used without further purification are analytically or chemically pure. Melting points (m.p.) were determined on an uncorrected WRS-1B digital melting point apparatus (Shanghai Precision and Scientific Instrument Corporation, China). The FT-IR spectra were recorded on a Thermo Nicolet 380 FT-IR spectrometer (Thermo Nicolet Corporation, America). ^1^H NMR, ^13^C NMR, and ^1^H-^1^H NOESY spectra were collected on a Bruker AV 400 MHz spectrometer (Bruker Corporation, Germany) at room temperature with DMSO-*d*_6_ as a solvent. Mass spectra were recorded on a TRACE 2000 spectrometer (Finnigan Corporation, America). Elemental analyses were determined on an Elementar Vario EL cube analyzer (Elementar Corporation, German). Reactions were monitored by thin layer chromatography (TLC) on silica gel GF_245_ (400 mesh). The tested strains *Fg*, *Rs*, *Bc* and *Cc* were provided by the Laboratory of Plant Disease Control at Nanjing Agricultural University.

### General procedures for intermediates **2** and **3**

Using glycine ethyl ester hydrochloride or alanine ethyl eater hydrochloride as a starting material, the intermediate **2a** (4-hydroxy-3-(thiophen-2-yl)-1,5-dihydro-2*H*-pyrrol-2-one) or **2b** (4-hydroxy-1-methyl-3-(thiophen-2-yl)-1,5-dihydro-2*H*-pyrrol-2-one) was successfully prepared according a previously procedure [31]. The substituted 2-bromo-1-phenylethan-1-ones **3a**–**3j** were synthesized according to a reported method [32].

### General procedures for intermediates **4**

A mixture of a intermediate **2** (10 mmol), a intermediate **3** (11 mmol) and triethylamine (11 mmol) in acetone (50 mL) was stirred at room temperature for 4 h. After that, the white solid appeared in the reaction solution was filtered, washed with water and diethyl ether to obtain a intermediate **4**.

#### 4-(2-oxo-2-(4-methylphenyl)ethoxy)-3-(thiophen-2-yl)-1,5-dihydro-2*H*-pyrrol-2-one (**4a**)

Yellow solid, m.p. 179–181 °C, yield 68%; ^1^H NMR (400 MHz, DMSO-*d*_*6*_) δ 8.02 (s, 1H, Pyrroline-1-H), 7.77 (d, *J* = 7.9 Hz, 2H, Ar(4-CH_3_)-2,6-2H), 7.63 (d, *J* = 3.0 Hz, 1H, Thiophene-3-H), 7.45 (d, *J* = 5.0 Hz, 1H, Thiophene-5-H), 7.25 (d, *J* = 7.9 Hz, 2H, Ar(4-CH_3_)-3.5-2H), 6.93 (t, *J* = 4.2 Hz, 1H, Thiophene-4-H), 5.38 (s, 2H, CH_2_), 4.38 (s, 2H, Pyrroline-5-2H), 2.32 (s, 3H, CH_3_).

#### 4-(2-oxo-2-phenylethoxy)-3-(thiophen-2-yl)-1,5-dihydro-2*H*-pyrrol-2-one (**4b**)

Yellow solid, m.p. 172–174 °C, yield 57%; ^1^H NMR (400 MHz, DMSO-*d*_*6*_) δ 7.97 (s, 1H, Pyrroline-1-H), 7.86 (d, *J* = 7.8 Hz, 2H, Ph-2,6-2H), 7.42 (d, *J* = 3.0 Hz, 1H, Thiophene-3-H), 7.38 (d, *J* = 5.0 Hz, 1H, Thiophene-5-H), 7.32 (t, *J* = 6.7 Hz, 2H, Ph-3,5-2H), 7.28–7.21 (m, 1H, Ph-4-H), 6.99–6.94 (m, 1H, Thiophene-4-H), 5.39 (s, 2H, CH_2_), 4.38 (s, 2H, Pyrroline-5-2H).

#### 4-(2-oxo-2-(2-chlorophenyl)ethoxy)-3-(thiophen-2-yl)-1,5-dihydro-2*H*-pyrrol-2-one (**4c**)

Yellow solid, m.p. 162–164 °C, yield 57%; ^1^H NMR (400 MHz, DMSO-*d*_*6*_) δ 8.05 (s, 1H, Pyrroline-1-H), 7.62 (dd, *J* = 5.7, 3.5 Hz, 1H, Ar(2-Cl)-3-H), 7.56 (dt, *J* = 7.3, 3.7 Hz, 1H, Ar(2-Cl)-4-H), 7.47 (dd, *J* = 5.7, 3.5 Hz, 2H, Thiophene-3,5-2H), 7.28 (d, *J* = 4.9 Hz, 1H, Ar(2-Cl)-6-H), 7.16 (d, *J* = 5,4 Hz, 1H, Thiophene-4-H), 6.87–6.80 (m, 1H, Ar(2-Cl)-5-H), 5.38 (s, 2H, CH_2_), 4.27 (s, 2H, Pyrroline-5-2H).

#### 4-(2-oxo-2-(2-bromophenyl)ethoxy)-3-(thiophen-2-yl)-1,5-dihydro-2*H*-pyrrol-2-one (**4d**)

Yellow solid, m.p. 152–154 °C, yield 34%; ^1^H NMR (400 MHz, DMSO-*d*_*6*_) δ 7.93 (s, 1H, Pyrroline-1-H), 7.68 (d, *J* = 7.8 Hz, 1H, Ar(2-Br)-6-H), 7.55 (d, *J* = 7.1 Hz, 1H, Ar(2-Br)-4-H), 7.46 (t, *J* = 7.4 Hz, 1H, Thiophene-3-H), 7.34 (t, *J* = 7.6 Hz, 1H, Thiophene-5-H), 7.28 (d, *J* = 4.8 Hz, 1H, Thiophene-4-H), 7.11 (d, *J* = 8.5 Hz, 2H, Ar(2-Br)-3,5-2H), 5.37 (s, 2H, CH_2_), 4.26 (s, 2H, Pyrroline-5-2H).

#### 4-(2-oxo-2-(3-chlorophenyl)ethoxy)-3-(thiophen-2-yl)-1,5-dihydro-2*H*-pyrrol-2-one (**4e**)

Yellow solid, m.p. 168–170 °C, yield 43%; ^1^H NMR (400 MHz, DMSO-*d*_*6*_) δ 7.98 (s, 1H, Pyrroline-1-H), 7.93 (s, 1H, Ar(3-Cl)-2-H), 7.84 (d, *J* = 7.7 Hz, 1H, Ar(3-Cl)-6-H), 7.42 (t, *J* = 8.5 Hz, 2H, Thiophene-3,5-2H), 7.32 (d, *J* = 4.9 Hz, 1H, Ar(3-Cl)-4-H), 7.23 (d, *J* = 8.7 Hz, 2H, Ar(3-Cl)-5-H, Thiophene-4-H), 5.41 (s, 2H, CH_2_), 4.37 (s, 2H, Pyrroline-5-2H).

#### 4-(2-oxo-2-(4-fluorophenyl)ethoxy)-3-(thiophen-2-yl)-1,5-dihydro-2*H*-pyrrol-2-one (**4f**)

Yellow solid, m.p. 174–176 °C, yield 56%; ^1^H NMR (400 MHz, DMSO-*d*_*6*_) δ 7.97 (s, 1H, Pyrroline-1-H), 7.94–7.84 (m, 2H, Ar(4-F)-2,6-2H), 7.41 (d, *J* = 2.6 Hz, 1H, Thiophene-3-H), 7.32 (d, *J* = 4.8 Hz, 1H, Thiophene-5-H), 7.12 (t, *J* = 8.6 Hz, 2H, Ar(4-F)-3,5-2H), 6.99–6.85 (m, 1H, Thiophene-4-H), 5.40 (s, 2H, CH_2_), 4.37 (s, 2H, Pyrroline-5-2H).

#### 4-(2-oxo-2-(4-chlorophenyl)ethoxy)-3-(thiophen-2-yl)-1,5-dihydro-2*H*-pyrrol-2-one (**4g**)

Yellow solid, m.p. 145–147 °C, yield 91%; ^1^H NMR (400 MHz, DMSO-*d*_*6*_) δ 7.99 (s, 1H, Pyrroline-1-H), 7.89 (d, *J* = 8.6 Hz, 2H, Ar(4-Cl)-2,6-2H), 7.40 (d, *J* = 3.3 Hz, 1H, Thiophene-3-H), 7.32 (d, *J* = 4.9 Hz, 1H, Thiophene-3-H), 7.22 (d, *J* = 8.8 Hz, 2H, Ar(4-Cl)-3,5-2H), 6.93 (dd, *J* = 8.8, 4.8 Hz, 1H, Thiophene-4-H), 5.40 (s, 2H, CH_2_), 4.37 (s, 2H, Pyrroline-5-2H).

#### 4-(2-oxo-2-(4-bromophenyl)ethoxy)-3-(thiophen-2-yl)-1,5-dihydro-2*H*-pyrrol-2-one (**4h**)

Yellow solid, m.p. 156–158 °C, yield 71%; ^1^H NMR (400 MHz, DMSO-*d*_*6*_) δ 7.97 (s, 1H, Pyrroline-1-H), 7.94–7.86 (m, 2H, Ar(4-Br)-2,6-2H), 7.42 (d, *J* = 5.9 Hz, 1H, Thiophene-3-H), 7.32 (d, *J* = 4.9 Hz, 1H, Thiophene-5-H), 7.23 (d, *J* = 8.7 Hz, 2H, Ar(4-Br)-3,5-2H), 6.94–6.88 (m, 1H, Thiophene-4-H), 5.40 (s, 2H, CH_2_), 4.37 (s, 2H, Pyrroline-5-2H).

#### 4-(2-oxo-2-(2,4-dichlorophenyl)ethoxy)-3-(thiophen-2-yl)-1,5-dihydro-2*H*-pyrrol-2-one (**4i**)

Yellow solid, m.p. 152–154 °C, yield 44%; ^1^H NMR (400 MHz, DMSO-*d*_*6*_) δ 7.95 (s, 1H, Pyrroline-1-H), 7.68 (d, *J* = 1.5 Hz, 1H, Ar(2,4-2Cl)-3-H), 7.61 (d, *J* = 8.3 Hz, 1H, Thiophene-3-H), 7.51 (dd, *J* = 8.3, 1.5 Hz, 1H, Thiophene-5-H), 7.30 (d, *J* = 5.1 Hz, 1H, Ar(2,4-2Cl)-5-H), 7.11 (d, *J* = 8.7 Hz, 1H, Ar(2,4-2Cl)-6-H), 6.90–6.78 (m, 1H, Thiophene-4-H), 5.37 (s, 2H, CH_2_), 4.26 (s, 2H, Pyrroline-5-2H).

#### 4-(2-oxo-2-(4-methoxyphenyl)ethoxy)-3-(thiophen-2-yl)-1,5-dihydro-2*H*-pyrrol-2-one (**4j**)

Yellow solid, m.p. 156–158 °C, yield 57%; ^1^H NMR (400 MHz, DMSO-*d*_*6*_) δ 7.97 (s, 1H, Pyrroline-1-H), 7.84–7.76 (m, 2H, Ar(4-OCH_3_)-2,6-2H), 7.42 (d, *J* = 5.9 Hz, 1H, Thiophene-3-H), 7.32 (d, *J* = 4.9 Hz, 1H, Thiophene-5-H), 7.23 (d, *J* = 8.7 Hz, 2H, Ar(4-OCH_3_)-3,5-2H), 6.97–6.88 (m, 1H, Thiophene-4-H), 5.40 (s, 2H, CH_2_), 4.37 (s, 2H, Pyrroline-5-2H), 3.78 (s, 3H, CH_3_).

#### 4-(2-oxo-2-(4-fluorophenyl)ethoxy)-1-methyl-3-(thiophen-2-yl)-1,5-dihydro-2*H*-pyrrol-2-one (**4k**)

Yellow solid, m.p. 166–168 °C, yield 72%; ^1^H NMR (400 MHz, DMSO-*d*_*6*_) δ 7.90 (dd, *J* = 8.7, 5.6 Hz, 2H, Ar(4-F)-2,6-2H), 7.40 (d, *J* = 3.6 Hz, 1H, Thiophene-3-H), 7.32 (d, *J* = 5.1 Hz, 1H, Thiophene-5-H), 7.29 (d, *J* = 11.1 Hz, 2H, Ar(4-F)-3,5-2H), 6.94 (dd, *J* = 5.0, 3.8 Hz, 1H, Thiophene-4-H), 5.39 (s, 2H, CH_2_), 4.45 (s, 2H, Pyrroline-5-2H), 2.99 (s, 3H, CH_3_).

#### 4-(2-oxo-2-(4-methylphenyl)ethoxy)-1-methyl-3-(thiophen-2-yl)-1,5-dihydro-2*H*-pyrrol-2-one (**4l**)

Yellow solid, m.p. 143–145 °C, yield 59%; ^1^H NMR (400 MHz, DMSO-*d*_*6*_) δ 7.76 (d, *J* = 7.7 Hz, 2H, Ar(4-CH_3_)-2,6-2H), 7.41 (d, *J* = 1.8 Hz, 1H, Thiophene-3-H), 7.31 (d, *J* = 8.5 Hz, 3H, Thiophene-5-H, Ar(4-CH_3_)-3,5-2H), 7.00–6.95 (m, 1H, Thiophene-4-H), 5.37 (s, 2H, CH_2_), 4.45 (s, 2H, Pyrroline-5-2H), 2.99 (s, 3H, CH_3_), 2.32 (s, 3H, CH_3_).

### General procedures for intermediates **5**

A mixture of a intermediate **4** (1.50 mmol) and substituted phenylhydrazine (1.70 mmol) in acetonitrile (35 mL) was stirred under 35 °C. After the reaction was completed, the white solid appeared in the reaction solution was filtered and recrystallized with diethyl ether to obtain a title compound **5**.

#### (Z)-4-(2-(2-phenylhydrazono)-2-(4-methylphenyl)ethoxy)-3-(thiophen-2-yl)-1,5-dihydro-2*H*-pyrrol-2-one (**5a**)

Yellow solid, m.p. 153–155 °C, yield 65%; IR (KBr, cm^−1^): 3380, 3171, 3063, 1676; ^1^H NMR (400 MHz, DMSO-*d*_*6*_) δ 9.98 (s, 1H, Ar–NH=N), 7.97 (s, 1H, Pyrroline-1-H), 7.78 (s, 1H, Ar(4-CH_3_)-2-H), 7.76 (s, 1H, Ar(4-CH_3_)-6-H), 7.42 (d, *J* = 3.1 Hz, 1H, Thiophene-3-H), 7.33–7.29 (m, 1H, Thiophene-5-H), 7.25 (t, *J* = 8.4 Hz, 5H, Ph-2,3,5,6-4H, Thiophene-4-H), 7.21 (s, 1H, Ar(4-CH_3_)-3-H), 6.93 (dd, *J* = 4.9, 3.8 Hz, 1H, Ar(4-CH_3_)-5-H), 6.83 (t, *J* = 6.5 Hz, 1H, Ph-4-H), 5.40 (s, 2H, CH_2_), 4.38 (s, 2H, Pyrroline-5-2H), 2.32 (s, 3H, CH_3_); ^13^C NMR (100 MHz, DMSO-*d*_6_) δ 171.99, 167.05, 145.68, 137.59, 136.57, 134.91, 132.66, 129.59, 129.51, 126.77, 125.80, 124.57, 124.04, 120.25, 113.44, 103.76, 61.76, 43.65, 21.28; Anal. Calcd for C_23_H_21_N_3_O_2_S (403.14): C, 68.46; H, 5.25; N, 10.41. Found: C, 68.22; H, 5.27; N, 10.37; EI-MS *m/z* 403.14 [M]^+^.

#### (Z)-4-(2-(2-(2-fluorophenyl)hydrazono)-2-(4-methylphenyl)ethoxy)-3-(thiophen-2-yl)-1,5-dihydro-2*H*-pyrrol-2-one (**5b**)

White solid, m.p. 158–160 °C, yield 51%; IR (KBr, cm^−1^): 3376, 3177, 3069, 1678; ^1^H NMR (400 MHz, DMSO-*d*_*6*_) δ 9.54 (s, 1H, Ar–NH=N), 7.95 (s, 1H, Pyrroline-1-H), 7.79 (d, *J* = 8.2 Hz, 2H, Ar(4-CH_3_)-2,6-2H), 7.62 (td, *J* = 8.5, 1.4 Hz, 1H, Thiophene-3-H), 7.43–7.39 (m, 1H, Thiophene-5-H), 7.32 (dd, *J* = 5.1, 0.9 Hz, 1H, Ar(2-F)-4-H), 7.24 (d, *J* = 8.1 Hz, 2H, Ar(2-F)-3,6-2H), 7.21–7.13 (m, 2H, Ar(4-CH_3_)-3,5-2H), 6.93 (dd, *J* = 5.1, 3.7 Hz, 1H, Ar(2-F)-5-H), 6.91–6.84 (m, 1H, Thiophene-4-H), 5.51 (s, 2H, CH_2_), 4.35 (s, 2H, Pyrroline-5-2H), 2.33 (s, 3H, CH_3_); ^13^C NMR (100 MHz, DMSO-*d*_6_) δ 171.91, 166.71, 151.54, 149.15, 140.53, 138.23, 134.40, 133.83, 133.74, 132.52, 129.53, 126.74, 126.29, 125.52, 125.48, 124.61, 124.07, 120.75, 120.69, 115.87, 103.82, 62.52, 43.58, 21.30; Anal. Calcd for C_23_H_20_FN_3_O_2_S (421.13): C, 65.54; H, 4.78; N, 9.97. Found: C, 65.12; H, 4.81; N, 9.92; EI-MS *m/z* 421.13 [M]^+^.

#### (Z)-4-(2-(2-(2-chlorophenyl)hydrazono)-2-(4-methylphenyl)ethoxy)-3-(thiophen-2-yl)-1,5-dihydro-2*H*-pyrrol-2-one (**5c**)

White solid, m.p. 160–162 °C, yield 30%; IR (KBr, cm^−1^): 3376, 3176, 3070, 1679; ^1^H NMR (400 MHz, DMSO-*d*_*6*_) δ 9.12 (s, 1H, Ar–NH=N), 7.99 (s, 1H, Pyrroline-1-H), 7.82 (d, *J* = 8.2 Hz, 2H, Ar(4-CH_3_)-2,6-2H), 7.64 (d, *J* = 7.2 Hz, 1H, Thiophene-3-H), 7.48 (d, *J* = 2.9 Hz, 1H, Thiophene-5-H), 7.36 (d, *J* = 4.1 Hz, 1H, Ar(2-Cl)-3-H), 7.35–7.30 (m, 2H, Thiophene-4-H, Ar(2-Cl)-5-H), 7.26 (d, *J* = 8.1 Hz, 2H, Ar(4-CH_3_)-3,5-2H), 6.96 (dd, *J* = 5.0, 3.7 Hz, 1H, Ar(2-Cl)-6-H), 6.92–6.86 (m, 1H, Ar(2-Cl)-4-H), 5.58 (s, 2H, CH_2_), 4.35 (s, 2H, Pyrroline-5-2H), 2.34 (s, 3H, CH_3_); ^13^C NMR (100 MHz, DMSO-*d*_6_) δ 171.74, 165.99, 141.52, 141.27, 138.68, 133.97, 132.25, 129.80, 129.67, 128.69, 126.76, 126.33, 124.85, 124.45, 121.55, 118.12, 115.32, 104.37, 63.53, 43.56, 21.31; Anal. Calcd for C_23_H_20_ClN_3_O_2_S (437.1): C, 63.08; H, 4.60; N, 9.60. Found: C, 62.82; H, 4.62; N, 9.57; EI-MS *m/z* 437.1 [M]^+^.

#### (Z)-4-(2-(2-(3-chlorophenyl)hydrazono)-2-(4-methylphenyl)ethoxy)-3-(thiophen-2-yl)-1,5-dihydro-2*H*-pyrrol-2-one (**5d**)

White solid, m.p. 172–174 °C, yield 38%; IR (KBr, cm^−1^): 3376, 3192, 3069, 1676; ^1^H NMR (400 MHz, DMSO-*d*_*6*_) δ 10.12 (s, 1H, Ar–NH=N), 7.97 (s, 1H, Pyrroline-1-H), 7.77 (d, *J* = 8.2 Hz, 2H, Ar(4-CH_3_)-2,6-2H), 7.41 (d, *J* = 2.8 Hz, 1H, Ar(3-Cl)-3-H), 7.34–7.30 (m, 1H, Thiophene-3-H), 7.27 (t, *J* = 5.2 Hz, 2H, Thiophene-4,5-2H), 7.25 (s, 1H, Ar(3-Cl)-5-H), 7.23 (s, 1H, Ar(3-Cl)-4-H), 7.18 (d, *J* = 8.2 Hz, 1H, Ar(4-CH_3_)-3-H), 6.93 (dd, *J* = 5.0, 3.7 Hz, 1H, Ar(4-CH_3_)-5-H), 6.85 (dd, *J* = 7.8, 1.1 Hz, 1H, Ar(3-Cl)-6-H), 5.39 (s, 2H, CH_2_), 4.37 (s, 2H, Pyrroline-5-2H), 2.33 (s, 3H, CH_3_); ^13^C NMR (100 MHz, DMSO-*d*_6_) δ 171.94, 166.89, 147.15, 138.39, 138.06, 134.53, 134.24, 132.60, 131.27, 129.57, 126.76, 126.05, 124.63, 124.07, 119.64, 112.79, 112.07, 103.83, 61.84, 43.63, 21.30; Anal. Calcd for C_23_H_20_ClN_3_O_2_S (437.1): C, 63.08; H, 4.60; N, 9.60. Found: C, 62.81; H, 4.64; N, 9.66; EI-MS *m/z* 437.1 [M]^+^.

#### (Z)-4-(2-(2-(4-fluorophenyl)hydrazono)-2-(4-methylphenyl)ethoxy)-3-(thiophen-2-yl)-1,5-dihydro-2*H*-pyrrol-2-one (**5e**)

White solid, m.p. 149–151 °C, yield 63%; IR (KBr, cm^−1^): 3368, 3167, 3063, 1676; ^1^H NMR (400 MHz, DMSO-*d*_*6*_) δ 10.05 (s, 1H, Ar–NH=N), 7.99 (s, 1H, Pyrroline-1-H), 7.76 (d, *J* = 8.0 Hz, 2H, Ar(4-CH_3_)-2,6-2H), 7.41 (d, *J* = 3.3 Hz, 1H, Thiophene-3-H), 7.32 (d, *J* = 5.0 Hz, 1H, Thiophene-5-H), 7.25 (dd, *J* = 10.0, 6.3 Hz, 3H, Thiophene-4-H, Ar(4-F)-3,5-2H), 7.21 (s, 1H, Ar(4-CH_3_)-3-H), 7.12 (t, *J* = 8.7 Hz, 2H, Ar(4-F)-2,6-2H), 6.95–6.90 (m, 1H, Ar(4-CH_3_)-5-H), 5.40 (s, 2H, CH_2_), 4.39 (s, 2H, Pyrroline-5-2H), 2.32 (s, 3H, CH_3_); ^13^C NMR (100 MHz, DMSO-*d*_6_) δ 171.97, 167.03, 158.07, 155.74, 142.37, 137.59, 136.71, 134.83, 132.65, 129.49, 126.76, 125.81, 124.56, 124.02, 116.20, 115.98, 114.53, 114.46, 103.74, 61.86, 43.66, 21.27; Anal. Calcd for C_23_H_20_FN_3_O_2_S (421.1): C, 65.54; H, 4.78; N, 9.97. Found: C, 65.81; H, 4.82; N, 9.89; EI-MS *m/z* 421.1 [M]^+^.

#### (Z)-4-(2-(2-(4-chlorophenyl)hydrazono)-2-(4-methylphenyl)ethoxy)-3-(thiophen-2-yl)-1,5-dihydro-2*H*-pyrrol-2-one (**5f**)

White solid, m.p. 156–157 °C, yield 61%; IR (KBr, cm^−1^): 3366, 3173, 3071, 1677; ^1^H NMR (400 MHz, DMSO-*d*_*6*_) δ 10.09 (s, 1H, Ar–NH=N), 7.97 (s, 1H, Pyrroline-1-H), 7.77 (d, *J* = 7.9 Hz, 2H, Ar(4-CH_3_)-2,6-2H), 7.41 (d, *J* = 2.7 Hz, 1H, Thiophene-3-H), 7.31 (d, *J* = 8.8 Hz, 3H, Thiophene-5-H, Ar(4-Cl)-3,5-2H), 7.25 (d, *J* = 10.3 Hz, 3H, Thiophene-4-H, Ar(4-Cl)-2,6-2H), 7.21 (s, 1H, Ar(4-CH_3_)-3-H), 6.96–6.90 (m, 1H, Ar(4-CH_3_)-5-H), 5.39 (s, 2H, CH_2_), 4.37 (s, 2H, Pyrroline-5-2H), 2.32 (s, 3H, CH_3_); ^13^C NMR (100 MHz, DMSO-*d*_6_) δ 171.96, 166.93, 144.64, 137.86, 137.60, 134.66, 132.62, 129.52, 129.41, 126.77, 125.93, 124.60, 124.06, 123.57, 114.90, 103.82, 61.81, 43.64, 21.29; Anal. Calcd for C_23_H_20_ClN_3_O_2_S (437.1): C, 63.08; H, 4.60; N, 9.60. Found: C, 63.51; H, 4.64; N, 9.67; EI-MS *m/z* 437.1 [M]^+^.

#### (Z)-4-(2-(2-(4-bromophenyl)hydrazono)-2-(4-methylphenyl)ethoxy)-3-(thiophen-2-yl)-1,5-dihydro-2*H*-pyrrol-2-one (**5g**)

White solid, m.p. 160–162 °C, yield 72%; IR (KBr, cm^−1^): 3364, 3179, 3075, 1677; ^1^H NMR (400 MHz, DMSO-*d*_*6*_) δ 10.09 (s, 1H, Ar–NH=N), 7.97 (s, 1H, Pyrroline-1-H), 7.77 (d, *J* = 8.1 Hz, 2H, Ar(4-CH_3_)-2,6-2H), 7.42 (d, *J* = 8.7 Hz, 3H, Thiophene-3,5-2H, Ar(4-Br)-3-H), 7.31 (d, *J* = 5.0 Hz, 1H, Ar(4-Br)-5-H), 7.21 (t, *J* = 8.1 Hz, 4H, Thiophene-4-H, Ar(4-CH_3_)-3,5-2H, Ar(4-Br)-2-H), 6.96–6.90 (m, 1H, Ar(4-Br)-6-H), 5.38 (s, 2H, CH_2_), 4.37 (s, 2H, Pyrroline-5-2H), 2.32 (s, 3H, CH_3_); ^13^C NMR (100 MHz, DMSO-*d*_6_) δ 171.95, 166.92, 145.02, 137.89, 137.70, 134.65, 132.62, 132.25, 129.53, 126.77, 125.94, 124.61, 124.06, 115.40, 111.25, 103.82, 61.82, 43.63, 21.29; Anal. Calcd for C_23_H_20_BrN_3_O_2_S (481.0): C, 57.27; H, 4.18; N, 8.71. Found: C, 57.14; H, 4.21; N, 8.72; EI-MS *m/z* 481.0 [M]^+^.

#### (Z)-4-(2-(2-(2-(4-(trifluoromethyl)phenyl)hydrazono)-2-(4-methylphenyl)ethoxy)-3-(thiophen-2-yl)-1,5-dihydro-2*H*-pyrrol-2-one (**5h**)

White solid, m.p. 167–169 °C, yield 82%; IR (KBr, cm^−1^): 3363, 3172, 3074, 1681, 1590; ^1^H NMR (400 MHz, DMSO-*d*_*6*_) δ 10.35 (s, 1H, Ar–NH=N), 7.98 (s, 1H, Pyrroline-1-H), 7.80 (d, *J* = 7.7 Hz, 2H, Ar(4-CF_3_)-3,5-2H), 7.61 (d, *J* = 8.3 Hz, 2H, Ar(4-CH_3_)-2,6-2H), 7.40 (s, 2H, Thiophene-3,5-2H), 7.38 (s, 1H, Ar(4-CF_3_)-2-H), 7.31 (d, *J* = 4.9 Hz, 1H, Ar(4-CF_3_)-6-H), 7.24 (d, *J* = 7.7 Hz, 2H, Ar(4-CH_3_)-3,5-2H), 6.92 (d, *J* = 3.7 Hz, 1H, Thiophene-4-H), 5.42 (s, 2H, CH_2_), 4.38 (s, 2H, Pyrroline-5-2H), 2.34 (s, 3H, CH_3_); ^13^C NMR (100 MHz, DMSO-*d*_6_) δ 171.94, 166.83, 148.76, 139.34, 138.29, 134.42, 132.59, 129.56, 126.98, 126.94, 126.76, 126.20, 124.64, 124.07, 120.13, 119.82, 113.23, 103.87, 61.87, 43.63, 21.30; Anal. Calcd for C_24_H_20_F_3_N_3_O_2_S (471.1): C, 61.14; H, 4.28; N, 8.91. Found: C, 61.21; H, 4.31; N, 8.89; EI-MS *m/z* 471.1 [M]^+^.

#### (Z)-4-(2-(2-(2-(2,4-dichlorophenyl)hydrazono)-2-(4-methylphenyl)ethoxy)-3-(thiophen-2-yl)-1,5-dihydro-2*H*-pyrrol-2-one (**5i**)

White solid, m.p. 172–174 °C, yield 39%; IR (KBr, cm^−1^): 3363, 3167, 3075, 1679; ^1^H NMR (400 MHz, DMSO-*d*_*6*_) δ 9.20 (s, 1H, Ar–NH=N), 8.00 (s, 1H, Pyrroline-1-H), 7.82 (d, *J* = 7.9 Hz, 2H, Ar(4-CH_3_)-2,6-2H), 7.64 (d, *J* = 8.9 Hz, 1H, Thiophene-3-H), 7.53 (s, 1H, Thiophene-6-H), 7.46 (d, *J* = 3.0 Hz, 1H, Thiophene-4-H), 7.39 (d, *J* = 8.8 Hz, 1H, Ar(2,4-2Cl)-6-H), 7.35 (d, *J* = 5.0 Hz, 1H, Ar(2,4-2Cl)-3-H), 7.26 (d, *J* = 7.9 Hz, 2H, Ar(4-CH_3_)-3,5-2H), 6.98–6.93 (m, 1H, Ar(2,4-2Cl)-5-H), 5.58 (s, 2H, CH_2_), 4.35 (s, 2H, Pyrroline-5-2H), 2.34 (s, 3H, CH_3_); ^13^C NMR (100 MHz, DMSO-*d*_6_) δ 171.73, 165.98, 142.51, 140.59, 138.88, 133.78, 132.24, 129.66, 129.09, 128.71, 126.75, 126.46, 124.84, 124.43, 124.26, 118.71, 116.46, 104.34, 63.57, 43.55, 21.32; Anal. Calcd for C_23_H_19_Cl_2_N_3_O_2_S (471.1): C, 58.48; H, 4.05; N, 8.90. Found: C, 58.23; H, 4.21; N, 8.86; EI-MS *m/z* 471.1 [M]^+^.

#### (Z)-4-(2-(2-(2-(4-methylphenyl)hydrazono)-2-(4-methylphenyl)ethoxy)-3-(thiophen-2-yl)-1,5-dihydro-2*H*-pyrrol-2-one (**5j**)

White solid, m.p. 141–143 °C, yield 42%; IR (KBr, cm^−1^): 3376, 3172, 3069, 1667; ^1^H NMR (400 MHz, DMSO-*d*_*6*_) δ 9.90 (s, 1H, Ar–NH=N), 7.98 (s, 1H, Pyrroline-1-H), 7.75 (d, *J* = 7.9 Hz, 2H, Ar(4-CH_3_)-2,6-2H), 7.42 (d, *J* = 3.0 Hz, 1H, Thiophene-3-H), 7.31 (d, *J* = 5.0 Hz, 1H, Thiophene-5-H), 7.21 (d, *J* = 7.9 Hz, 2H, Ar(4-CH_3_)-3.5-2H), 7.11 (dd, *J* = 27.3, 8.1 Hz, 4H, Ar(4-CH_3_)-2,3,4,5-4H), 6.93 (t, *J* = 4.2 Hz, 1H, Thiophene-4-H), 5.38 (s, 2H, CH_2_), 4.38 (s, 2H, Pyrroline-5-2H), 2.32 (s, 3H, CH_3_), 2.23 (s, 3H, CH_3_); ^13^C NMR (100 MHz, DMSO-*d*_6_) δ 171.98, 167.09, 143.42, 137.39, 135.82, 135.01, 132.67, 130.02, 129.49, 128.85, 126.76, 125.66, 124.55, 124.02, 113.44, 103.72, 61.73, 43.64, 21.27, 20.75; Anal. Calcd for C_24_H_23_N_3_O_2_S (417.1): C, 58.48; H, 4.05; N, 8.90. Found: C, 58.23; H, 4.07; N, 8.86; EI-MS *m/z* 417.1 [M]^+^.

#### (Z)-4-(2-(2-(2-(4-methoxyphenyl)hydrazono)-2-(4-methylphenyl)ethoxy)-3-(thiophen-2-yl)-1,5-dihydro-2*H*-pyrrol-2-one (**5k**)

White solid, m.p. 140–142 °C, yield 38%; IR (KBr, cm^−1^): 3376, 3177, 3069, 1679; ^1^H NMR (400 MHz, DMSO-*d*_*6*_) δ 9.82 (s, 1H, Ar–NH=N), 7.96 (s, 1H, Pyrroline-1-H), 7.74 (d, *J* = 8.2 Hz, 2H, Ar(4-CH_3_)-2,6-2H), 7.42 (d, *J* = 3.4 Hz, 1H, Thiophene-3-H), 7.32 (d, *J* = 5.0 Hz, 1H, Thiophene-5-H), 7.19 (t, *J* = 9.0 Hz, 4H, Thiophene-4-H, Ar(4-OCH_3_)-2,6-2H, Ar(4-CH_3_)-3-H), 6.96–6.92 (m, 1H, Ar(4-CH_3_)-5-H), 6.89 (d, *J* = 9.0 Hz, 2H, Ar(4-OCH_3_)-3,5-2H), 5.37 (s, 2H, CH_2_), 4.37 (s, 2H, Pyrroline-5-2H), 3.70 (s, 3H, CH_3_), 2.31 (s, 3H, CH_3_); ^13^C NMR (100 MHz, DMSO-*d*_6_) δ 172.01, 167.12, 153.75, 139.63, 137.22, 135.28, 135.10, 132.69, 129.48, 126.76, 125.56, 124.54, 124.03, 115.02, 114.50, 103.73, 61.75, 55.70, 43.65, 21.25; Anal. Calcd for C_24_H_23_N_3_O_3_S (433.1): C, 66.49; H, 5.35; N, 9.69. Found: C, 66.26; H, 5.33; N, 9.73; EI-MS *m/z* 433.1 [M]^+^.

#### (Z)-4-(2-(2-(2-(4-fluorophenyl)hydrazono)-2-phenylethoxy)-3-(thiophen-2-yl)-1,5-dihydro-2*H*-pyrrol-2-one (**5l**)

White solid, m.p. 131–133 °C, yield 44%; IR (KBr, cm^−1^): 3343, 3231, 3060, 1677; ^1^H NMR (400 MHz, DMSO-*d*_*6*_) δ 10.05 (s, 1H, Ar–NH=N), 7.97 (s, 1H, Pyrroline-1-H), 7.86 (d, *J* = 7.8 Hz, 2H, Ph-2,6-2H), 7.40 (d, *J* = 7.5 Hz, 3H, Thiophene-3,4,5-3H), 7.32 (t, *J* = 6.7 Hz, 2H, Ph-3,5-2H), 7.28–7.21 (m, 2H, Ar(4-F)-2,6-2H), 7.12 (t, *J* = 8.7 Hz, 2H, Ar(4-F)-3,5-2H), 6.95–6.90 (m, 1H, Ph-4-H), 5.40 (s, 2H, CH_2_), 4.38 (s, 2H, Pyrroline-5-2H); ^13^C NMR (100 MHz, DMSO-*d*_6_) δ 171.97, 166.96, 158.19, 155.85, 142.23, 137.58, 136.62, 132.63, 128.89, 128.20, 126.76, 125.88, 124.59, 124.04, 116.26, 116.04, 114.64, 114.56, 103.81, 61.81, 43.64; Anal. Calcd for C_22_H_18_FN_3_O_2_S (407.1): C, 64.85; H, 4.45; N, 10.31. Found: C, 64.78; H, 4.48; N, 10.37; EI-MS *m/z* 407.1 [M]^+^.

#### (Z)-4-(2-(2-chlorophenyl)-2-(2-(4-fluorophenyl)hydrazono)ethoxy)-3-(thiophen-2-yl)-1,5-dihydro-2*H*-pyrrol-2-one (**5m**)

Yellow solid, m.p. 125–127 °C, yield 46%; IR (KBr, cm^−1^): 3312, 3223, 3084, 1682; ^1^H NMR (400 MHz, DMSO-*d*_*6*_) δ 9.85 (s, 1H, Ar–NH=N), 7.95 (s, 1H, Pyrroline-1-H), 7.58 (dd, *J* = 5.7, 3.5 Hz, 1H, Ar(2-Cl)-3-H), 7.50 (dt, *J* = 7.3, 3.7 Hz, 1H, Ar(2-Cl)-4-H), 7.42 (dd, *J* = 5.7, 3.5 Hz, 2H, Thiophene-3,5-2H), 7.28 (d, *J* = 4.9 Hz, 1H, Thiophene-4-H), 7.20–7.06 (m, 4H, Ar(4-F)-2,3,6-3H, Ar(2-Cl)-6-H), 7.04 (d, *J* = 3.1 Hz, 1H, Ar(4-F)-5-H), 6.87–6.80 (m, 1H, Ar(2-Cl)-5-H), 5.38 (s, 2H, CH_2_), 4.27 (s, 2H, Pyrroline-5-2H); ^13^C NMR (100 MHz, DMSO-*d*_6_) δ 171.85, 166.09, 158.18, 155.85, 142.37, 139.22, 136.89, 132.68, 132.34, 131.70, 130.14, 129.95, 127.66, 126.58, 124.56, 124.09, 116.15, 115.93, 114.64, 114.57, 103.88, 65.93, 43.54; Anal. Calcd for C_22_H_17_FClN_3_O_2_S (441.1): C, 59.80; H, 3.88; N, 9.51. Found: C, 59.78; H, 3.90; N, 9.57; EI-MS *m/z* 441.1 [M]^+^.

#### (Z)-4-(2-(2-bromophenyl)-2-(2-(4-fluorophenyl)hydrazono)ethoxy)-3-(thiophen-2-yl)-1,5-dihydro-2*H*-pyrrol-2-one (**5n**)

Yellow solid, m.p. 132–134 °C, yield 35%; IR (KBr, cm^−1^): 3315, 3219, 3087, 1681; ^1^H NMR (400 MHz, DMSO-*d*_*6*_) δ 9.94 (s, 1H, Ar–NH=N), 7.96 (s, 1H, Pyrroline-1-H), 7.68 (d, *J* = 7.9 Hz, 1H, Ar(2-Br)-3-H), 7.55 (d, *J* = 6.2 Hz, 1H, Ar(2-Br)-4-H), 7.49–7.31 (m, 4H, Thiophene-3,5-2H, Ar(4-F)-2,6-2H), 7.28 (d, *J* = 4.9 Hz, 1H, Thiophene-4-H), 7.11 (d, *J* = 8.8 Hz, 2H, Ar(4-F)-3,5-2H), 7.00 (d, *J* = 3.2 Hz, 1H, Ar(2-Br)-6-H), 6.86–6.79 (m, 1H, Ar(2-Br)-5-H), 5.37 (s, 2H, CH_2_), 4.26 (s, 2H, Pyrroline-5-2H); ^13^C NMR (100 MHz, DMSO-*d*_6_) δ 171.86, 166.06, 142.42, 140.36, 138.78, 133.08, 132.32, 131.85, 130.31, 128.11, 126.58, 124.55, 124.16, 122.75, 116.95, 116.87, 116.28, 116.12, 116.05, 115.90, 114.63, 114.56, 103.90, 66.02, 43.62; Anal. Calcd for C_22_H_17_FBrN_3_O_2_S (485.0): C, 54.33; H, 3.52; N, 8.64. Found: C, 54.53; H, 3.55; N, 8.57; EI-MS *m/z* 485.0 [M]^+^.

#### (Z)-4-(2-(3-chlorophenyl)-2-(2-(4-fluorophenyl)hydrazono)ethoxy)-3-(thiophen-2-yl)-1,5-dihydro-2*H*-pyrrol-2-one (**5o**)

Yellow solid, m.p. 125–126 °C, yield 36%; IR (KBr, cm^−1^): 3375, 3255, 3067, 1682; ^1^H NMR (400 MHz, DMSO-*d*_*6*_) δ 10.18 (s, 1H, Ar–NH=N), 7.97 (s, 1H, Pyrroline-1-H), 7.89 (s, 1H, Ar(3-Cl)-2-H), 7.83 (d, *J* = 7.7 Hz, 1H, Ar(3-Cl)-6-H), 7.43 (t, *J* = 6.9 Hz, 2H, Thiophene-3,5-2H), 7.37 (d, *J* = 7.7 Hz, 1H, Thiophene-4-H), 7.32 (d, *J* = 5.0 Hz, 1H, Ar(3-Cl)-4-H), 7.29–7.22 (m, 2H, Ar(4-F)-2,6-2H), 7.14 (t, *J* = 8.6 Hz, 2H, Ar(4-F)-3,5-2H), 6.97–6.91 (m, 1H, Ar(3-Cl)-5-H), 5.40 (s, 2H, CH_2_), 4.38 (s, 2H, Pyrroline-5-2H); ^13^C NMR (100 MHz, DMSO-*d*_6_) δ 171.94, 166.89, 158.39, 156.05, 141.92, 139.73, 135.19, 133.89, 132.62, 130.72, 127.84, 126.77, 125.40, 124.62, 124.50, 123.99, 116.34, 116.11, 114.88, 114.80, 103.84, 61.62, 43.62; Anal. Calcd for C_22_H_17_ClBrN_3_O_2_S (441.1): C, 59.80; H, 3.88; N, 9.51. Found: C, 59.58; H, 3.85; N, 9.57; EI-MS *m/z* 441.1 [M]^+^.

#### (Z)-4-(2-(4-fluorophenyl)-2-(2-(4-fluorophenyl)hydrazono)ethoxy)-3-(thiophen-2-yl)-1,5-dihydro-2*H*-pyrrol-2-one (**5p**)

Yellow solid, m.p. 133–135 °C, yield 67%; IR (KBr, cm^−1^): 3355, 3229, 3087, 1678; ^1^H NMR (400 MHz, DMSO-*d*_*6*_) δ 10.07 (s, 1H, Ar–NH=N), 7.98 (s, 1H, Pyrroline-1-H), 7.94–7.85 (m, 2H, Ar(4-F)-2,6-2H), 7.40 (s, 1H, Thiophene-3-H), 7.32 (d, *J* = 4.7 Hz, 1H, Thiophene-5-H), 7.25 (d, *J* = 8.4 Hz, 4H, Ar(4-F)-2,6-2H, Ar(4-F)-3,5-2H), 7.12 (t, *J* = 8.5 Hz, 2H, Ar(4-F)-3,5-2H), 6.94 (s, 1H, Thiophene-4-H), 5.40 (s, 2H, CH_2_), 4.38 (s, 2H, Pyrroline-5-2H); ^13^C NMR (100 MHz, DMSO-*d*_6_) δ 171.95, 167.00, 163.52, 161.09, 159.09, 156.73, 142.56, 135.82, 135.76, 134.16, 134.13, 132.68, 128.04, 127.96, 126.72, 124.52, 123.95, 117.04, 116.96, 116.91, 116.11, 115.89, 103.72, 62.11, 43.77; Anal. Calcd for C_22_H_17_F_2_N_3_O_2_S (425.1): C, 62.11; H, 4.03; N, 9.88. Found: C, 62.49; H, 4.05; N, 9.86; EI-MS *m/z* 425.1 [M]^+^.

#### (Z)-4-(2-(4-chlorophenyl)-2-(2-(4-fluorophenyl)hydrazono)ethoxy)-3-(thiophen-2-yl)-1,5-dihydro-2*H*-pyrrol-2-one (**5q**)

Yellow solid, m.p. 131–133 °C, yield 83%; IR (KBr, cm^−1^): 3447, 3239, 3123, 1675; ^1^H NMR (400 MHz, DMSO-*d*_*6*_) δ 10.19 (s, 1H, Ar–NH=N), 7.99 (s, 1H, Pyrroline-1-H), 7.89 (d, *J* = 8.6 Hz, 2H, Ar(4-Cl)-2,6-2H), 7.46 (d, *J* = 8.6 Hz, 2H, Ar(4-F)-2,6-2H), 7.41 (d, *J* = 3.2 Hz, 1H, Thiophene-3-H), 7.32 (d, *J* = 4.7 Hz, 1H, Thiophene-5-H), 7.27 (dd, *J* = 9.0, 4.8 Hz, 2H, Ar(4-F)-3,5-2H), 7.13 (t, *J* = 8.8 Hz, 2H, Ar(4-Cl)-3,5-2H), 6.94 (dd, *J* = 4.9, 3.8 Hz, 1H, Thiophene-4-H), 5.41 (s, 2H, CH_2_), 4.39 (s, 2H, Pyrroline-5-2H); ^13^C NMR (100 MHz, DMSO-*d*_6_) δ 171.94, 166.89, 158.30, 155.96, 142.05, 136.47, 135.44, 132.69, 132.63, 128.87, 127.56, 126.76, 124.61, 124.02, 116.27, 116.05, 114.76, 114.68, 103.83, 61.60, 43.65; Anal. Calcd for C_22_H_17_FClN_3_O_2_S (441.1): C, 59.80; H, 3.88; N, 9.51. Found: C, 60.19; H, 3.90; N, 9.46; EI-MS *m/z* 441.1 [M]^+^.

#### (Z)-4-(2-(4-bromophenyl)-2-(2-(4-fluorophenyl)hydrazono)ethoxy)-3-(thiophen-2-yl)-1,5-dihydro-2*H*-pyrrol-2-one (**5r**)

White solid, m.p. 136–138 °C, yield 88%; IR (KBr, cm^−1^): 3294, 3223, 3079, 1679; ^1^H NMR (400 MHz, DMSO-*d*_*6*_) δ 10.16 (s, 1H, Ar–NH=N), 7.98 (s, 1H, Pyrroline-1-H), 7.91 (dd, *J* = 8.6, 5.6 Hz, 2H, Ar(4-Br)-2,6-2H), 7.43 (d, *J* = 8.7 Hz, 2H, Ar(4-F)-2,6-2H), 7.40 (d, *J* = 3.2 Hz, 1H, Thiophene-3-H), 7.32 (d, *J* = 4.9 Hz, 1H, Thiophene-5-H), 7.28–7.18 (m, 4H, Ar(4-F)-3,5-2H, Ar(4-Br)-3,5-2H), 6.96–6.90 (m, 1H, Thiophene-4-H), 5.40 (s, 2H, CH_2_), 4.37 (s, 2H, Pyrroline-5-2H); ^13^C NMR (100 MHz, DMSO-*d*_6_) δ 171.95, 166.88, 163.58, 161.14, 158.19, 155.85, 142.22, 142.21, 135.94, 134.07, 132.62, 128.02, 127.94, 126.76, 124.61, 124.03, 116.25, 116.03, 115.85, 115.63, 114.64, 114.56, 103.84, 61.81, 43.63; Anal. Calcd for C_22_H_17_FBrN_3_O_2_S (485.0): C, 54.33; H, 3.52; N, 8.64. Found: C, 54.62; H, 3.54; N, 8.62; EI-MS *m/z* 485.0 [M]^+^.

#### (Z)-4-(2-(2,4-dichlorophenyl)-2-(2-(4-fluorophenyl)hydrazono)ethoxy)-3-(thiophen-2-yl)-1,5-dihydro-2*H*-pyrrol-2-one (**5s**)

Yellow solid, m.p. 155–157 °C, yield 77%; IR (KBr, cm^−1^): 3431, 3255, 3103, 1672; ^1^H NMR (400 MHz, DMSO-*d*_*6*_) δ 9.88 (s, 1H, Ar–NH=N), 7.94 (s, 1H, Pyrroline-1-H), 7.67 (d, *J* = 2.0 Hz, 1H, Ar(2,4-2Cl)-3-H), 7.61 (d, *J* = 8.3 Hz, 1H, Thiophene-3-H), 7.51 (dd, *J* = 8.3, 2.0 Hz, 1H, Thiophene-5-H), 7.30 (d, *J* = 5.0 Hz, 1H, Ar(2,4-2Cl)-5-H), 7.18–7.06 (m, 4H, Ar(2,4-2Cl)-6-H, Ar(4-F)-2,3,5-3H), 7.04 (d, *J* = 3.0 Hz, 1H, Ar(4-F)-6-H), 6.85 (dd, *J* = 5.0, 3.8 Hz, 1H, Thiophene-4-H), 5.36 (s, 2H, CH_2_), 4.26 (s, 2H, Pyrroline-5-2H); ^13^C NMR (100 MHz, DMSO-*d*_6_) δ 171.81, 165.98, 158.28, 155.94, 142.19, 138.08, 135.88, 133.82, 133.74, 132.93, 132.34, 129.47, 127.85, 126.45, 124.63, 124.01, 116.19, 115.97, 114.70, 114.63, 103.99, 65.77, 43.51; Anal. Calcd for C_22_H_16_FCl_2_N_3_O_2_S (475.0): C, 55.47; H, 3.39; N, 8.82. Found: C, 55.42; H, 3.36; N, 8.76; EI-MS *m/z* 475.0 [M]^+^.

#### (Z)-4-(2-(4-methoxyphenyl)-2-(2-(4-fluorophenyl)hydrazono)ethoxy)-3-(thiophen-2-yl)-1,5-dihydro-2*H*-pyrrol-2-one (**5t**)

Yellow solid, m.p. 153–155 °C, yield 58%; IR (KBr, cm^−1^): 3419, 3251, 3067, 1677; ^1^H NMR (400 MHz, DMSO-*d*_*6*_) δ 9.91 (s, 1H, Ar–NH=N), 7.97 (s, 1H, Pyrroline-1-H), 7.80 (d, *J* = 8.8 Hz, 2H, Ar(4-OCH_3_)-2,6-2H), 7.42 (d, *J* = 3.6 Hz, 1H, Thiophene-3-H), 7.32 (d, *J* = 5.0 Hz, 1H, Thiophene-5-H), 7.22 (dd, *J* = 9.0, 4.8 Hz, 2H, Ar(4-F)-2,6-2H), 7.11 (t, *J* = 8.8 Hz, 2H, Ar(4-F)-3,5-2H), 6.99–6.92 (m, 3H, Ar(4-OCH_3_)-3,5-2H, Thiophene-4-H), 5.38 (s, 2H, CH_2_), 4.37 (s, 2H, Pyrroline-5-2H), 3.78 (s, 3H, CH_3_); ^13^C NMR (100 MHz, DMSO-*d*_6_) δ 171.97, 166.96, 159.61, 142.49, 136.88, 132.64, 130.14, 127.32, 126.77, 124.59, 124.07, 116.19, 115.97, 114.41, 114.32, 103.80, 61.89, 55.64, 43.64; Anal. Calcd for C_23_H_20_FN_3_O_2_S (437.1): C, 63.15; H, 4.61; N, 9.61. Found: C, 63.42; H, 4.63; N, 9.66; EI-MS *m/z* 437.1 [M]^+^.

#### (Z)-4-(2-(4-fluorophenyl)-2-(2-(4-fluorophenyl)hydrazono)ethoxy)-1-methyl-3-(thiophen-2-yl)-1,5-dihydro-2*H*-pyrrol-2-one (**5u**)

White solid, m.p. 147–149 °C, yield 80%; IR (KBr, cm^−1^): 3263, 2987, 1667; ^1^H NMR (400 MHz, DMSO-*d*_*6*_) δ 10.12 (s, 1H, Ar–NH=N), 7.89 (dd, *J* = 8.3, 5.7 Hz, 2H, Ar(4-F)-2,6-2H), 7.41 (d, *J* = 2.8 Hz, 1H, Thiophene-5-H), 7.32 (d, *J* = 4.9 Hz, 1H, Thiophene-3-H), 7.25 (dt, *J* = 13.7, 6.8 Hz, 4H, Ar(4-F)-3,5-2H, Ar(4-F)-2,6-2H), 7.12 (t, *J* = 8.8 Hz, 2H, Ar(4-F)-3,5-2H), 6.96–6.91 (m, 1H, Thiophene-4-H), 5.41 (s, 2H, CH_2_), 4.47 (s, 2H, Pyrroline-5-2H), 2.99 (s, 3H, CH_3_); ^13^C NMR (100 MHz, DMSO-*d*_6_) δ 169.51, 164.59, 163.57, 161.13, 158.18, 155.84, 142.29, 135.78, 134.15, 132.64, 128.01, 127.93, 126.83, 124.67, 124.06, 116.22, 116.00, 115.83, 115.62, 114.65, 114.58, 103.65, 62.08, 49.70, 29.06; Anal. Calcd for C_23_H_19_F_2_N_3_O_2_S (439.1): C, 62.86; H, 4.36; N, 9.56. Found: C, 62.51; H, 4.39; N, 9.52; EI-MS *m/z* 439.1 [M]^+^.

#### (Z)-4-(2-(4-fluorophenyl)-2-(2-(4-methylphenyl)hydrazono)ethoxy)-1-methyl-3-(thiophen-2-yl)-1,5-dihydro-2*H*-pyrrol-2-one (**5v**)

White solid, m.p. 157–159 °C, yield 53%; IR (KBr, cm^−1^): 3257, 2922, 1670; ^1^H NMR (400 MHz, DMSO-*d*_*6*_) δ 10.08 (s, 1H, Ar–NH=N), 7.76 (d, *J* = 7.7 Hz, 2H, Ar(4-CH_3_)-2,6-2H), 7.41 (s, 1H, Thiophene-5-H), 7.31 (d, *J* = 8.5 Hz, 3H, Thiophene-3-H, Ar(4-CH_3_)-3,5-2H), 7.28–7.17 (m, 4H, Ar(4-F)-2,3,5,6-4H), 6.93 (s, 1H, Thiophene-4-H), 5.37 (s, 2H, CH_2_), 4.45 (s, 2H, Pyrroline-5-2H), 2.99 (s, 3H, CH_3_), 2.32 (s, 3H, CH_3_); ^13^C NMR (100 MHz, DMSO-*d*_6_) δ 169.53, 164.71, 158.08, 155.74, 142.40, 137.58, 136.59, 134.88, 132.67, 129.49, 126.82, 125.80, 124.63, 124.07, 116.96, 116.88, 116.19, 115.97, 114.55, 114.48, 103.59, 62.06, 49.71, 29.05, 21.26; Anal. Calcd for C_24_H_22_FN_3_O_2_S (435.1): C, 66.19; H, 5.09; N, 9.65. Found: C, 66.44; H, 5.12; N, 9.71; EI-MS *m/z* 435.1 [M]^+^.

#### (Z)-4-(2-(4-methylphenyl)-2-(2-(4-methylphenyl)hydrazono)ethoxy)-1-methyl-3-(thiophen-2-yl)-1,5-dihydro-2*H*-pyrrol-2-one (**5w**)

White solid, m.p. 175–177 °C, yield 41%; IR (KBr, cm^−1^): 3230, 2988, 1668; ^1^H NMR (400 MHz, DMSO-*d*_*6*_) δ 9.99 (s, 1H, Ar–NH=N), 7.74 (d, *J* = 8.2 Hz, 2H, Ar(4-CH_3_)-3,5-2H), 7.43–7.39 (m, 1H, Thiophene-5-H), 7.31 (dd, *J* = 5.1, 0.9 Hz, 1H, Thiophene-3-H), 7.21 (d, *J* = 8.1 Hz, 2H, Ar(4-CH_3_)-2,6-2H), 7.16 (d, *J* = 8.4 Hz, 2H, Ar(4-CH_3_)-2,6-2H), 7.07 (d, *J* = 8.4 Hz, 2H, Ar(4-CH_3_)-3,5-2H), 6.93 (dd, *J* = 5.1, 3.7 Hz, 1H, Thiophene-4-H), 5.40 (s, 2H, CH_2_), 4.49 (s, 2H, Pyrroline-5-2H), 2.99 (s, 3H, CH_3_), 2.32 (s, 3H, CH_3_), 2.23 (s, 3H, CH_3_); ^13^C NMR (100 MHz, DMSO-*d*_6_) δ 169.56, 164.84, 143.52, 137.32, 135.62, 135.08, 132.70, 129.96, 129.46, 128.77, 126.82, 125.66, 124.57, 124.02, 113.48, 103.49, 62.06, 49.75, 29.06, 21.26, 20.75; Anal. Calcd for C_25_H_25_N_3_O_2_S (431.1): C, 69.58; H, 5.84; N, 9.74. Found: C, 69.36; H, 5.87; N, 9.77; EI-MS *m/z* 431.1 [M]^+^.

## Conclusions

A series of 3-(thiophen-2-yl)-1,5-dihydro-2*H*-pyrrol-2-one derivatives bearing a hydrazone group were designed, synthesized and confirmed by FT-IR, ^1^H NMR, ^13^C NMR, EI-MS, NOESY and elemental analysis. The antifungal assays indicated that some the title compounds exhibited obvious antifungal activity against *Fg*, *Rs*, *Bc* and *Cc*. Strikingly, the EC_50_ value of **5e** against *Rs* was 1.26 µg/mL, which is better than that of drazoxolon (1.77 µg/mL). Meanwhile, title compounds **5b**, **5d**, **5e**–**5g**, **5n**–**5q** and **5t** exhibited remarkable anti-*Cc* activity, with corresponding EC_50_ values reached 5.52–9.97 µg/mL, which are better than that of drazoxolon (19.46 µg/mL). These results indicated that 3-(thiophen-2-yl)-1,5-dihydro-2*H*-pyrrol-2-one derivatives containing a hydrazone group can serve as potential structural templates in the search for novel and highly efficient fungicides. Further studies on the antifungal mechanism and structural modification of 3-(thiophen-2-yl)-1,5-dihydro-2*H*-pyrrol-2-one derivatives containing a hydrazone group are currently underway.

## Additional file

**Additional file 1.** All the copies of FT-IR, ^1^H NMR, ^13^C NMR and EI-MS for title compounds **5a–5w**.
