# Supplementary material for: Design and synthesis of novel 3-(thiophen-2-yl)-1,5-dihydro-2H-pyrrol-2-one derivatives bearing a hydrazone moiety as potential fungicides
Source: Chem Cent J. 2018 Jul 17;12:83. doi: 10.1186/s13065-018-0452-z (PMC6049848; doi:10.1186/s13065-018-0452-z)
Supplement: Supplementary file 1 — Additional file 1. All the copies of FT-IR, 1H NMR, 13C NMR and EI-MS for title compounds 5a–5w. [file 13065_2018_452_MOESM1_ESM.doc]

Additional file 1

Design and synthesis of novel 3-(thiophen-2-yl)-1,5-dihydro-2*H*-pyrrol-2-one derivatives bearing a hydrazone moiety as potential fungicides

Xiaobin Wang^1^, Zhengjiao Ren^1^, Mengqi Wang^1^, Min Chen^1^, Aiming Lu^1^, Weijie Si^1,2^, Chunlong Yang^1,2,*^

**^1^** Jiangsu Key Laboratory of Pesticide Science, College of Sciences, Nanjing Agricultural University, Nanjing 210095, China

**^2^** Key Laboratory of Monitoring and Management of Crop Diseases and Pest Insects, Ministry of Agriculture, Nanjing Agricultural University, Nanjing 210095, China

^*^ Corresponding author: ycl@njau.edu.cn (Chunlong Yang).

**Table of Contents**

[Fig. S1 IR sprectrum of title compound **5a** 5](#__RefHeading___Toc13039_WPSOffice_Level1)

[Fig. S2 ^1^H NMR sprectrum of title compound **5a** 5](#__RefHeading___Toc26755_WPSOffice_Level1)

[Fig. S3 ^13^C NMR sprectrum of title compound **5a** 6](#__RefHeading___Toc3148_WPSOffice_Level1)

[Fig. S4 EI-MS sprectrum of title compound **5a** 6](#__RefHeading___Toc4752_WPSOffice_Level1)

[Fig. S5 IR sprectrum of title compound **5b** 7](#__RefHeading___Toc11611_WPSOffice_Level1)

[Fig. S6 ^1^H NMR sprectrum of title compound **5b** 7](#__RefHeading___Toc3302_WPSOffice_Level1)

[Fig. S7 ^13^C NMR sprectrum of title compound **5b** 8](#__RefHeading___Toc29807_WPSOffice_Level1)

[Fig. S8 EI-MS sprectrum of title compound **5b** 8](#__RefHeading___Toc10631_WPSOffice_Level1)

[Fig. S9 IR sprectrum of title compound **5c** 9](#__RefHeading___Toc3222_WPSOffice_Level1)

[Fig. S10 ^1^H NMR sprectrum of title compound **5c** 9](#__RefHeading___Toc19473_WPSOffice_Level1)

[Fig. S11 ^13^C NMR sprectrum of title compound **5c** 10](#__RefHeading___Toc17561_WPSOffice_Level1)

[Fig. S12 EI-MS sprectrum of title compound **5c** 10](#__RefHeading___Toc9031_WPSOffice_Level1)

[Fig. S13 IR sprectrum of title compound **5d** 11](#__RefHeading___Toc9955_WPSOffice_Level1)

[Fig. S14 ^1^H NMR sprectrum of title compound **5d** 11](#__RefHeading___Toc5069_WPSOffice_Level1)

[Fig. S15 ^13^C NMR sprectrum of title compound **5d** 12](#__RefHeading___Toc8643_WPSOffice_Level1)

[Fig. S16 EI-MS sprectrum of title compound **5d** 12](#__RefHeading___Toc1520_WPSOffice_Level1)

[Fig. S17 IR sprectrum of title compound **5e** 13](#__RefHeading___Toc19115_WPSOffice_Level1)

[Fig. S18 ^1^H NMR sprectrum of title compound **5e** 13](#__RefHeading___Toc5738_WPSOffice_Level1)

[Fig. S19 ^13^C NMR sprectrum of title compound **5e** 14](#__RefHeading___Toc23627_WPSOffice_Level1)

[Fig. S20 EI-MS sprectrum of title compound **5e** 14](#__RefHeading___Toc27264_WPSOffice_Level1)

[Fig. S21 IR sprectrum of title compound **5f** 15](#__RefHeading___Toc20165_WPSOffice_Level1)

[Fig. S22 ^1^H NMR sprectrum of title compound **5f** 15](#__RefHeading___Toc10744_WPSOffice_Level1)

[Fig. S23 ^13^C NMR sprectrum of title compound **5f** 16](#__RefHeading___Toc8783_WPSOffice_Level1)

[Fig. S24 EI-MS sprectrum of title compound **5f** 16](#__RefHeading___Toc30120_WPSOffice_Level1)

[Fig. S25 IR sprectrum of title compound **5g** 17](#__RefHeading___Toc30708_WPSOffice_Level1)

[Fig. S26 ^1^H NMR sprectrum of title compound **5g** 17](#__RefHeading___Toc10170_WPSOffice_Level1)

[Fig. S27 ^13^C NMR sprectrum of title compound **5g** 18](#__RefHeading___Toc12633_WPSOffice_Level1)

[Fig. S28 HRMS sprectrum of title compound **5g** 18](#__RefHeading___Toc21208_WPSOffice_Level1)

[Fig. S29 IR sprectrum of title compound **5h** 19](#__RefHeading___Toc22449_WPSOffice_Level1)

[Fig. S30 ^1^H NMR sprectrum of title compound **5h** 19](#__RefHeading___Toc24535_WPSOffice_Level1)

[Fig. S31 ^13^C NMR sprectrum of title compound **5h** 20](#__RefHeading___Toc19266_WPSOffice_Level1)

[Fig. S32 EI-MS sprectrum of title compound **5h** 20](#__RefHeading___Toc26013_WPSOffice_Level1)

[Fig. S33 IR sprectrum of title compound **5i** 21](#__RefHeading___Toc2091_WPSOffice_Level1)

[Fig. S34 ^1^H NMR sprectrum of title compound **5i** 21](#__RefHeading___Toc30035_WPSOffice_Level1)

[Fig. S35 ^13^C NMR sprectrum of title compound **5i** 22](#__RefHeading___Toc29264_WPSOffice_Level1)

[Fig. S36 EI-MS sprectrum of title compound **5i** 22](#__RefHeading___Toc9516_WPSOffice_Level1)

[Fig. S37 IR sprectrum of title compound **5j** 23](#__RefHeading___Toc27021_WPSOffice_Level1)

[Fig. S38 ^1^H NMR sprectrum of title compound **5j** 23](#__RefHeading___Toc13886_WPSOffice_Level1)

[Fig. S39 ^13^C NMR sprectrum of title compound **5j** 24](#__RefHeading___Toc12450_WPSOffice_Level1)

[Fig. S40 EI-MS sprectrum of title compound **5j** 24](#__RefHeading___Toc22079_WPSOffice_Level1)

[Fig. S41 IR sprectrum of title compound **5k** 25](#__RefHeading___Toc17535_WPSOffice_Level1)

[Fig. S42 ^1^H NMR sprectrum of title compound **5k** 25](#__RefHeading___Toc17451_WPSOffice_Level1)

[Fig. S43 ^13^C NMR sprectrum of title compound **5k** 26](#__RefHeading___Toc28366_WPSOffice_Level1)

[Fig. S44 EI-MS sprectrum of title compound **5k** 26](#__RefHeading___Toc5919_WPSOffice_Level1)

[Fig. S45 IR sprectrum of title compound **5l** 27](#__RefHeading___Toc30191_WPSOffice_Level1)

[Fig. S46 ^1^H NMR sprectrum of title compound **5l** 27](#__RefHeading___Toc8420_WPSOffice_Level1)

[Fig. S47 ^13^C NMR sprectrum of title compound **5l** 28](#__RefHeading___Toc7624_WPSOffice_Level1)

[Fig. S48 EI-MS sprectrum of title compound **5l** 28](#__RefHeading___Toc29186_WPSOffice_Level1)

[Fig. S49 IR sprectrum of title compound **5m** 29](#__RefHeading___Toc2065_WPSOffice_Level1)

[Fig. S50 ^1^H NMR sprectrum of title compound **5m** 29](#__RefHeading___Toc29532_WPSOffice_Level1)

[Fig. S51 ^13^C NMR sprectrum of title compound **5m** 30](#__RefHeading___Toc14342_WPSOffice_Level1)

[Fig. S52 EI-MS sprectrum of title compound **5m** 30](#__RefHeading___Toc27032_WPSOffice_Level1)

[Fig. S53 IR sprectrum of title compound **5n** 31](#__RefHeading___Toc23716_WPSOffice_Level1)

[Fig. S54 ^1^H NMR sprectrum of title compound **5n** 31](#__RefHeading___Toc6373_WPSOffice_Level1)

[Fig. S55 ^13^C NMR sprectrum of title compound **5n** 32](#__RefHeading___Toc24033_WPSOffice_Level1)

[Fig. S56 EI-MS sprectrum of title compound **5n** 32](#__RefHeading___Toc13281_WPSOffice_Level1)

[Fig. S57 IR sprectrum of title compound **5o** 33](#__RefHeading___Toc19576_WPSOffice_Level1)

[Fig. S58 ^1^H NMR sprectrum of title compound **5o** 33](#__RefHeading___Toc22179_WPSOffice_Level1)

[Fig. S59 ^13^C NMR sprectrum of title compound **5o** 34](#__RefHeading___Toc4415_WPSOffice_Level1)

[Fig. S60 EI-MS sprectrum of title compound **5o** 34](#__RefHeading___Toc5953_WPSOffice_Level1)

[Fig. S61 IR sprectrum of title compound **5p** 35](#__RefHeading___Toc24621_WPSOffice_Level1)

[Fig. S62 ^1^H NMR sprectrum of title compound **5p** 35](#__RefHeading___Toc16193_WPSOffice_Level1)

[Fig. S63 ^13^C NMR sprectrum of title compound **5p** 36](#__RefHeading___Toc21615_WPSOffice_Level1)

[Fig. S64 EI-MS sprectrum of title compound **5p** 36](#__RefHeading___Toc7638_WPSOffice_Level1)

[Fig. S65 IR sprectrum of title compound **5q** 37](#__RefHeading___Toc1597_WPSOffice_Level1)

[Fig. S66 ^1^H NMR sprectrum of title compound **5q** 37](#__RefHeading___Toc8418_WPSOffice_Level1)

[Fig. S67 ^13^C NMR sprectrum of title compound **5q** 38](#__RefHeading___Toc12372_WPSOffice_Level1)

[Fig. S68 EI-MS sprectrum of title compound **5q** 38](#__RefHeading___Toc4873_WPSOffice_Level1)

[Fig. S69 IR sprectrum of title compound **5r** 39](#__RefHeading___Toc27197_WPSOffice_Level1)

[Fig. S70 ^1^H NMR sprectrum of title compound **5r** 39](#__RefHeading___Toc3931_WPSOffice_Level1)

[Fig. S71 ^13^C NMR sprectrum of title compound **5r** 40](#__RefHeading___Toc26054_WPSOffice_Level1)

[Fig. S72 EI-MS sprectrum of title compound **5r** 40](#__RefHeading___Toc32611_WPSOffice_Level1)

[Fig. S73 IR sprectrum of title compound **5s** 41](#__RefHeading___Toc20831_WPSOffice_Level1)

[Fig. S74 ^1^H NMR sprectrum of title compound **5s** 41](#__RefHeading___Toc24223_WPSOffice_Level1)

[Fig. S75 ^13^C NMR sprectrum of title compound **5s** 42](#__RefHeading___Toc20021_WPSOffice_Level1)

[Fig. S76 EI-MS sprectrum of title compound **5s** 42](#__RefHeading___Toc5795_WPSOffice_Level1)

[Fig. S77 IR sprectrum of title compound **5t** 43](#__RefHeading___Toc8252_WPSOffice_Level1)

[Fig. S78 ^1^H NMR sprectrum of title compound **5t** 43](#__RefHeading___Toc8570_WPSOffice_Level1)

[Fig. S79 ^13^C NMR sprectrum of title compound **5t** 44](#__RefHeading___Toc12175_WPSOffice_Level1)

[Fig. S80 EI-MS sprectrum of title compound **5t** 44](#__RefHeading___Toc19469_WPSOffice_Level1)

[Fig. S81 IR sprectrum of title compound **5u** 45](#__RefHeading___Toc28112_WPSOffice_Level1)

[Fig. S82 ^1^H NMR sprectrum of title compound **5u** 45](#__RefHeading___Toc131_WPSOffice_Level1)

[Fig. S83 ^13^C NMR sprectrum of title compound **5u** 46](#__RefHeading___Toc1892_WPSOffice_Level1)

[Fig. S84 EI-MS sprectrum of title compound **5u** 46](#__RefHeading___Toc260_WPSOffice_Level1)

[Fig. S85 IR sprectrum of title compound **5v** 47](#__RefHeading___Toc18374_WPSOffice_Level1)

[Fig. S86 ^1^H NMR sprectrum of title compound **5v** 47](#__RefHeading___Toc32076_WPSOffice_Level1)

[Fig. S87 ^13^C NMR sprectrum of title compound **5v** 48](#__RefHeading___Toc4425_WPSOffice_Level1)

[Fig. S88 EI-MS sprectrum of title compound **5v** 48](#__RefHeading___Toc475_WPSOffice_Level1)

[Fig. S89 IR sprectrum of title compound **5w** 49](#__RefHeading___Toc3062_WPSOffice_Level1)

[Fig. S90 ^1^H NMR sprectrum of title compound **5w** 49](#__RefHeading___Toc30171_WPSOffice_Level1)

[Fig. S91 ^13^C NMR sprectrum of title compound **5w** 50](#__RefHeading___Toc3194_WPSOffice_Level1)

[Fig. S92 EI-MS sprectrum of title compound **5w** 50](#__RefHeading___Toc17641_WPSOffice_Level1)

Fig. S1 IR sprectrum of title compound **5a**

**
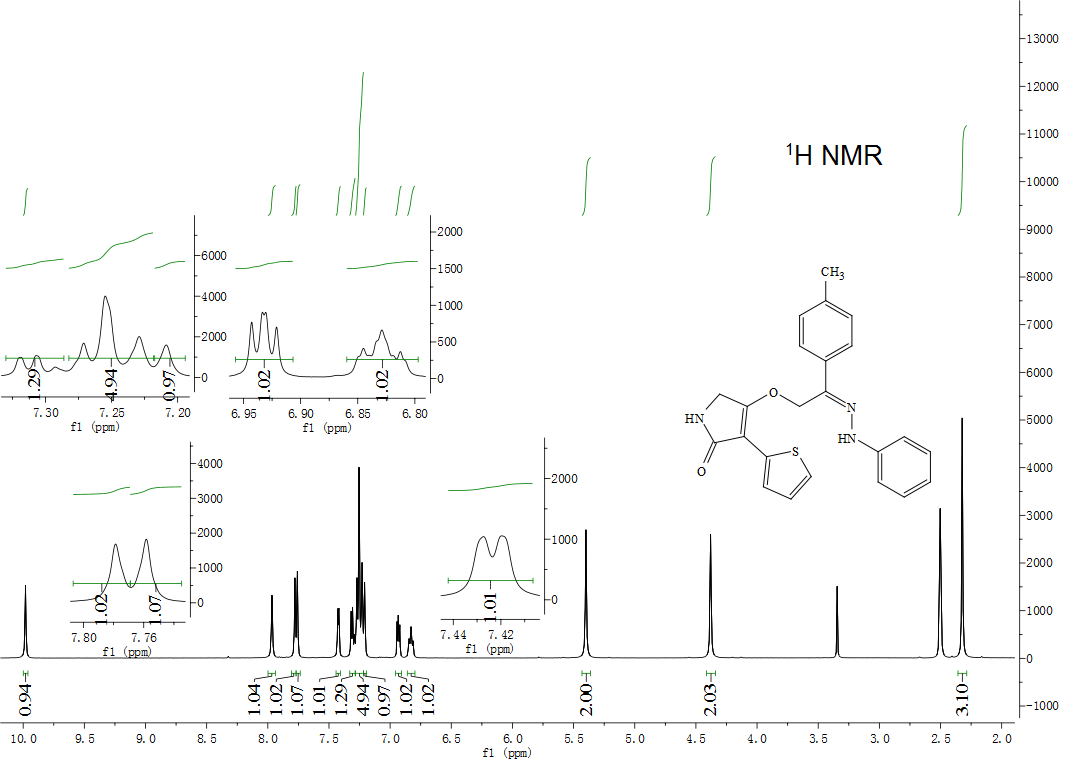
**

Fig. S2 ^1^H NMR sprectrum of title compound **5a**


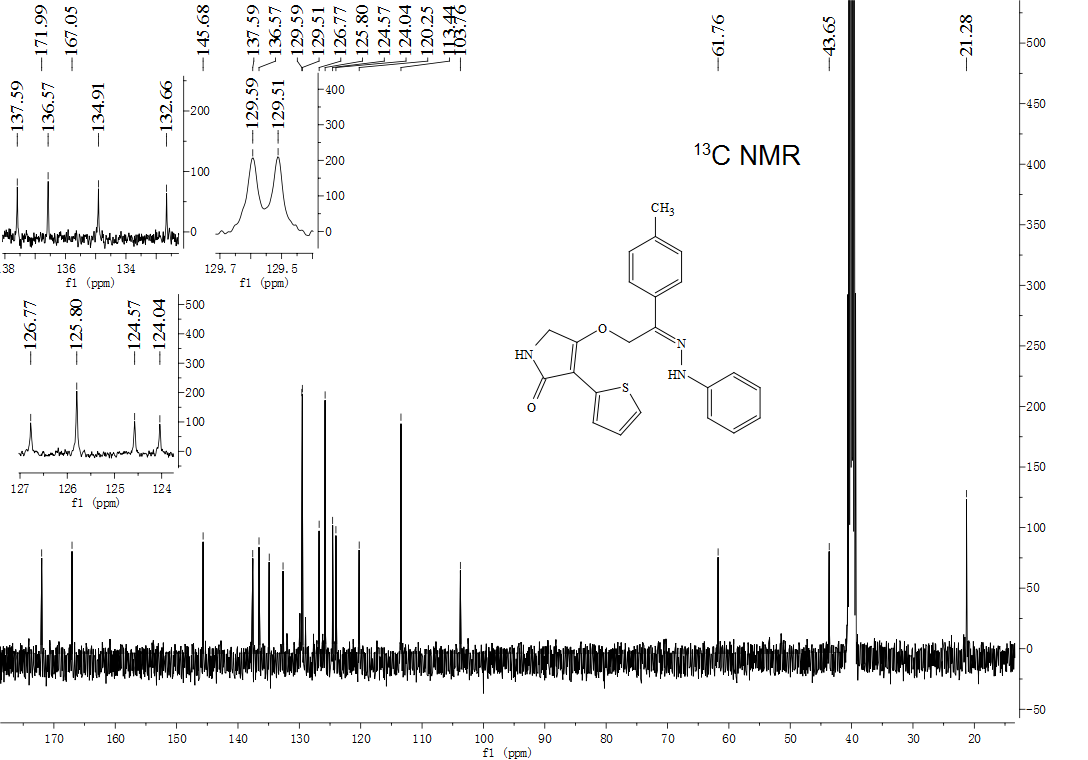


Fig. S3 ^13^C NMR sprectrum of title compound **5a**


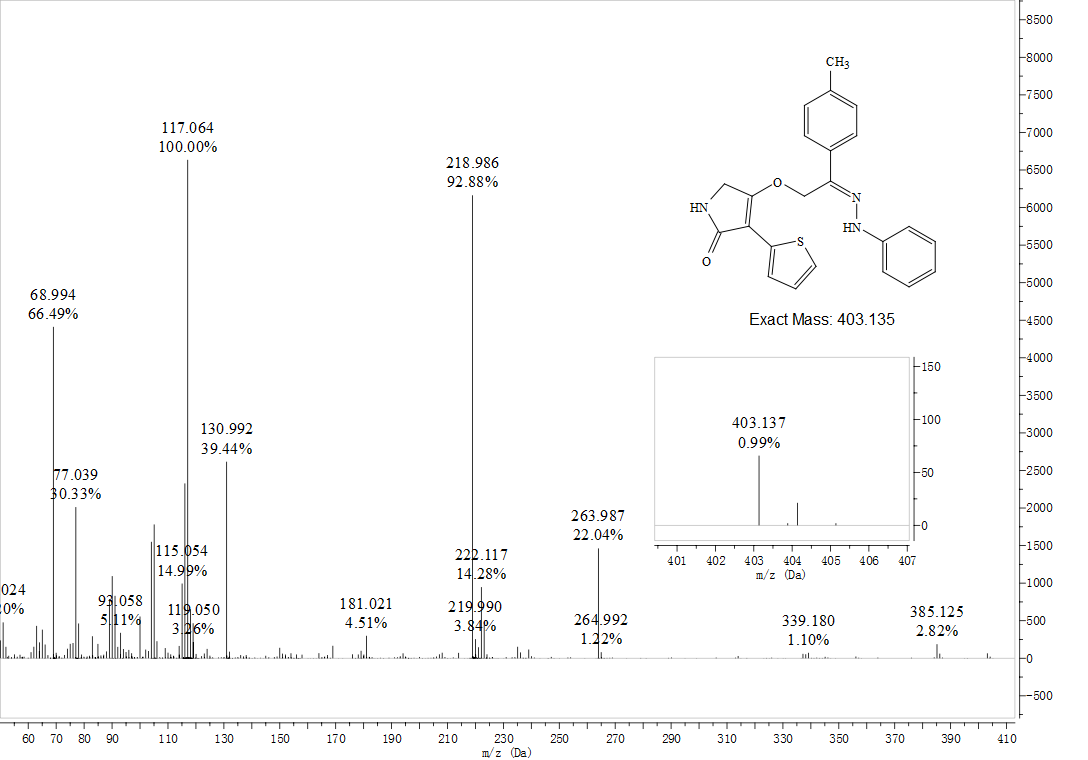


Fig. S4 EI-MS sprectrum of title compound **5a**

Fig. S5 IR sprectrum of title compound **5b**

**
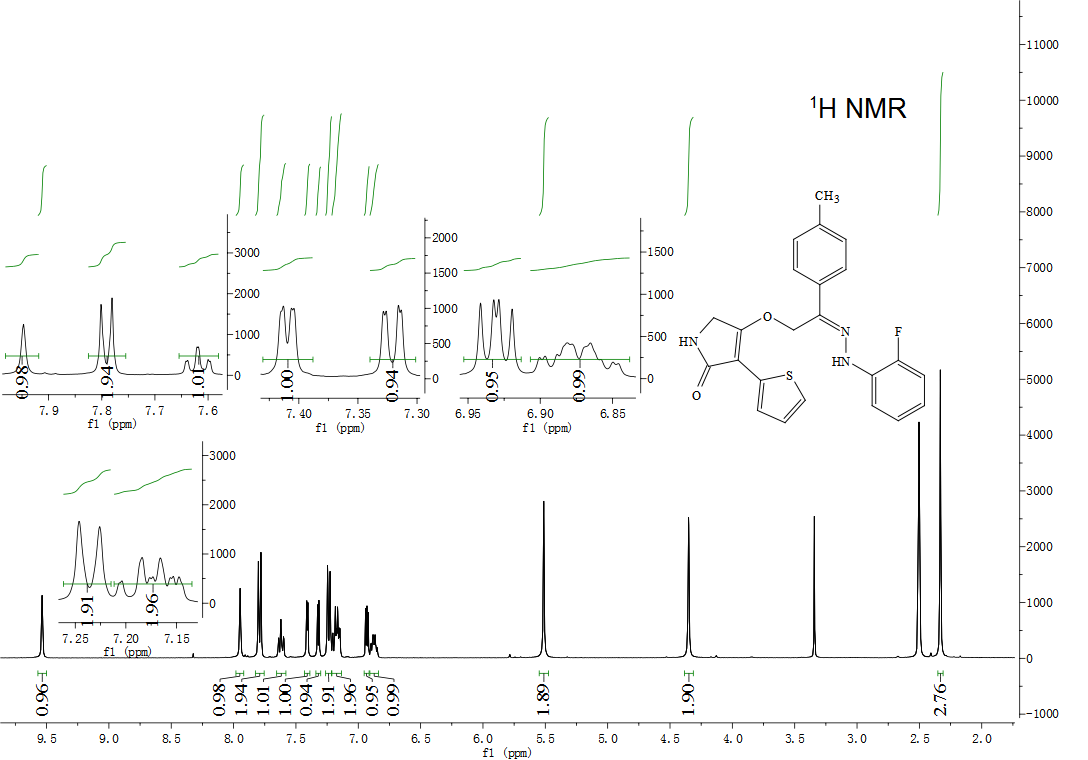
**

Fig. S6 ^1^H NMR sprectrum of title compound **5b**

**
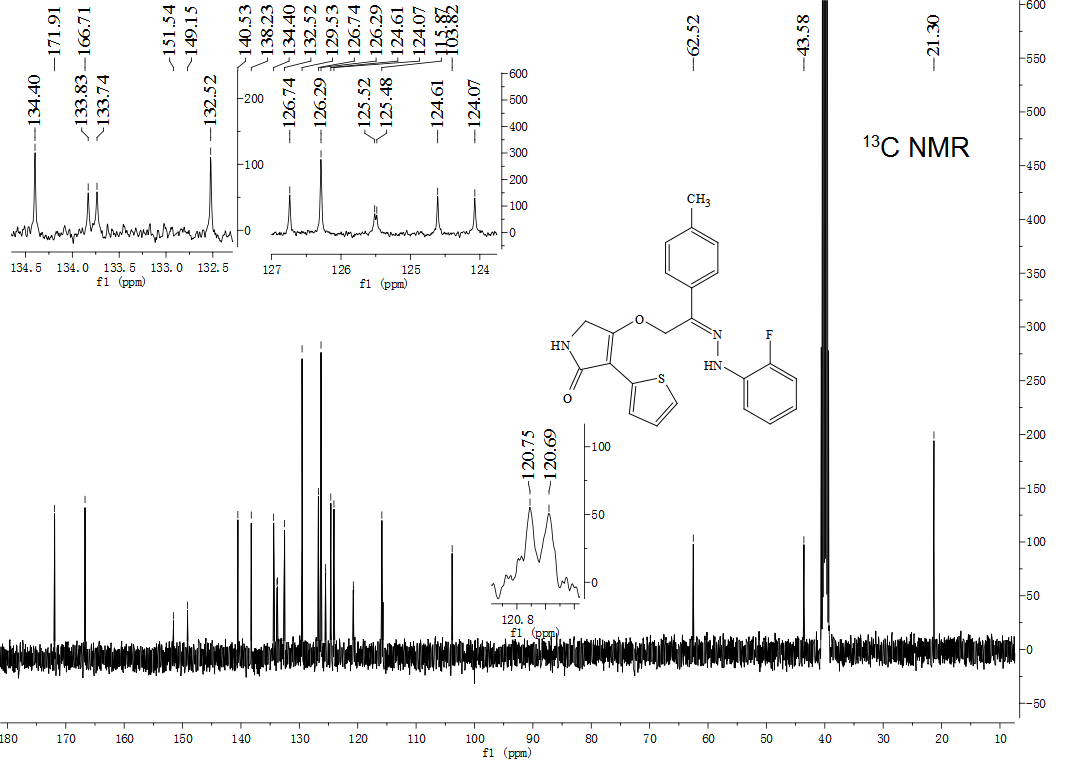
**

Fig. S7 ^13^C NMR sprectrum of title compound **5b**

**
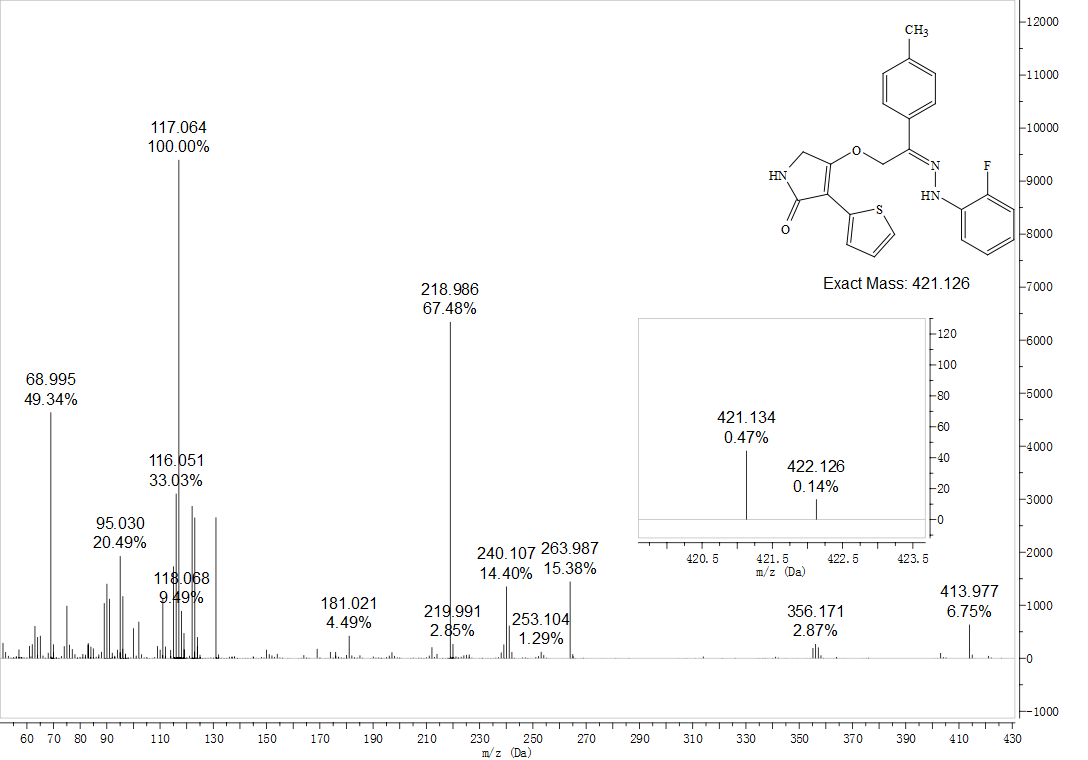
**

Fig. S8 EI-MS sprectrum of title compound **5b**

Fig. S9 IR sprectrum of title compound **5c**

**
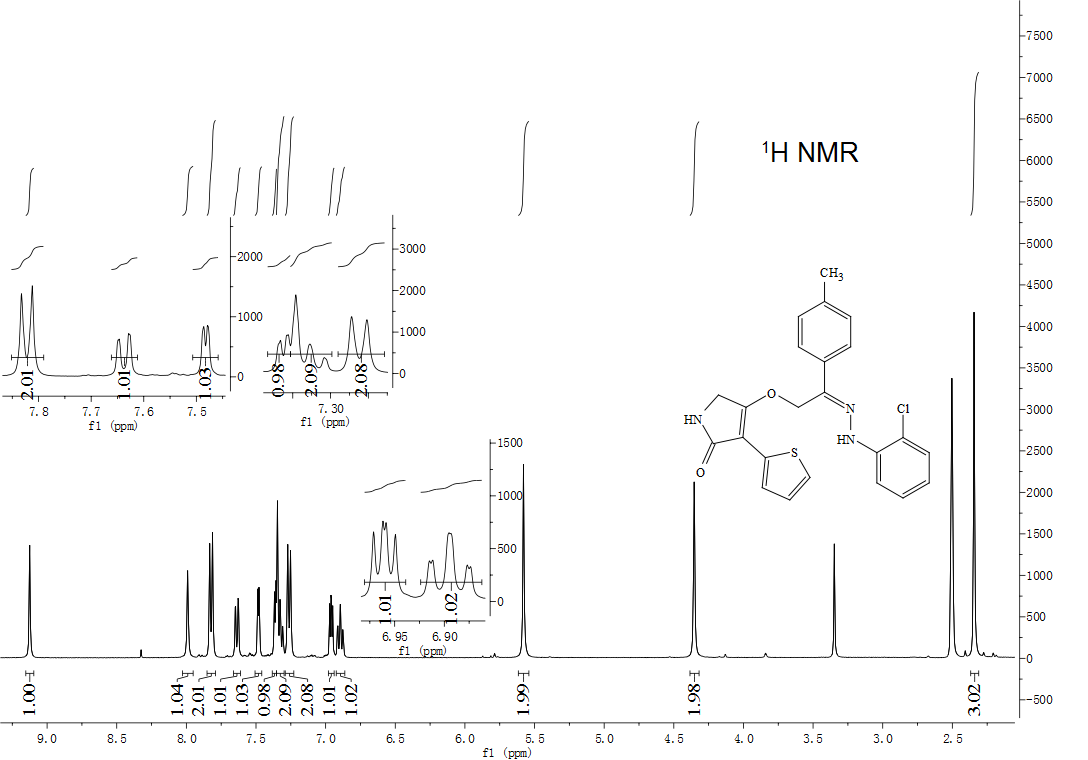
**

Fig. S10 ^1^H NMR sprectrum of title compound **5c**

**
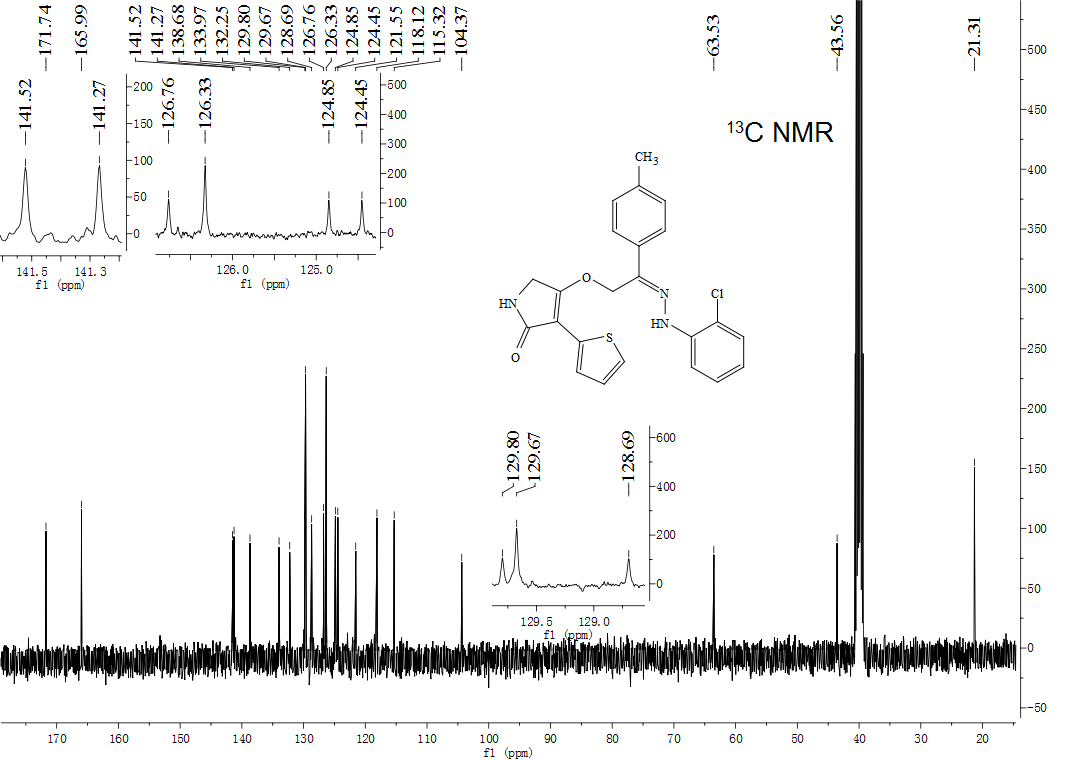
**

Fig. S11 ^13^C NMR sprectrum of title compound **5c**

**
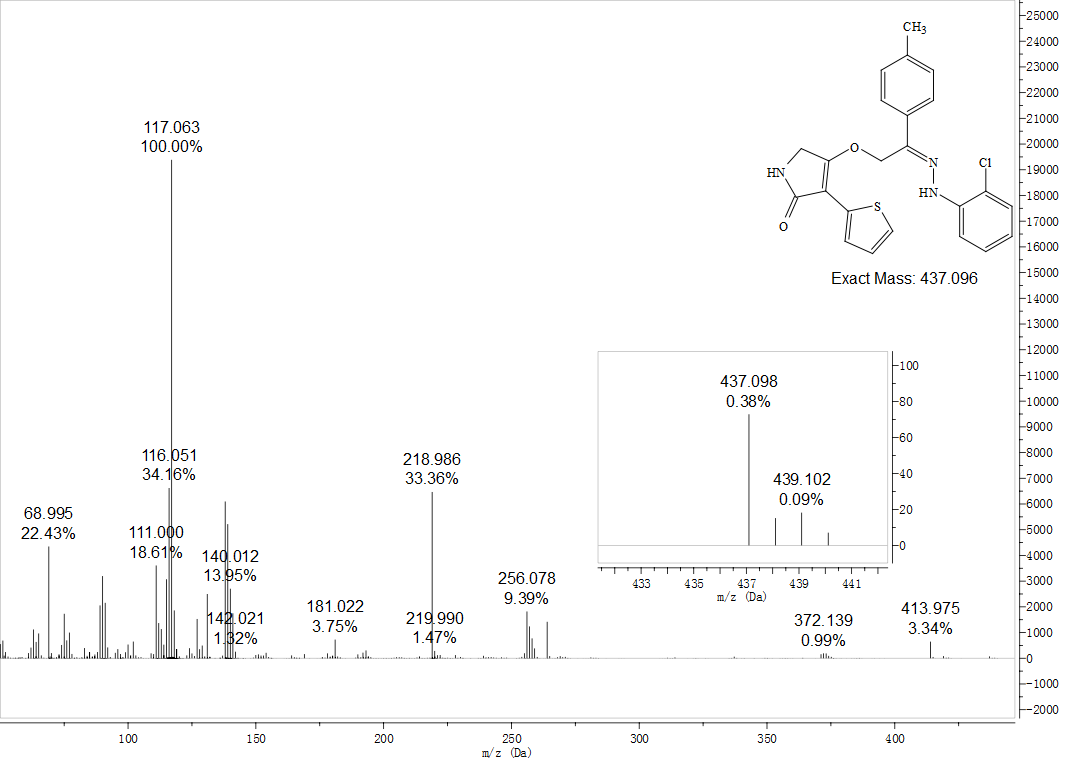
**

Fig. S12 EI-MS sprectrum of title compound **5c**

Fig. S13 IR sprectrum of title compound **5d**

**
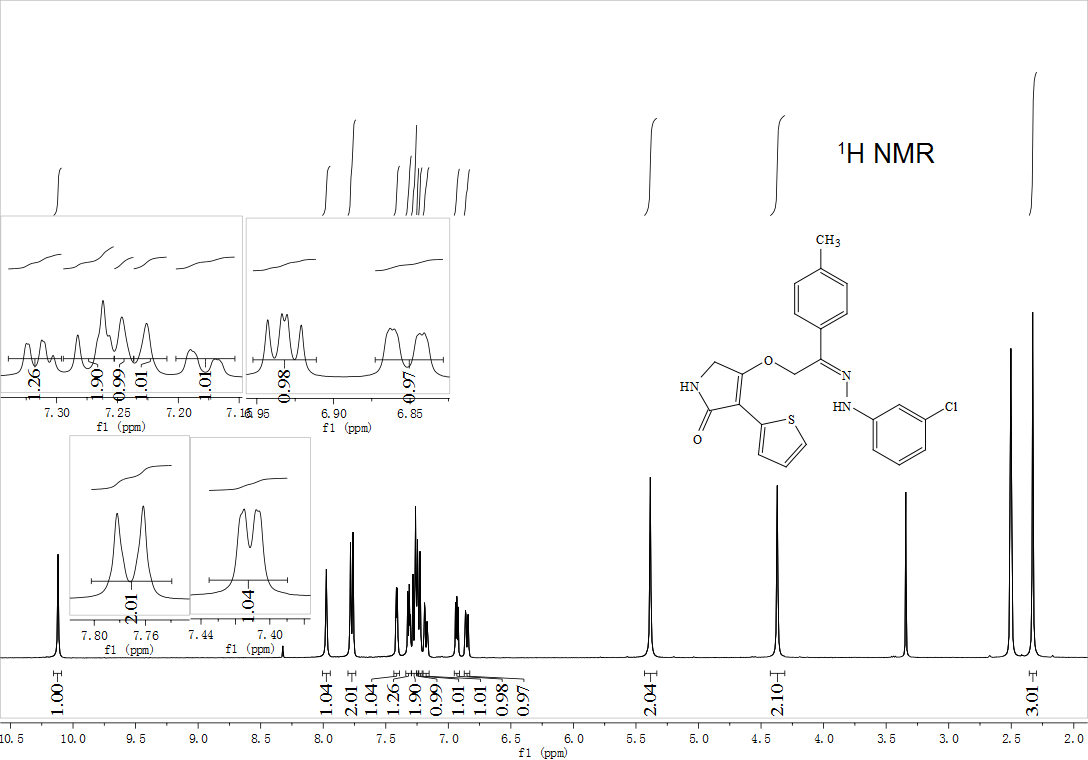
**

Fig. S14 ^1^H NMR sprectrum of title compound **5d**

**
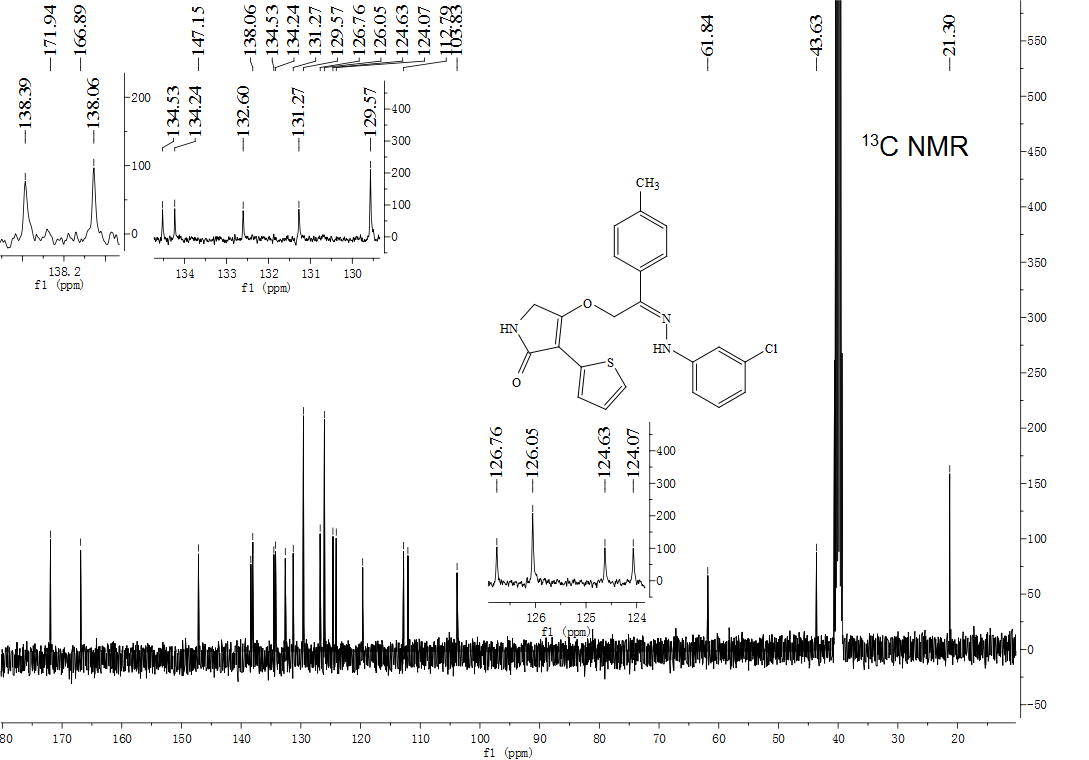
**

Fig. S15 ^13^C NMR sprectrum of title compound **5d**

**
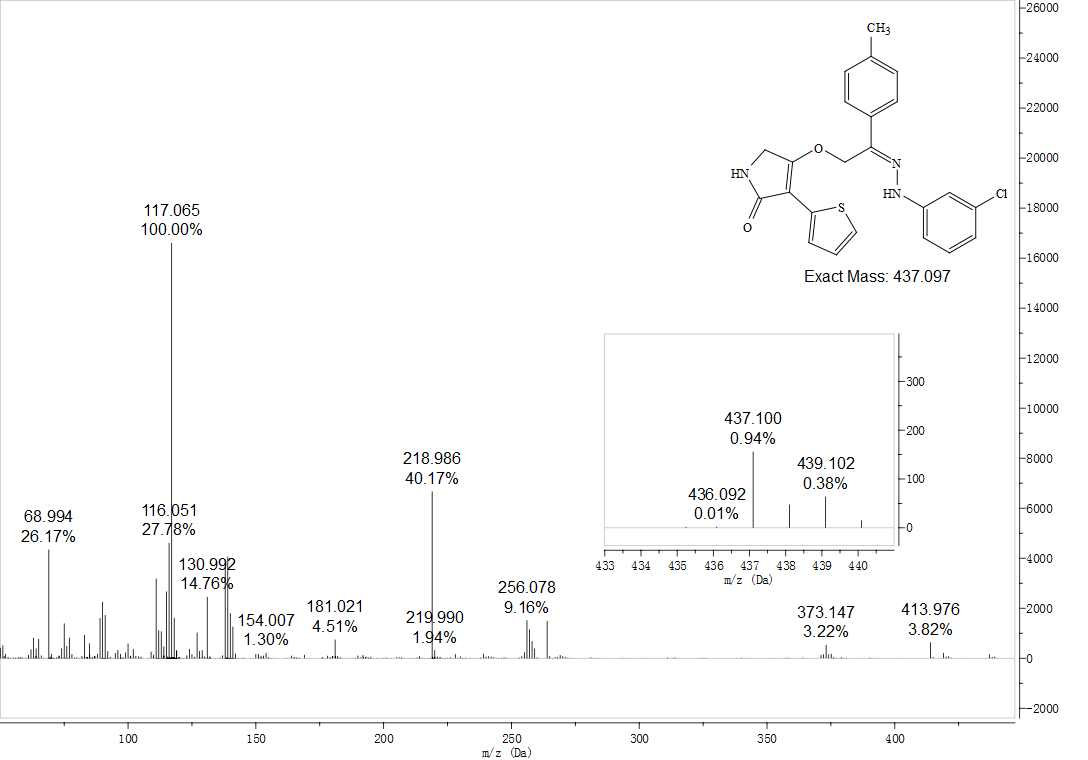
**

Fig. S16 EI-MS sprectrum of title compound **5d**

Fig. S17 IR sprectrum of title compound **5e**

**
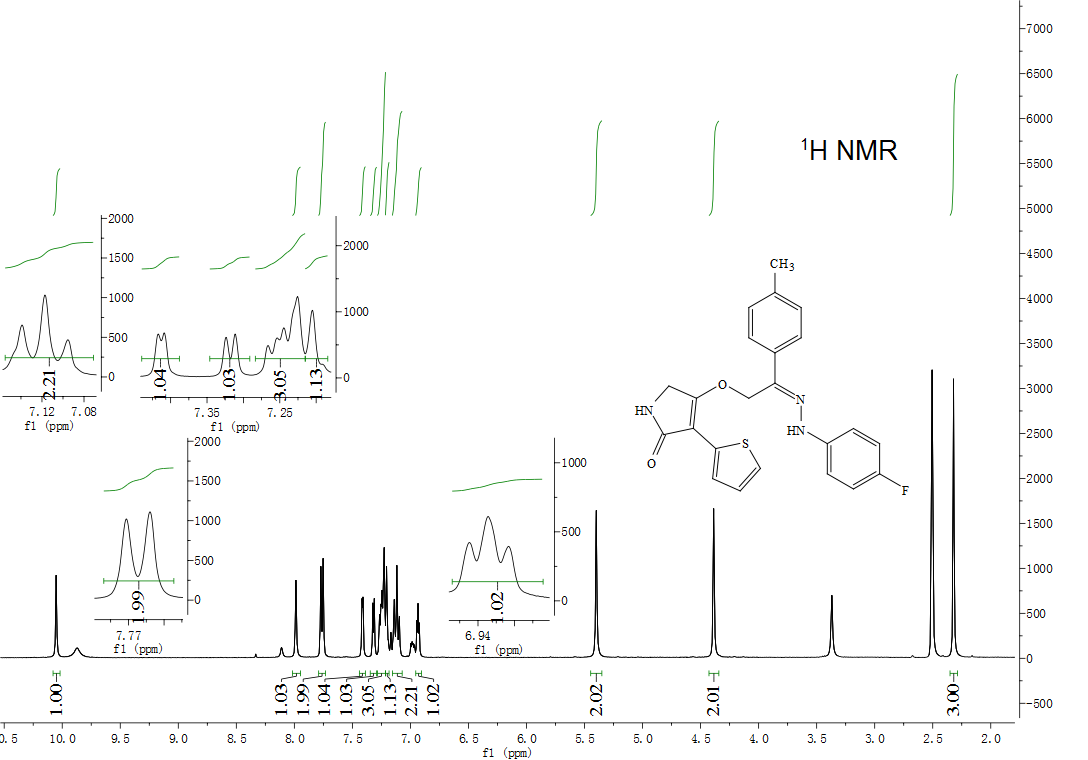
**

Fig. S18 ^1^H NMR sprectrum of title compound **5e**

**
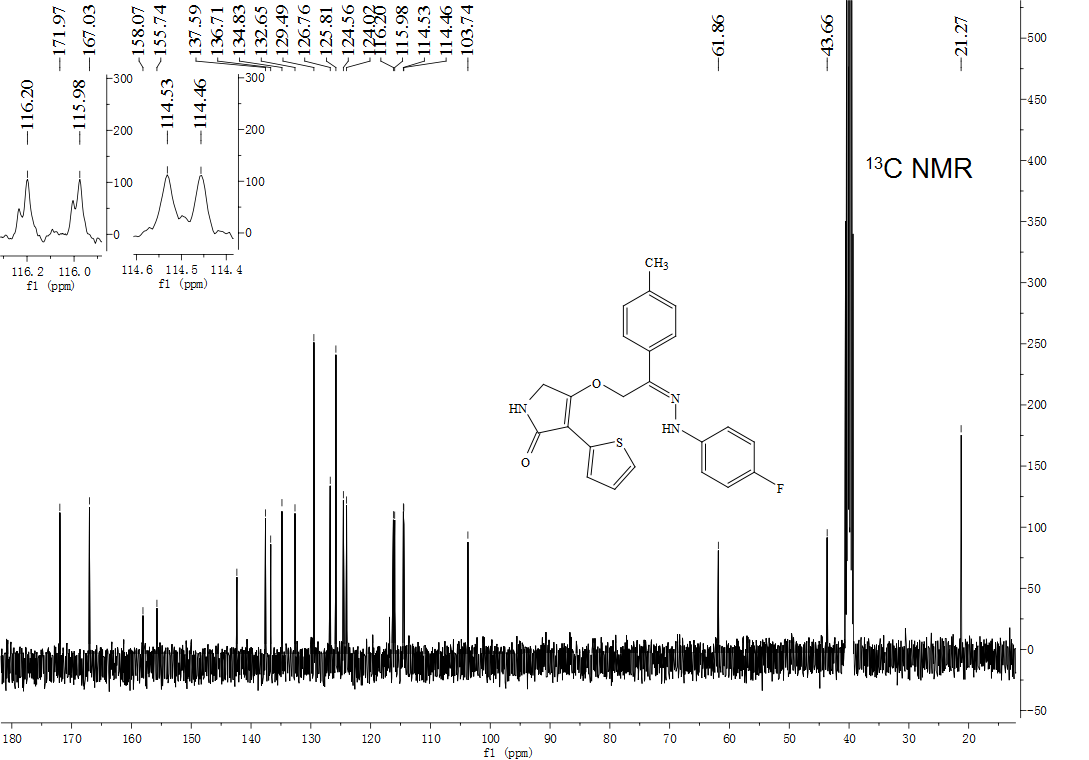
**

Fig. S19 ^13^C NMR sprectrum of title compound **5e**

**
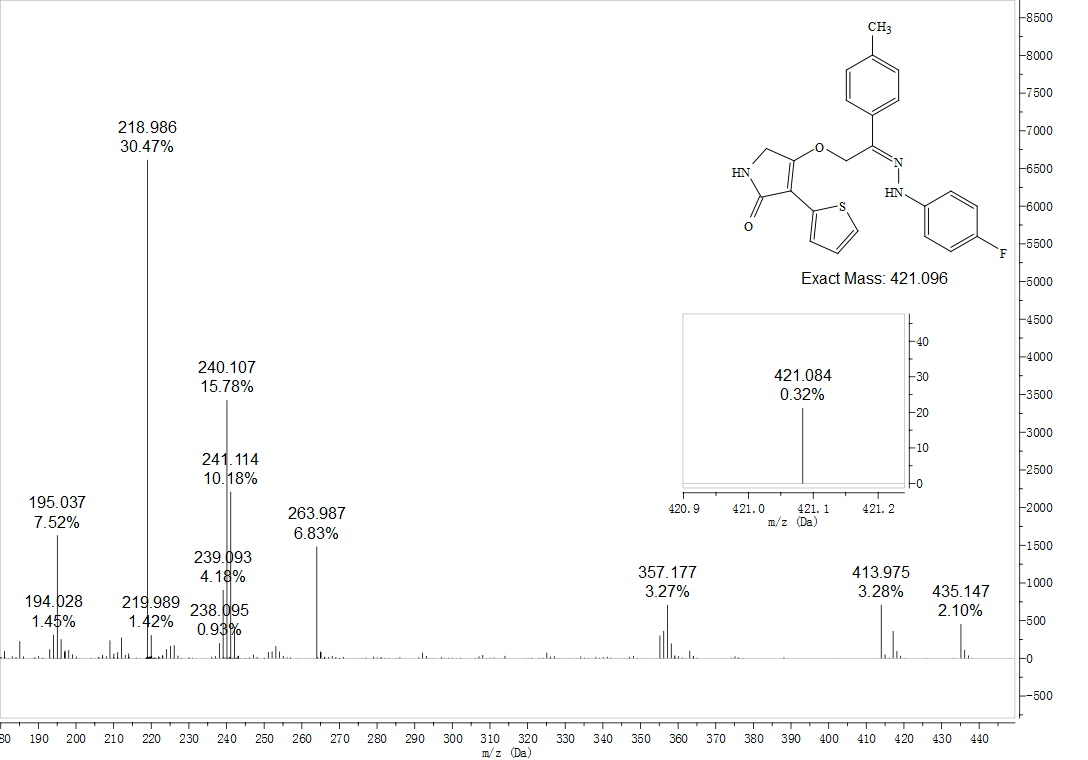
**

Fig. S20 EI-MS sprectrum of title compound **5e**

Fig. S21 IR sprectrum of title compound **5f**

**
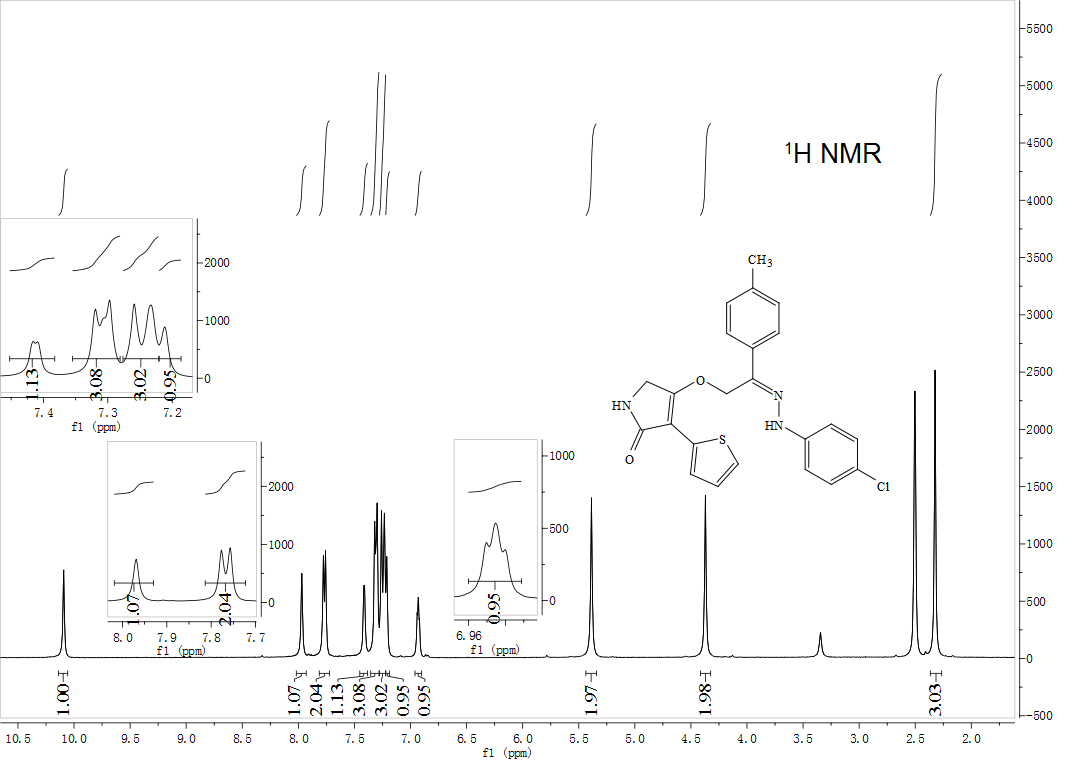
**

Fig. S22 ^1^H NMR sprectrum of title compound **5f**

**
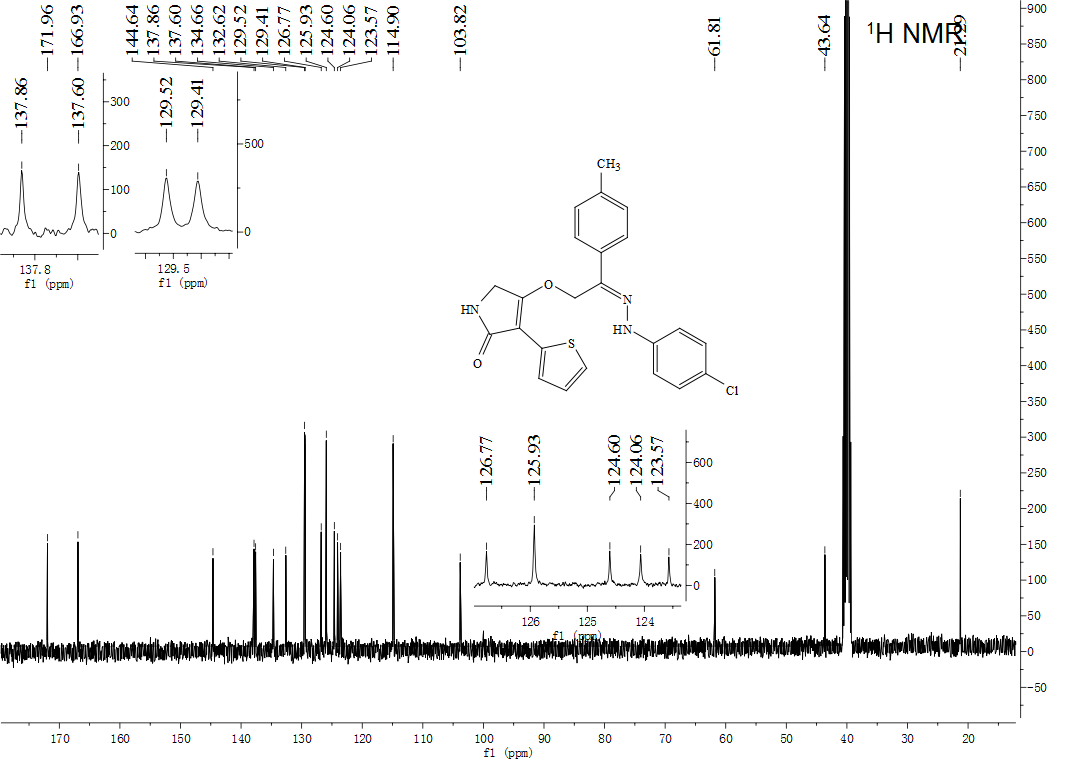
**

Fig. S23 ^13^C NMR sprectrum of title compound **5f**

**
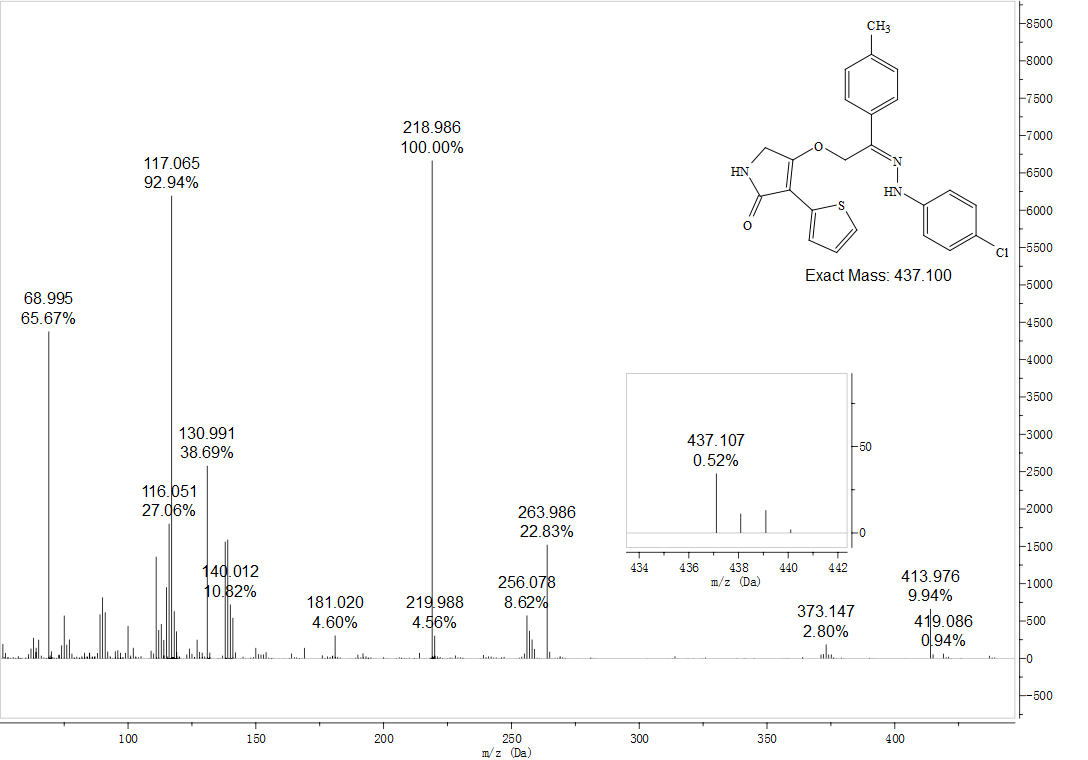
**

Fig. S24 EI-MS sprectrum of title compound **5f**

Fig. S25 IR sprectrum of title compound **5g**

**
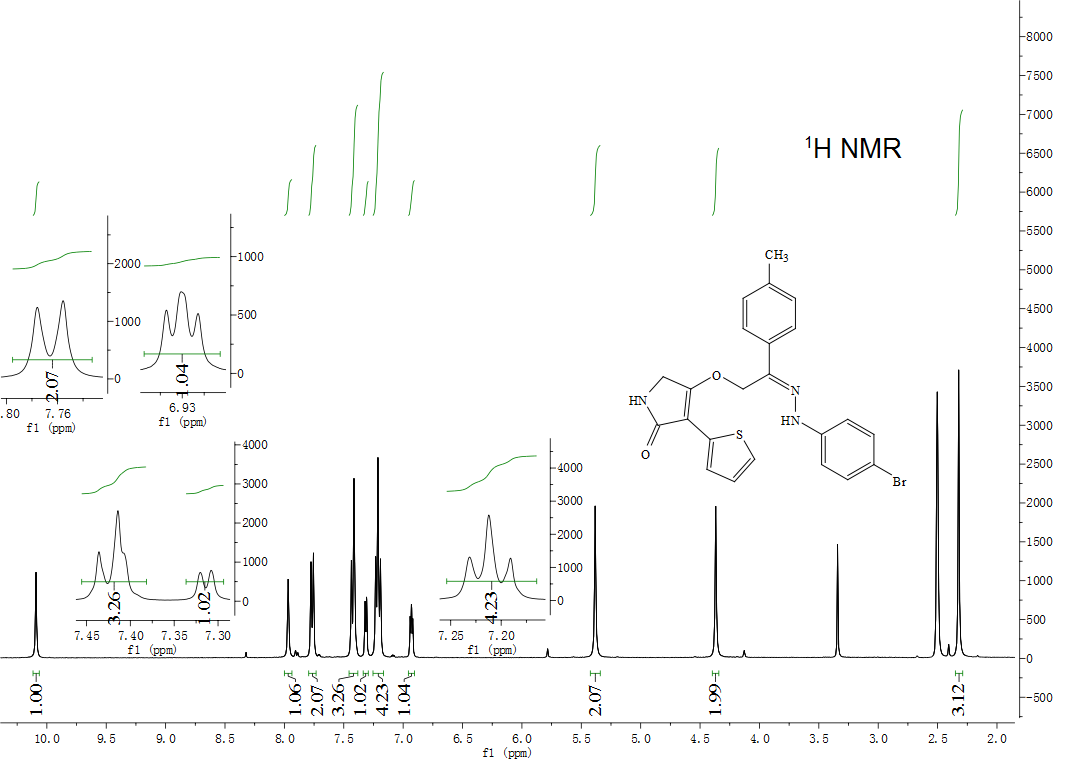
**

Fig. S26 ^1^H NMR sprectrum of title compound **5g**

**
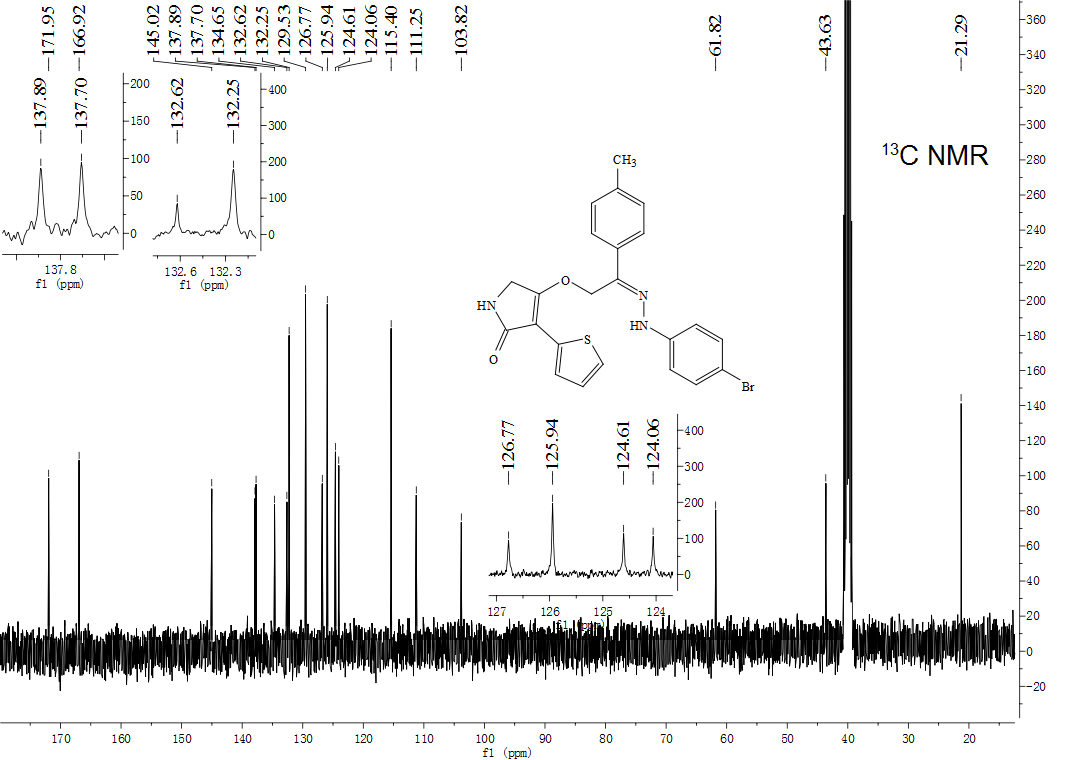
**

Fig. S27 ^13^C NMR sprectrum of title compound **5g**

**
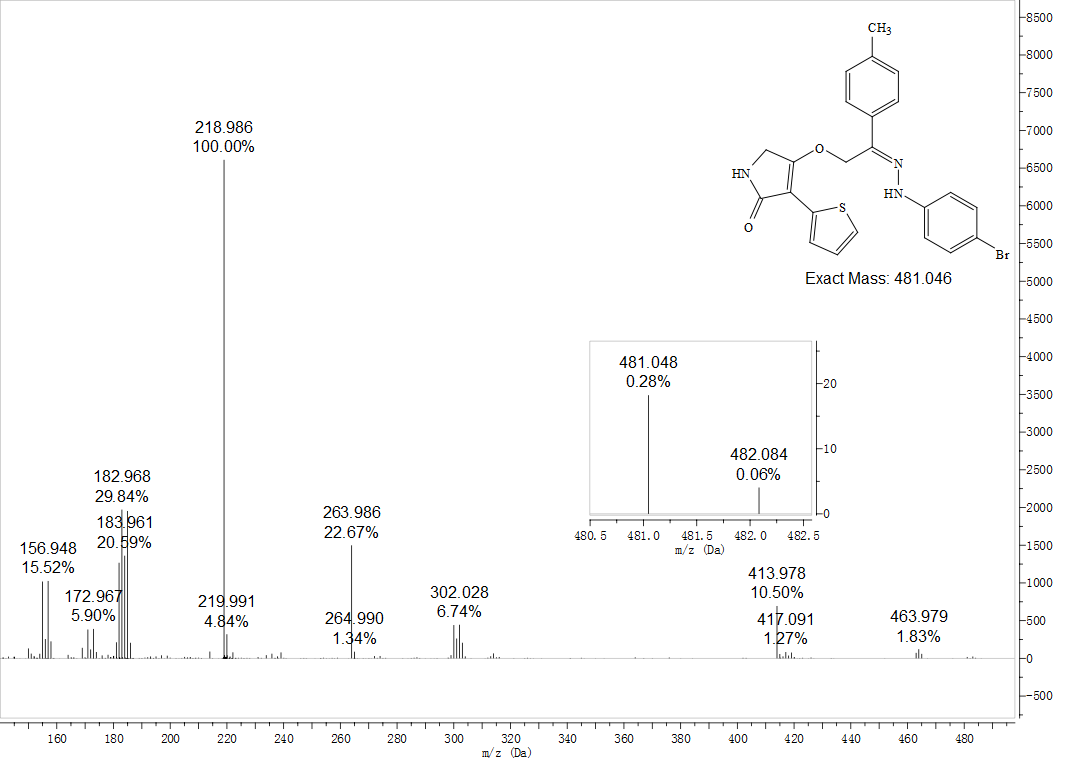
**

Fig. S28 HRMS sprectrum of title compound **5g**

Fig. S29 IR sprectrum of title compound **5h**

**
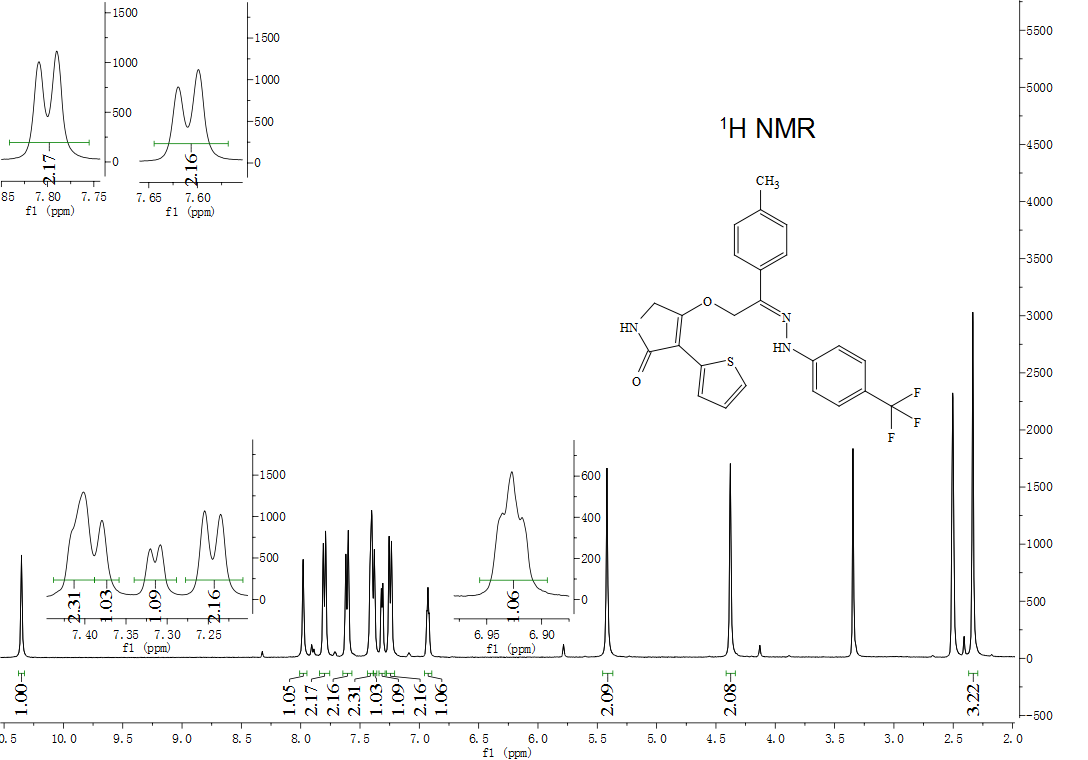
**

Fig. S30 ^1^H NMR sprectrum of title compound **5h**

**
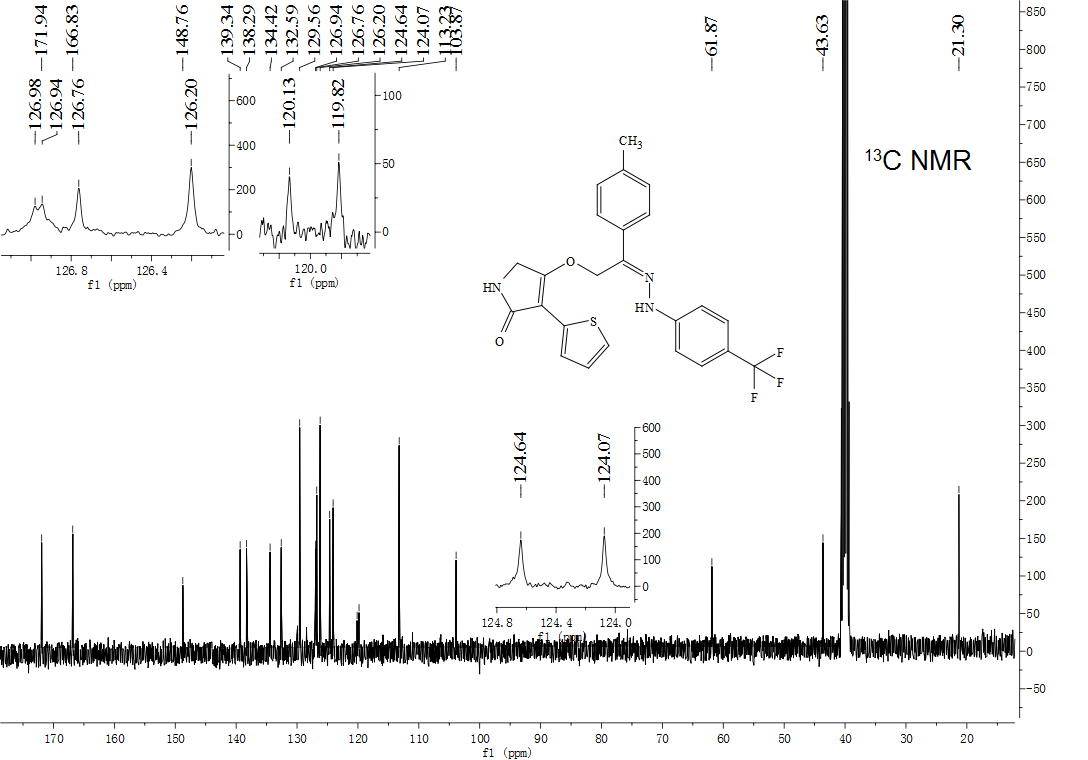
**

Fig. S31 ^13^C NMR sprectrum of title compound **5h**

**
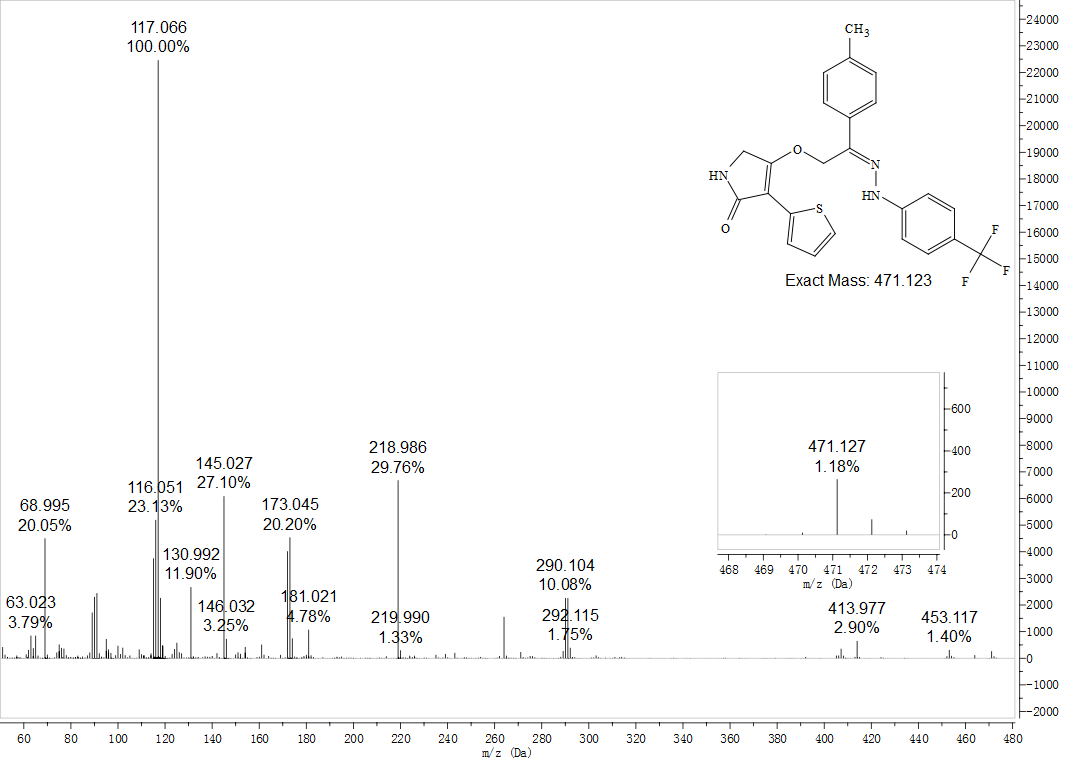
**

Fig. S32 EI-MS sprectrum of title compound **5h**

Fig. S33 IR sprectrum of title compound **5i**

**
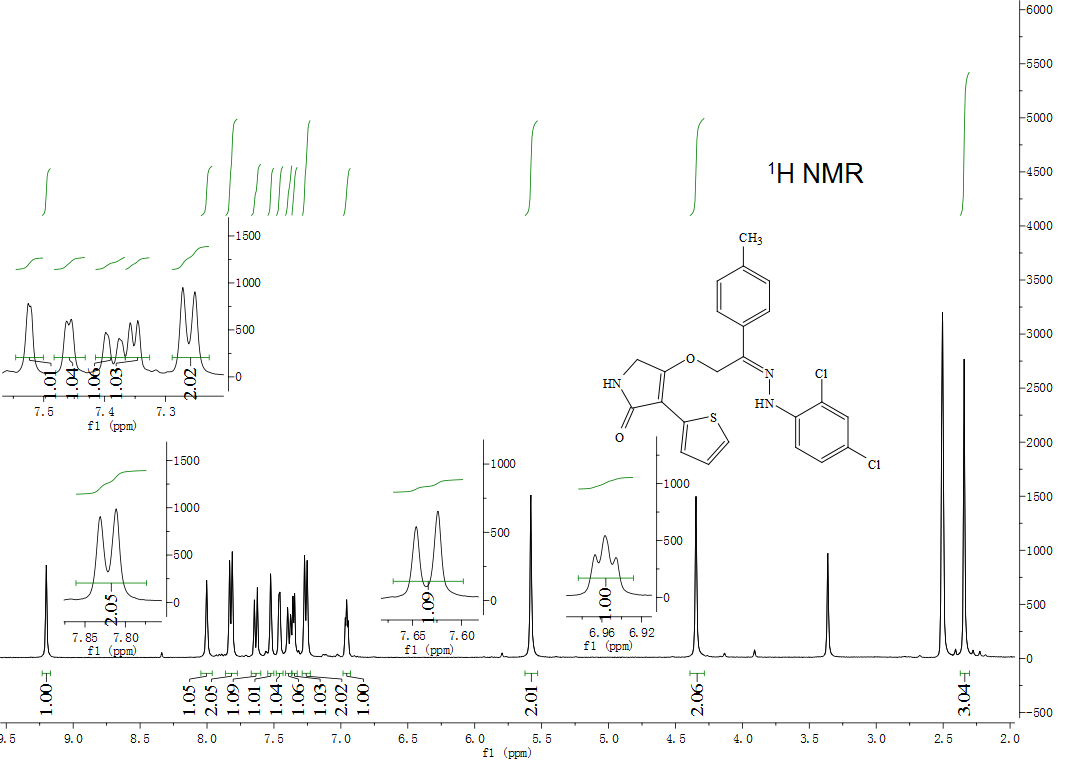
**

Fig. S34 ^1^H NMR sprectrum of title compound **5i**

**
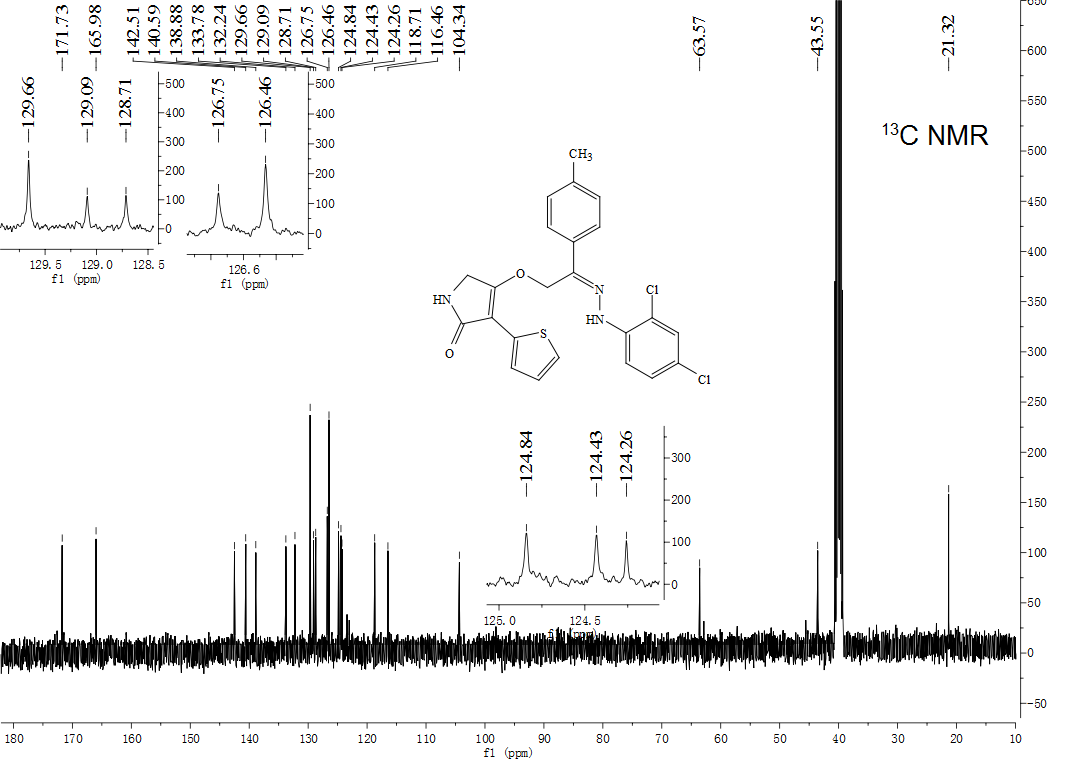
**

Fig. S35 ^13^C NMR sprectrum of title compound **5i**

**
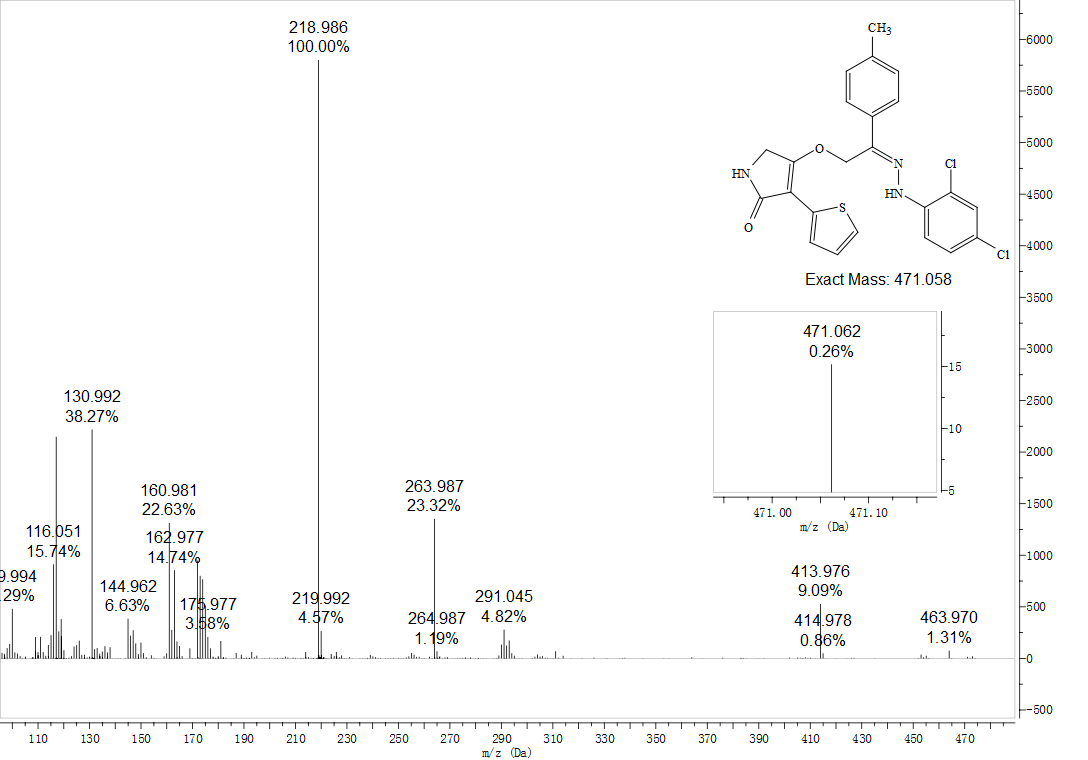
**

Fig. S36 EI-MS sprectrum of title compound **5i**

Fig. S37 IR sprectrum of title compound **5j**

**
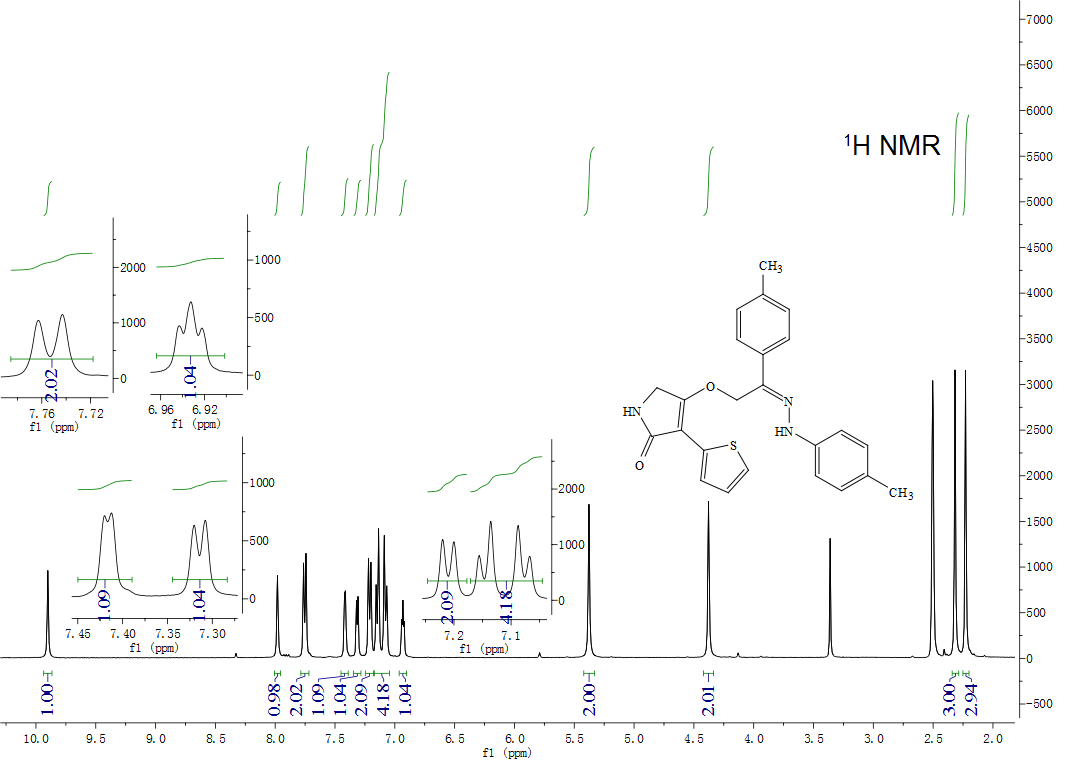
**

Fig. S38 ^1^H NMR sprectrum of title compound **5j**

**
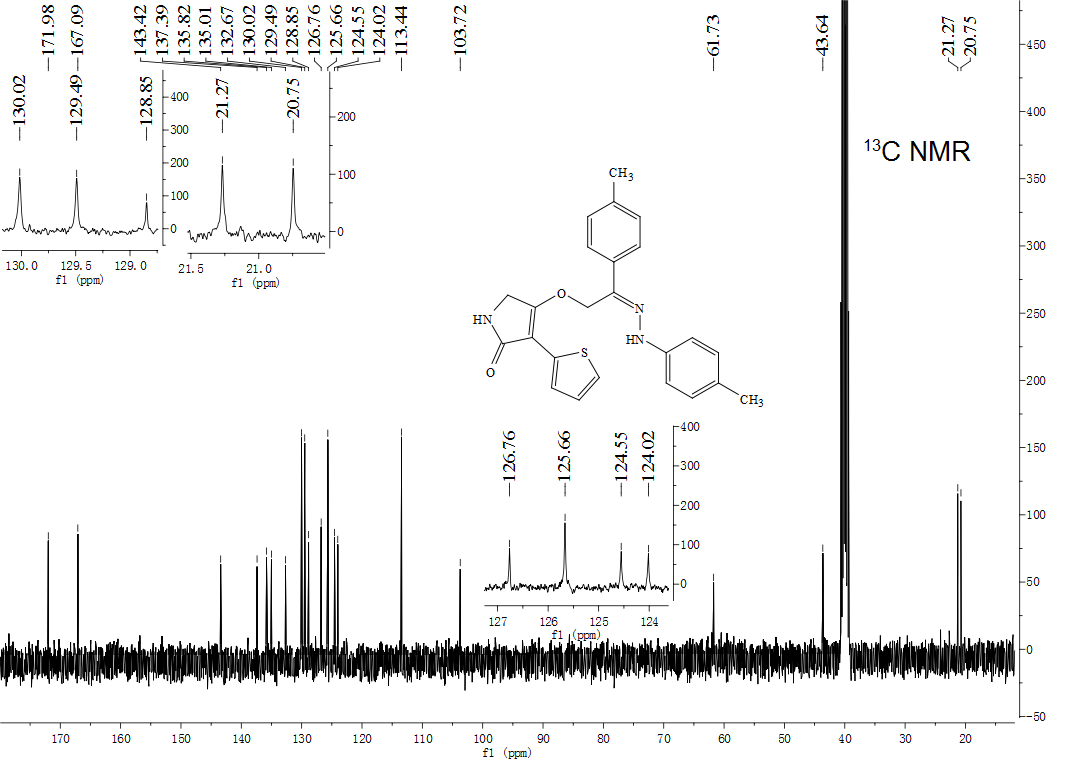
**

Fig. S39 ^13^C NMR sprectrum of title compound **5j**

**
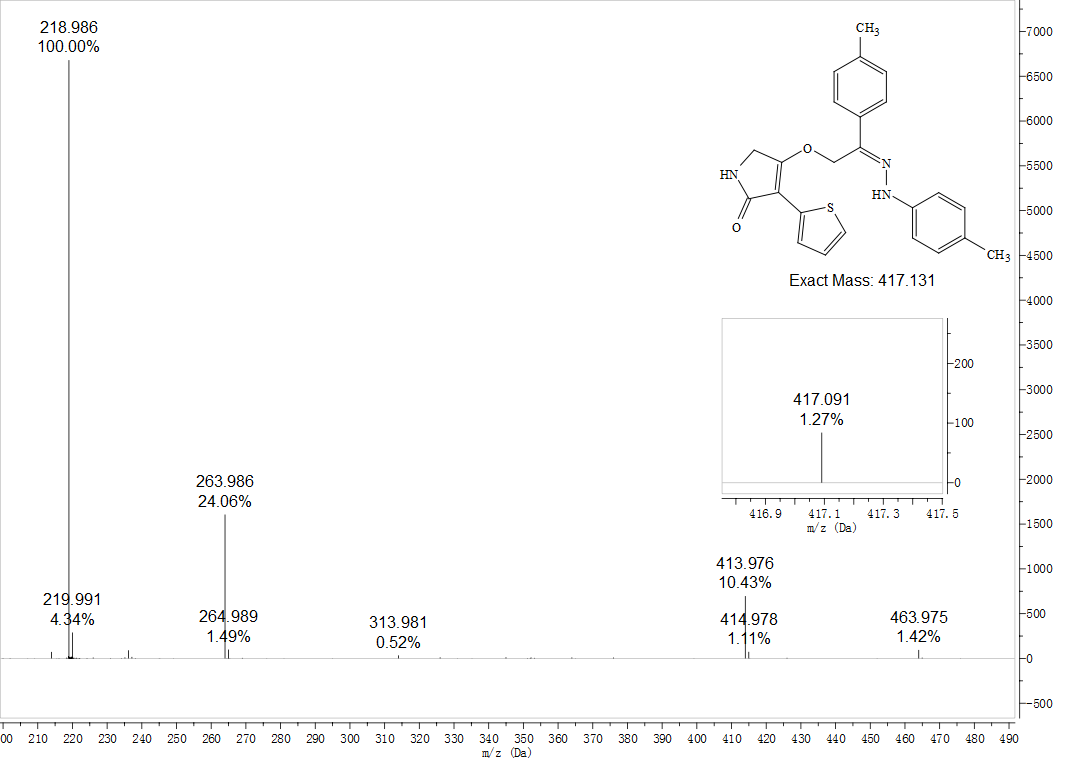
**

Fig. S40 EI-MS sprectrum of title compound **5j**

Fig. S41 IR sprectrum of title compound **5k**

**
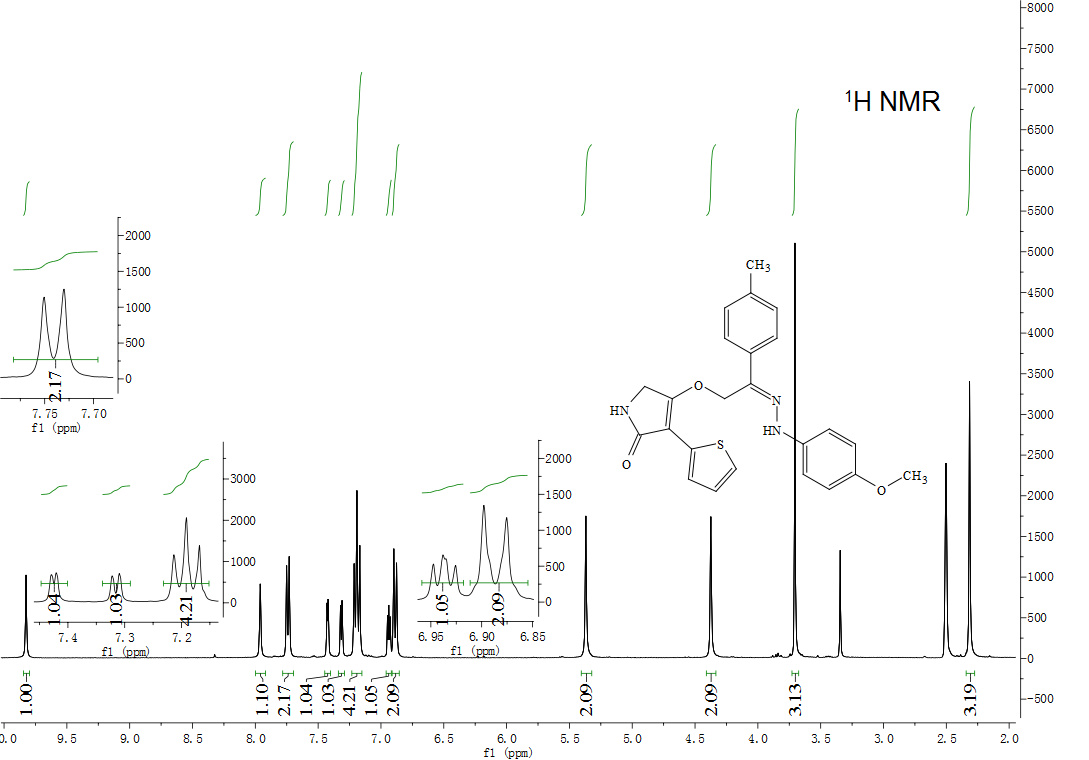
**

Fig. S42 ^1^H NMR sprectrum of title compound **5k**

**
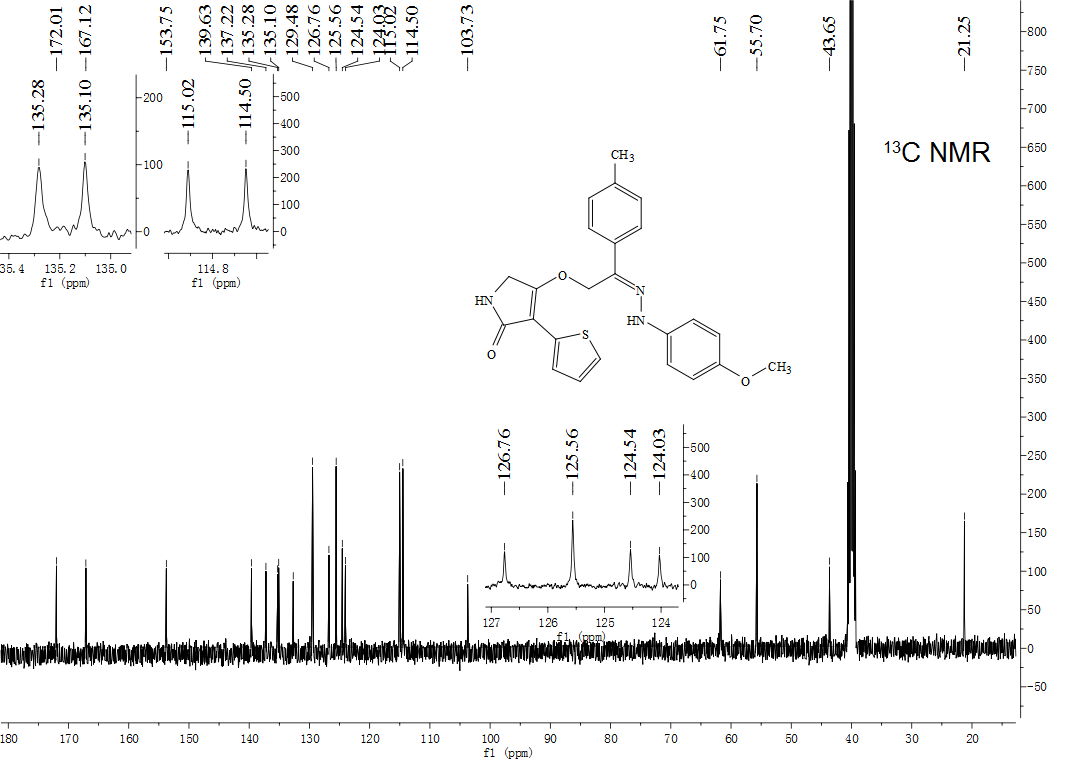
**

Fig. S43 ^13^C NMR sprectrum of title compound **5k**

**
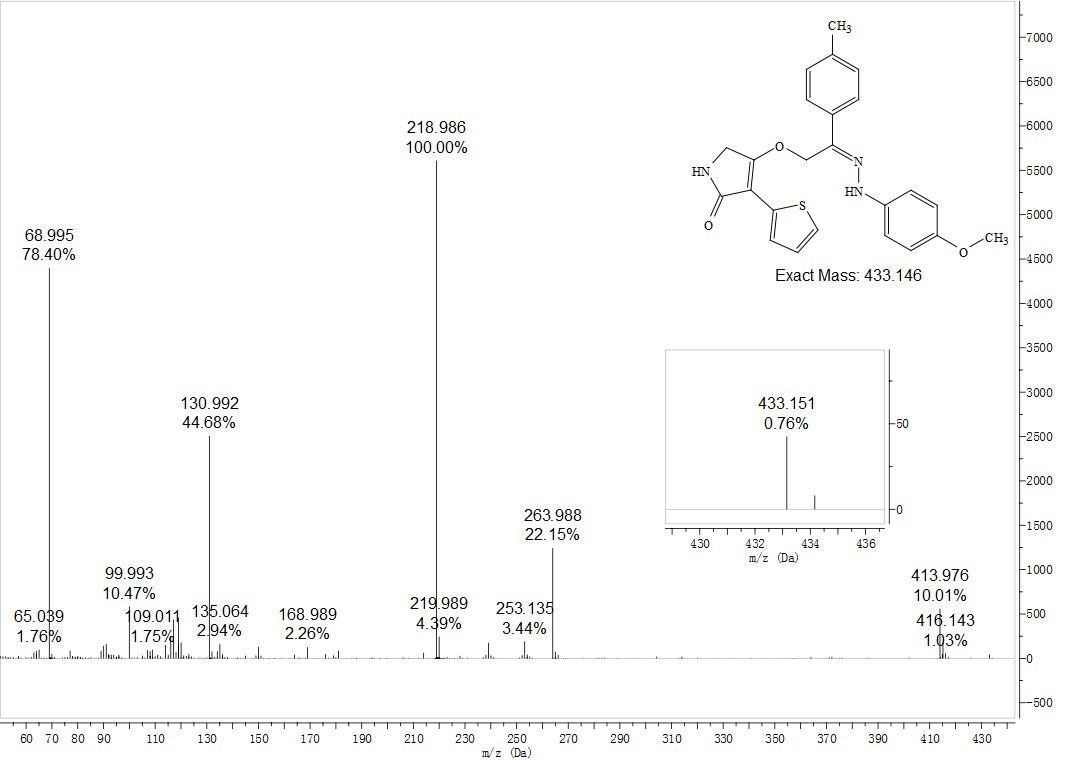
**

Fig. S44 EI-MS sprectrum of title compound **5k**

Fig. S45 IR sprectrum of title compound **5l**

**
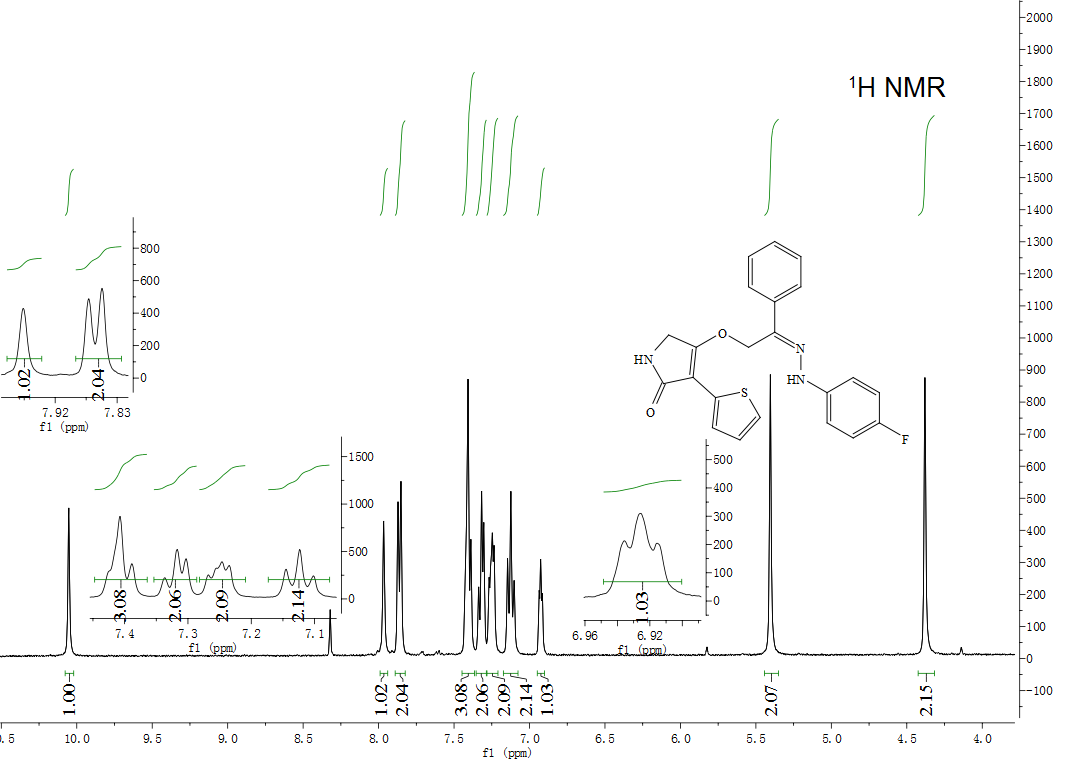
**

Fig. S46 ^1^H NMR sprectrum of title compound **5l**

**
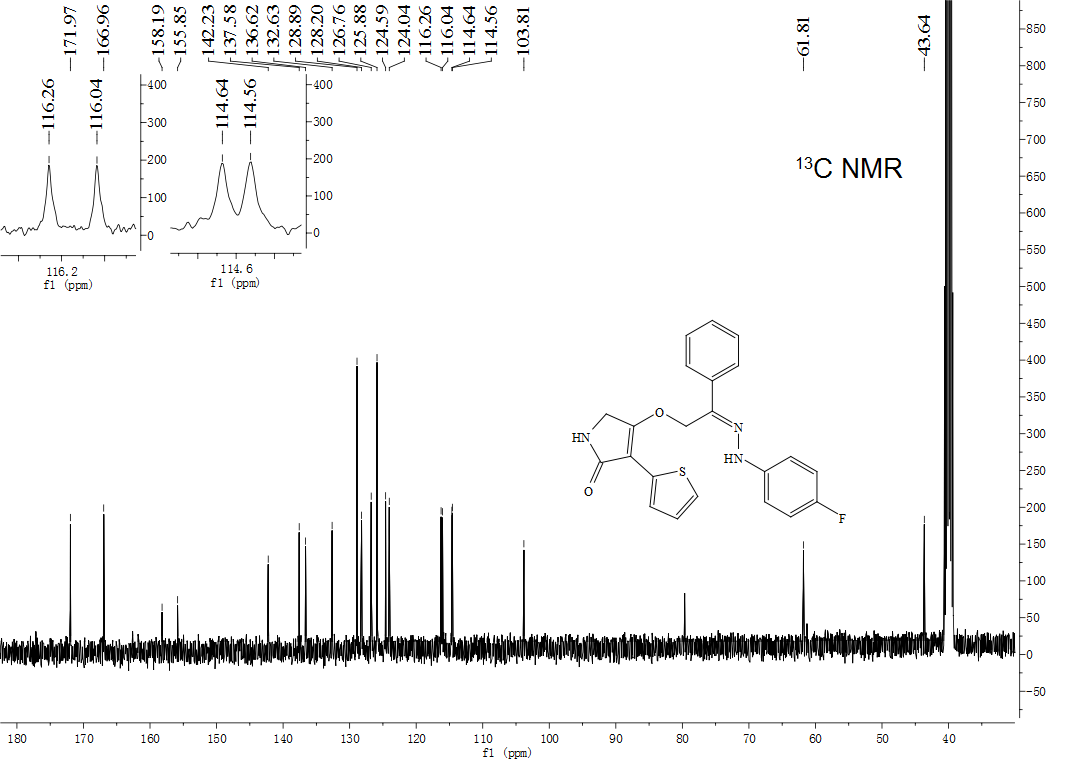
**

Fig. S47 ^13^C NMR sprectrum of title compound **5l**

**
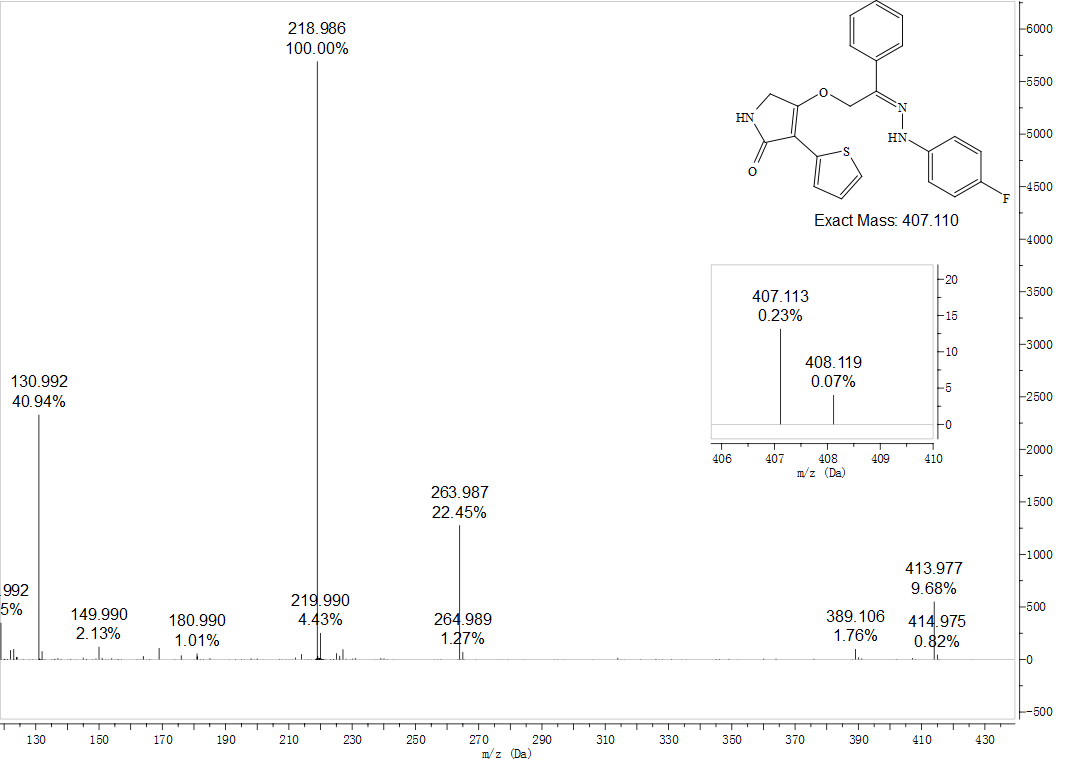
**

Fig. S48 EI-MS sprectrum of title compound **5l**

Fig. S49 IR sprectrum of title compound **5m**

**
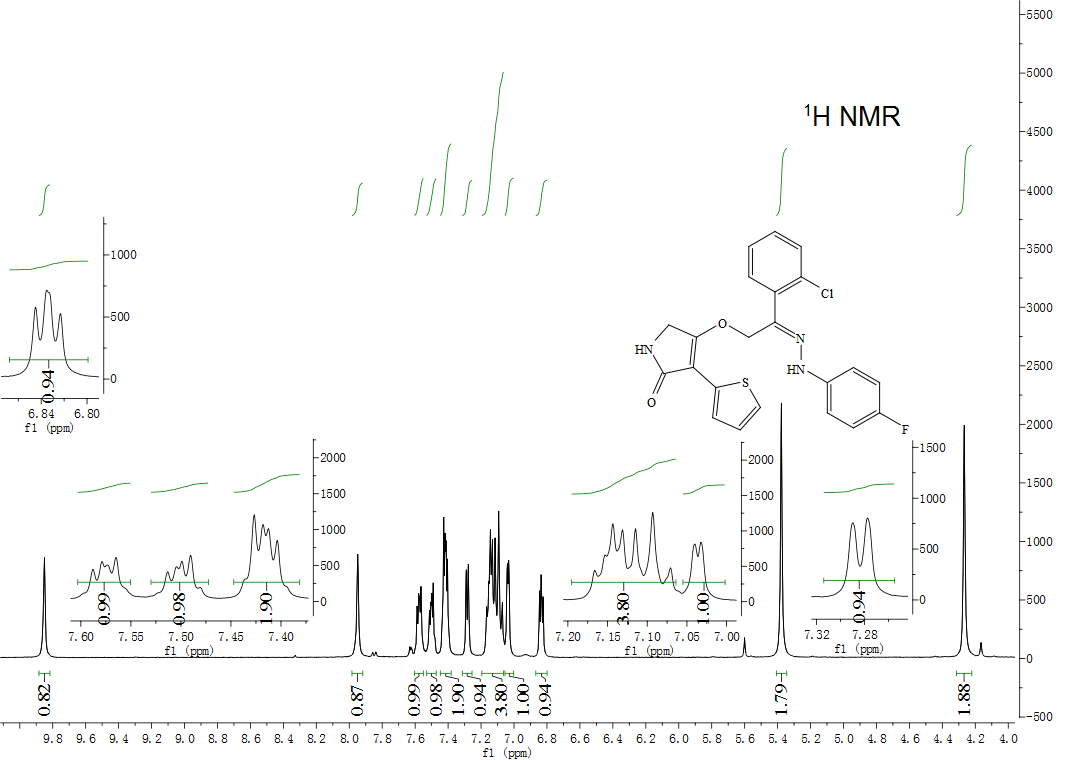
**

Fig. S50 ^1^H NMR sprectrum of title compound **5m**

**
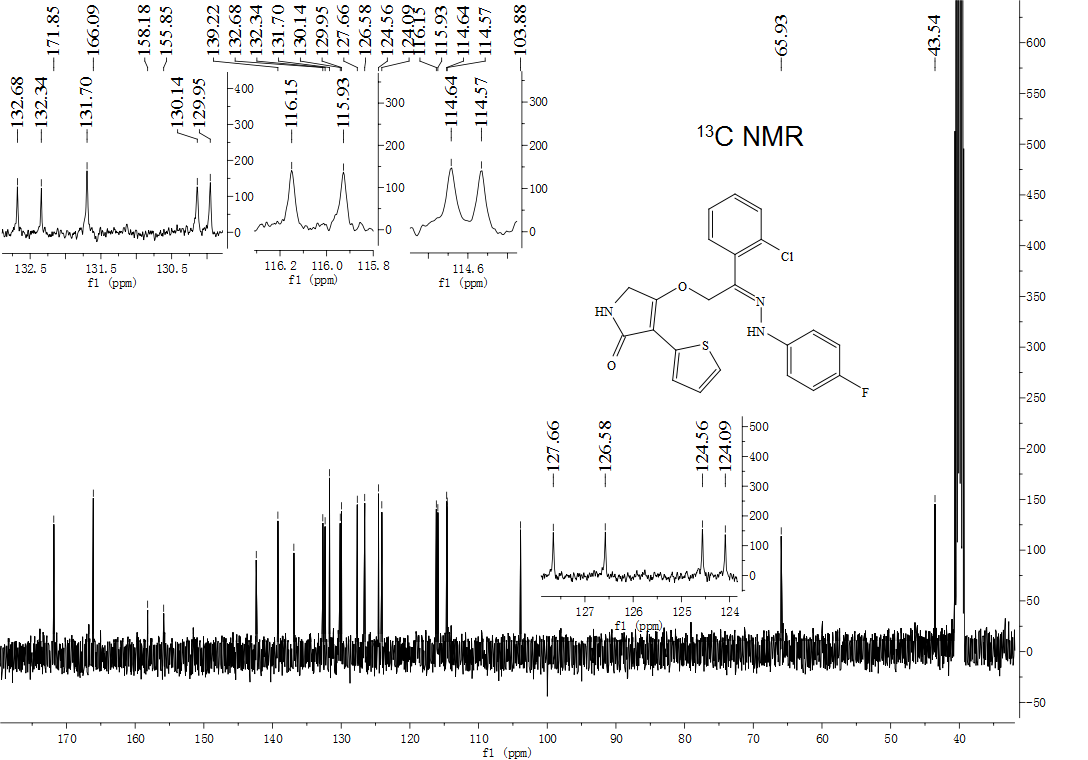
**

Fig. S51 ^13^C NMR sprectrum of title compound **5m**

**
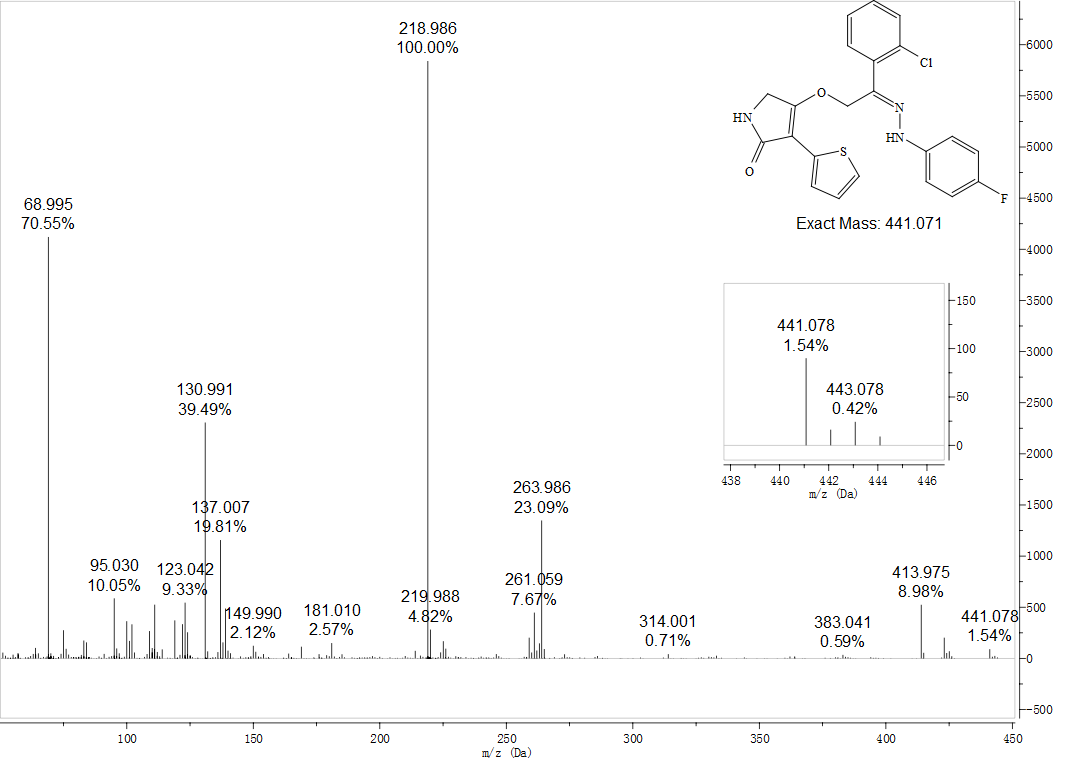
**

Fig. S52 EI-MS sprectrum of title compound **5m**

Fig. S53 IR sprectrum of title compound **5n**

**
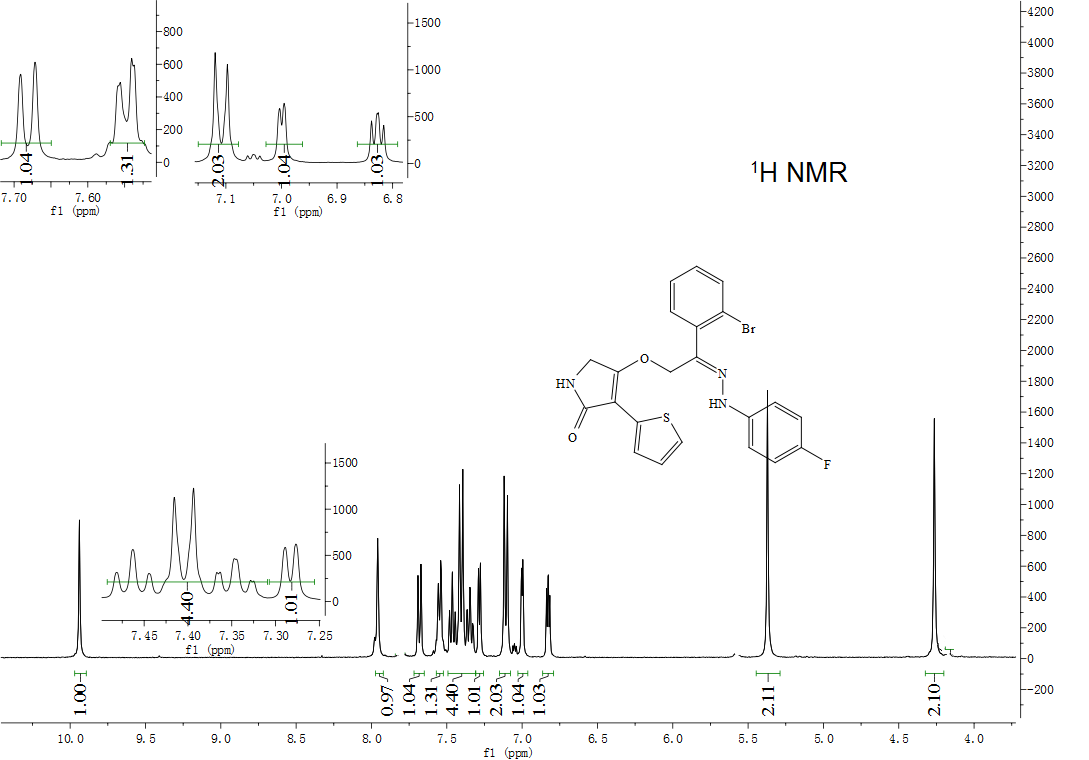
**

Fig. S54 ^1^H NMR sprectrum of title compound **5n**

**
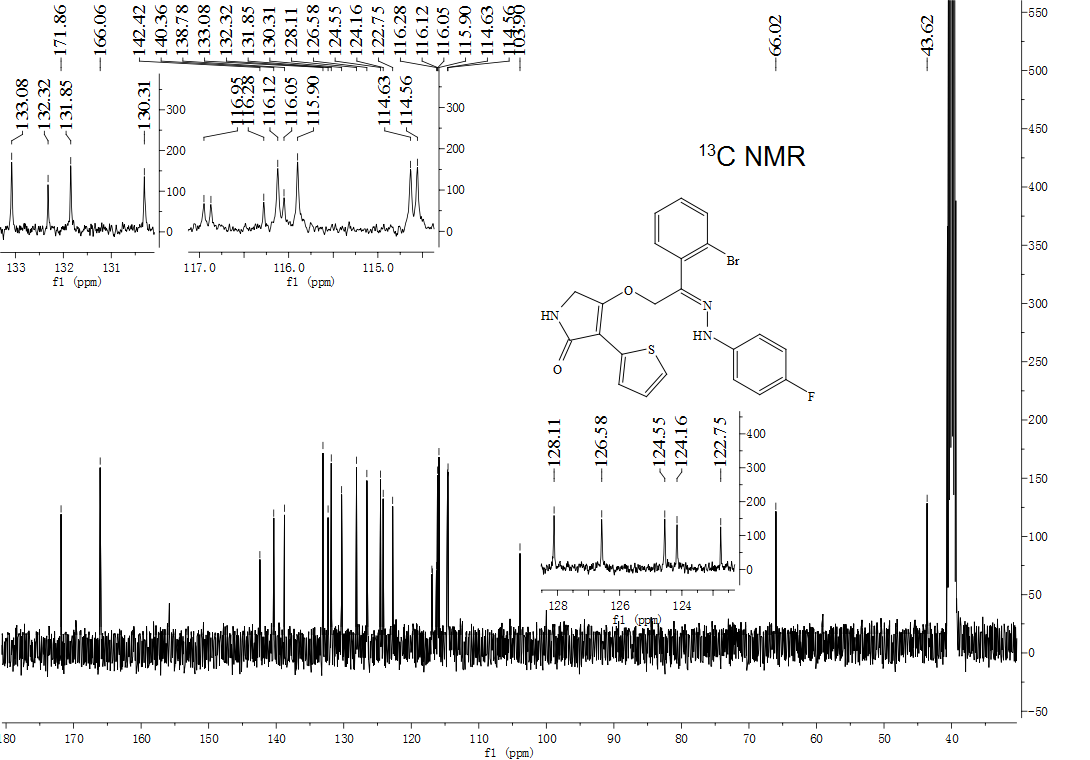
**

Fig. S55 ^13^C NMR sprectrum of title compound **5n**

**
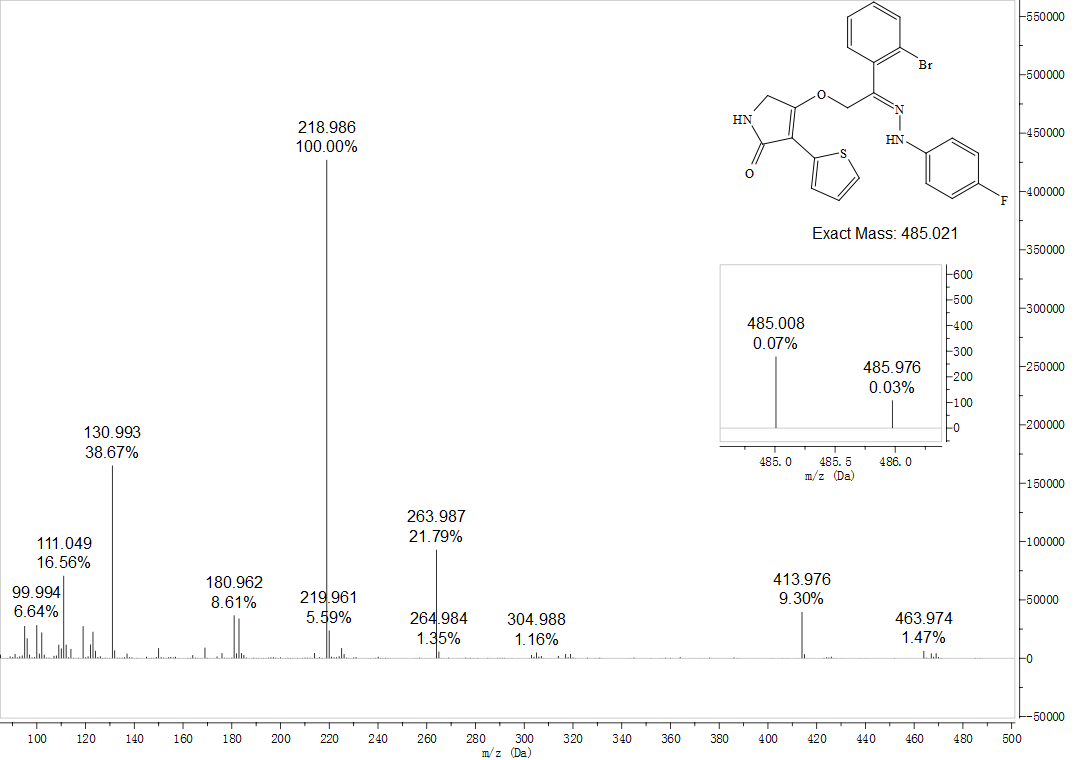
**

Fig. S56 EI-MS sprectrum of title compound **5n**

Fig. S57 IR sprectrum of title compound **5o**

**
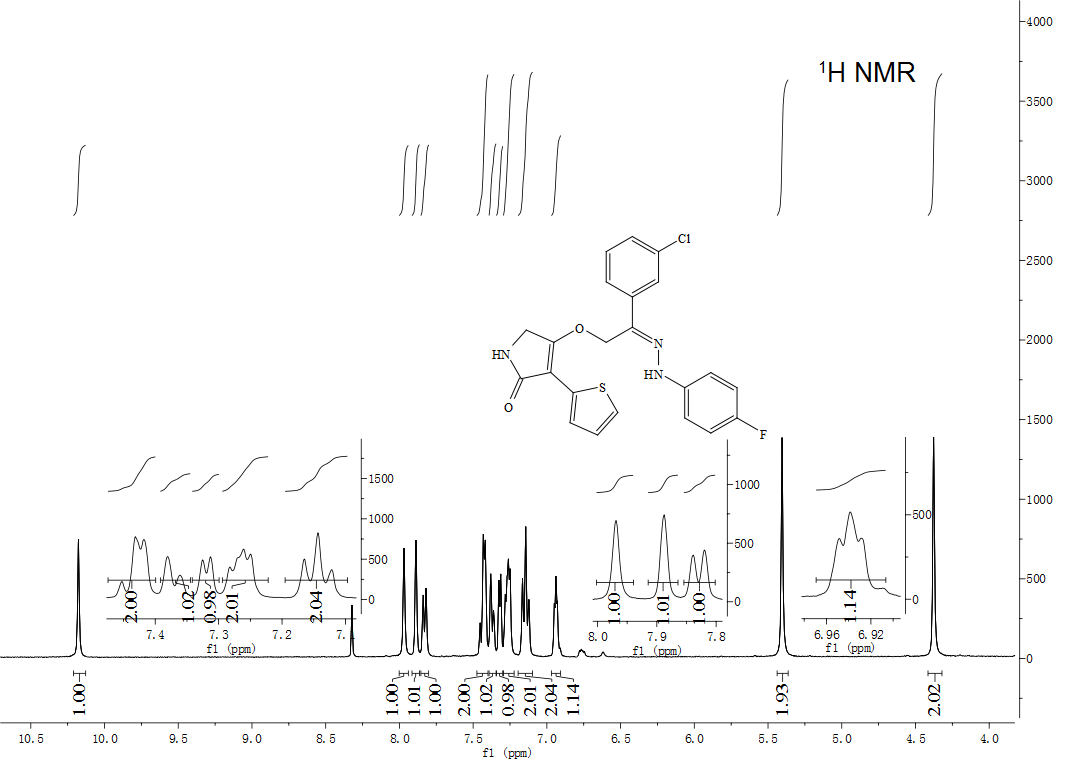
**

Fig. S58 ^1^H NMR sprectrum of title compound **5o**

**
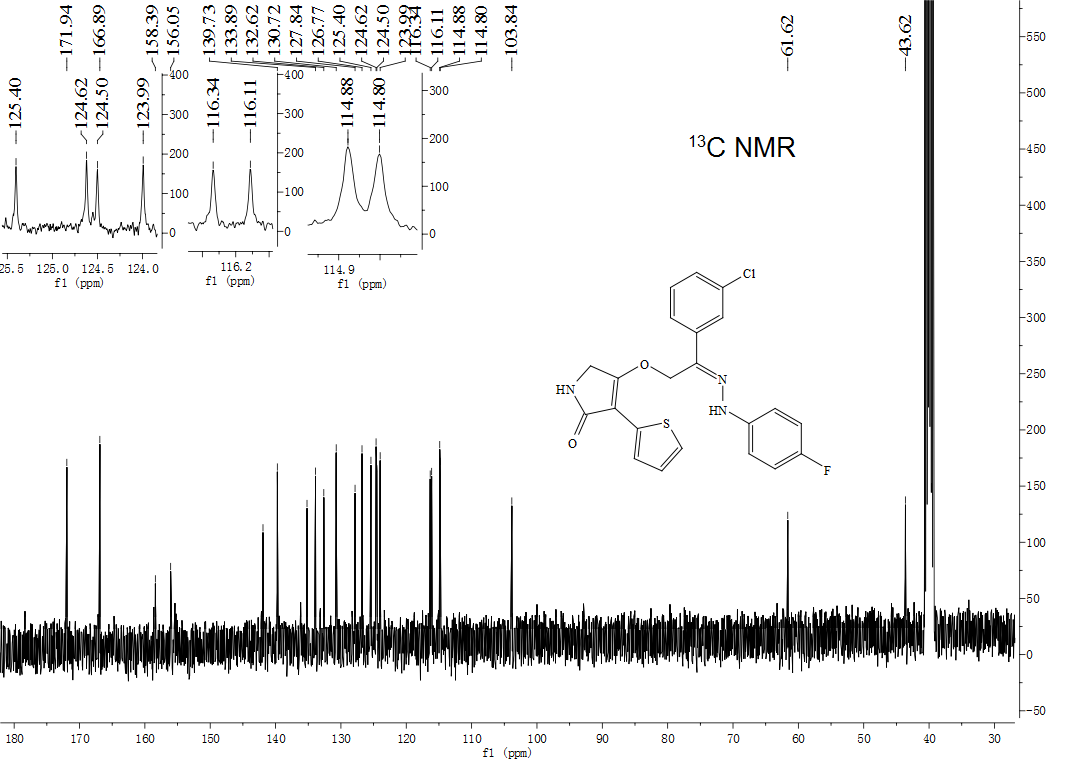
**

Fig. S59 ^13^C NMR sprectrum of title compound **5o**

**
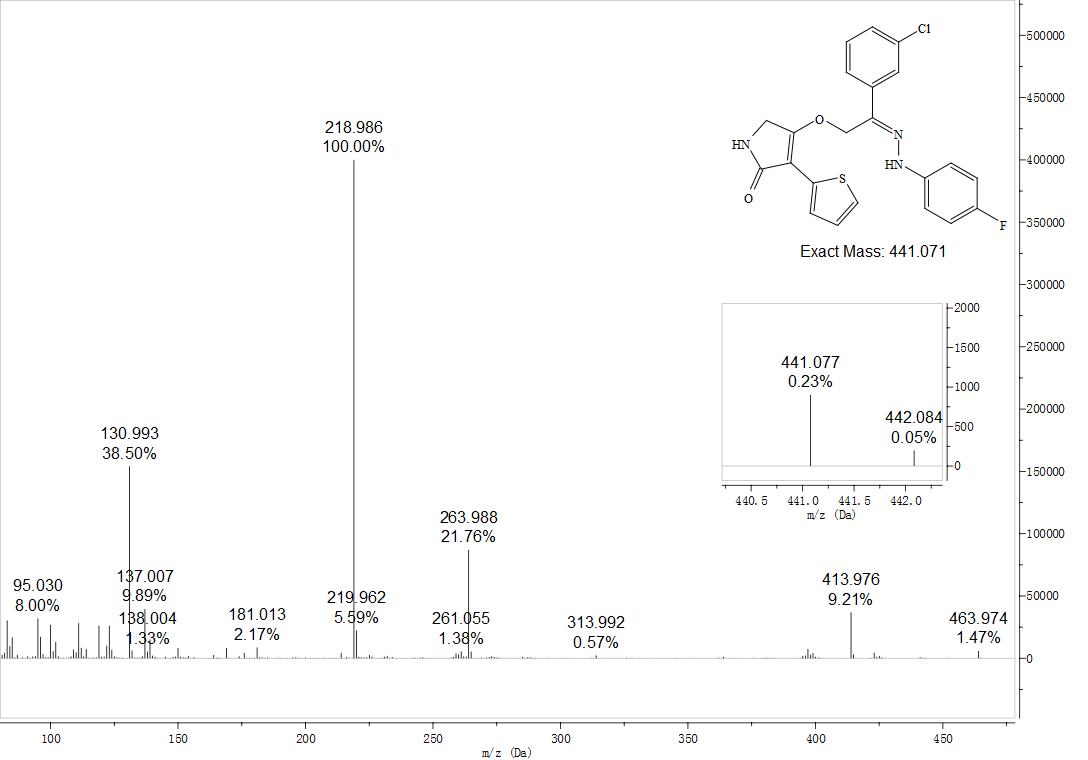
**

Fig. S60 EI-MS sprectrum of title compound **5o**

Fig. S61 IR sprectrum of title compound **5p**

**
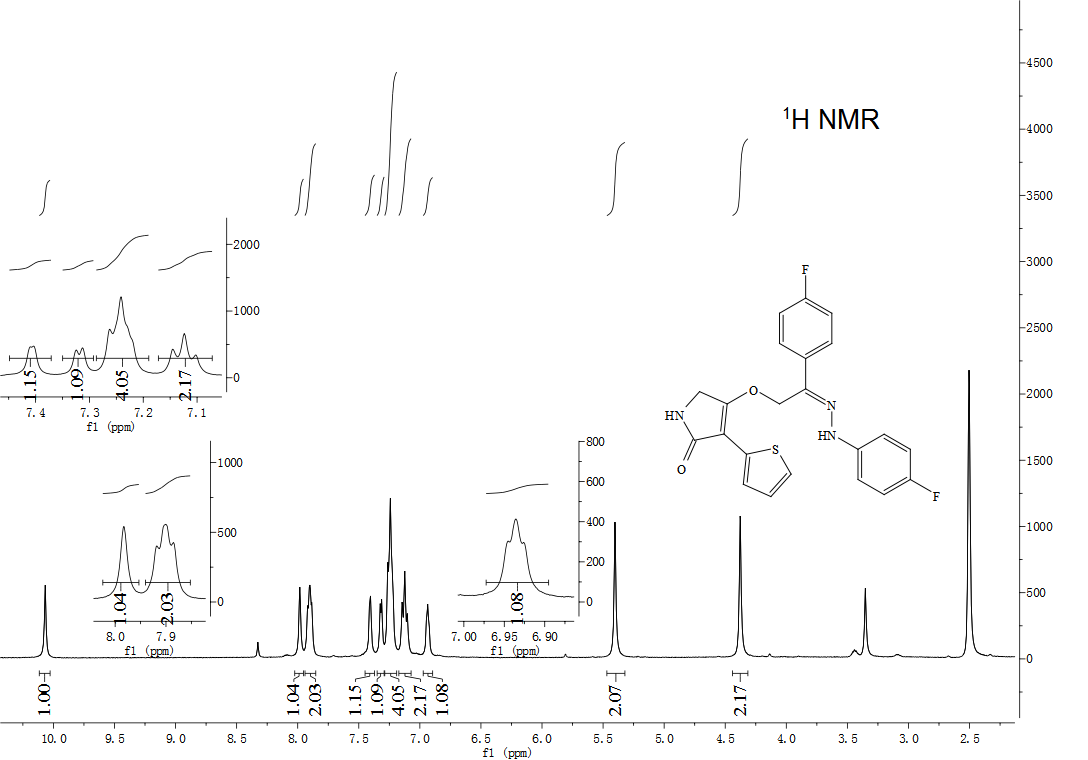
**

Fig. S62 ^1^H NMR sprectrum of title compound **5p**

**
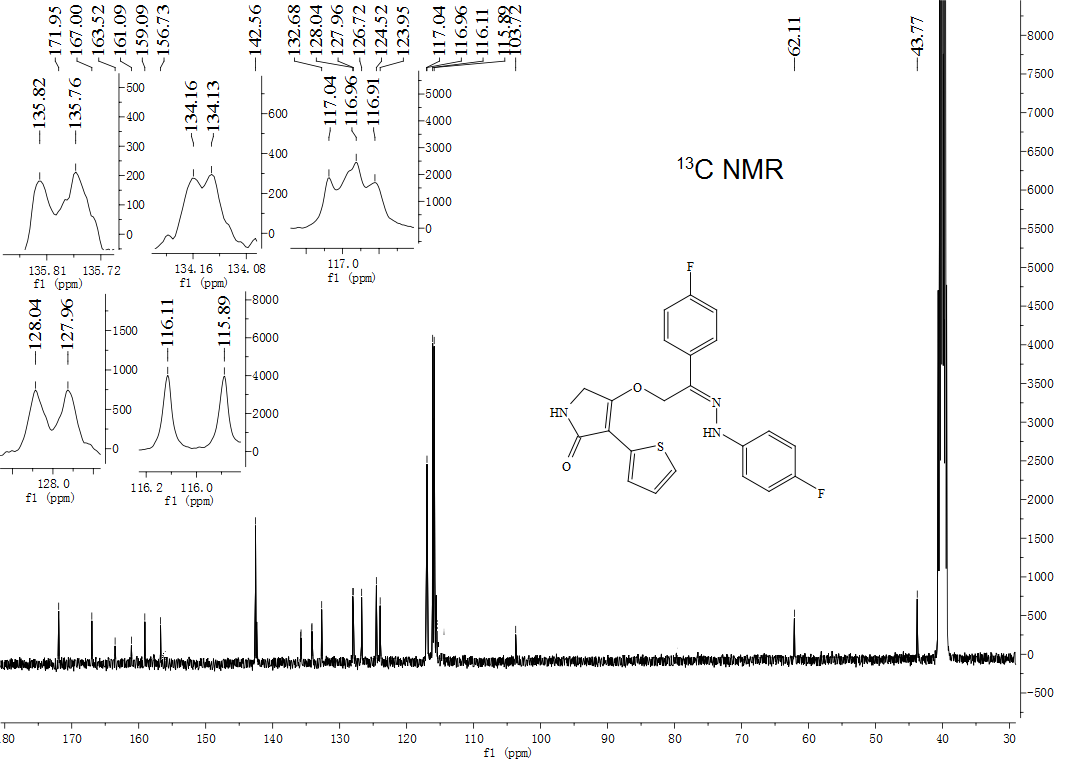
**

Fig. S63 ^13^C NMR sprectrum of title compound **5p**

**
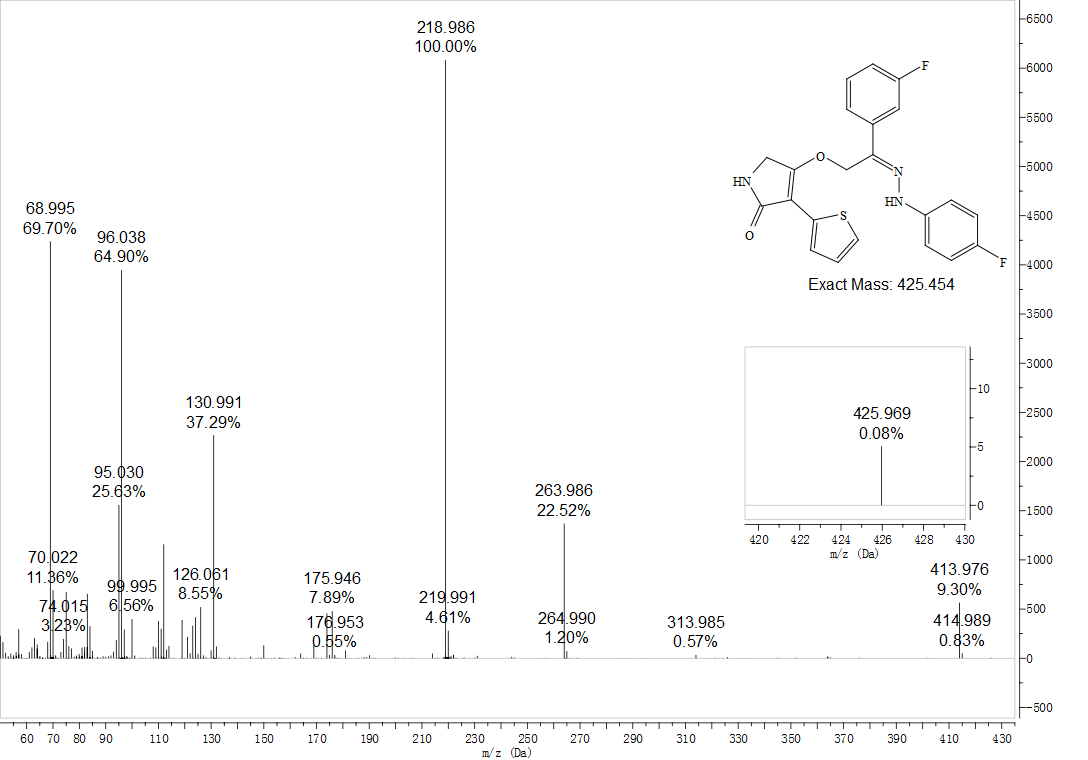
**

Fig. S64 EI-MS sprectrum of title compound **5p**

Fig. S65 IR sprectrum of title compound **5q**

**
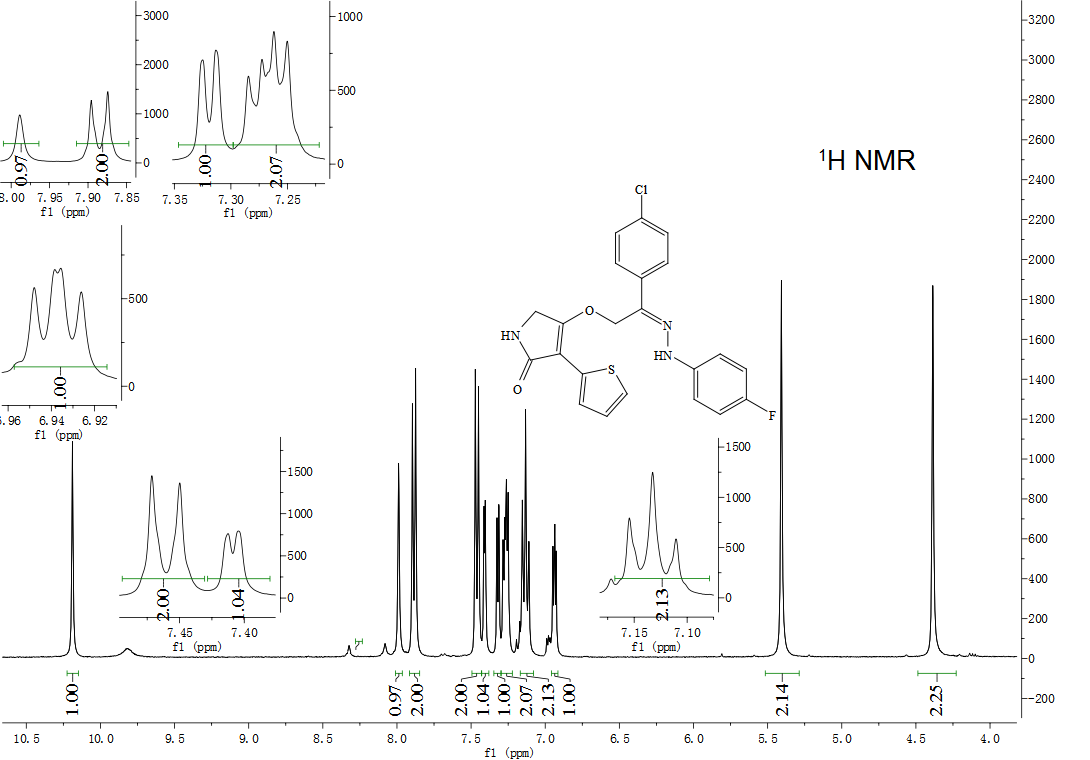
**

Fig. S66 ^1^H NMR sprectrum of title compound **5q**

**
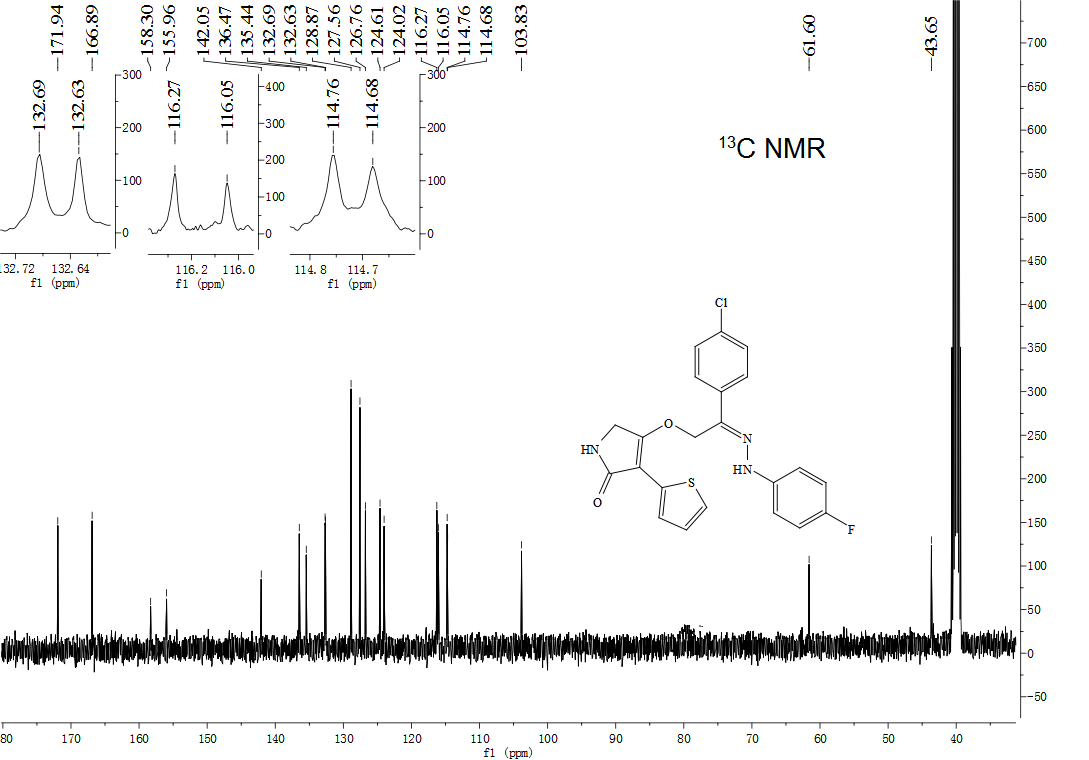
**

Fig. S67 ^13^C NMR sprectrum of title compound **5q**

**
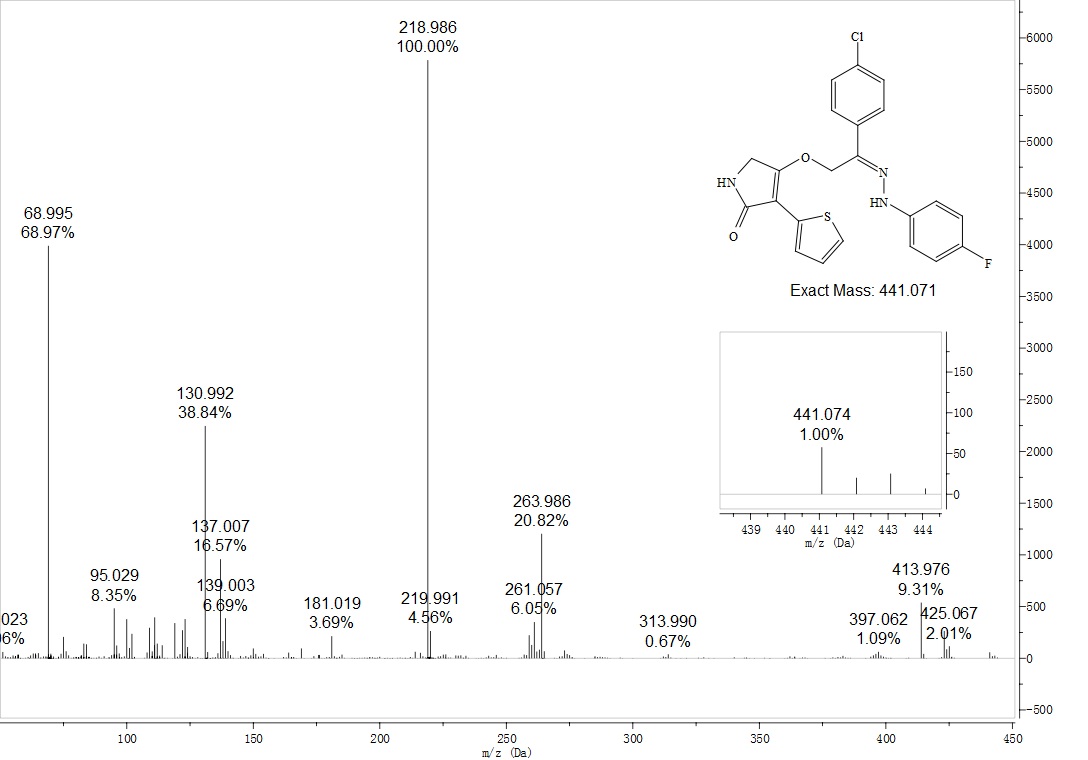
**

Fig. S68 EI-MS sprectrum of title compound **5q**

Fig. S69 IR sprectrum of title compound **5r**

**
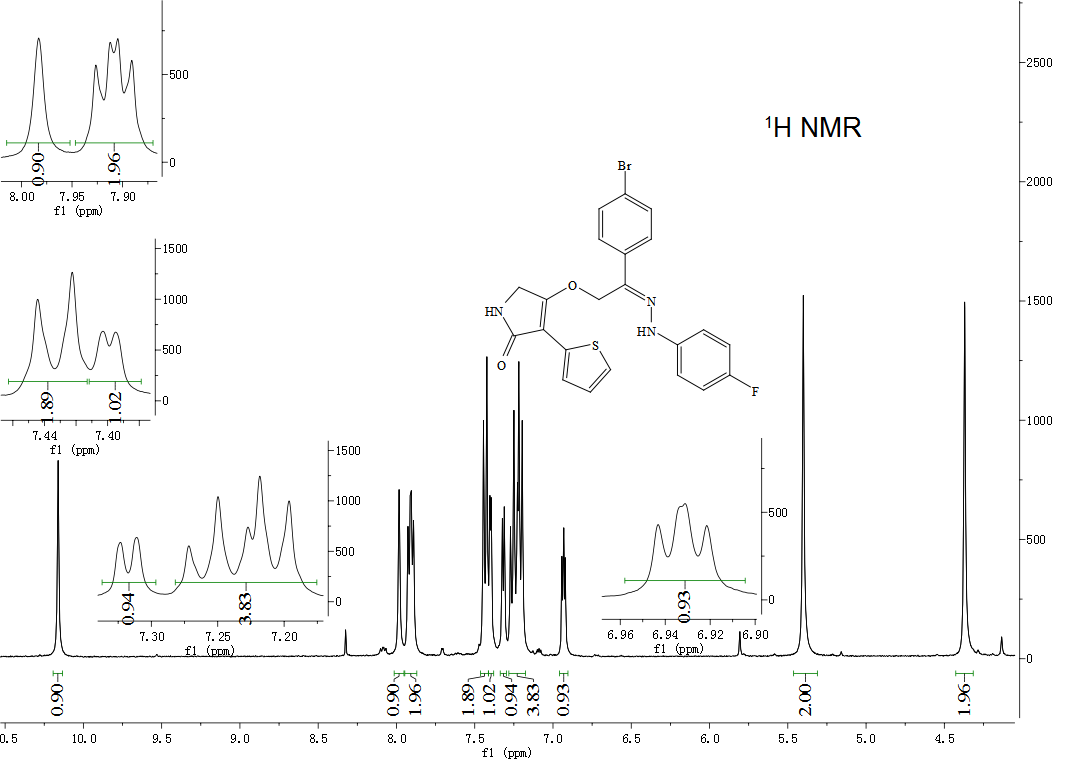
**

Fig. S70 ^1^H NMR sprectrum of title compound **5r**

**
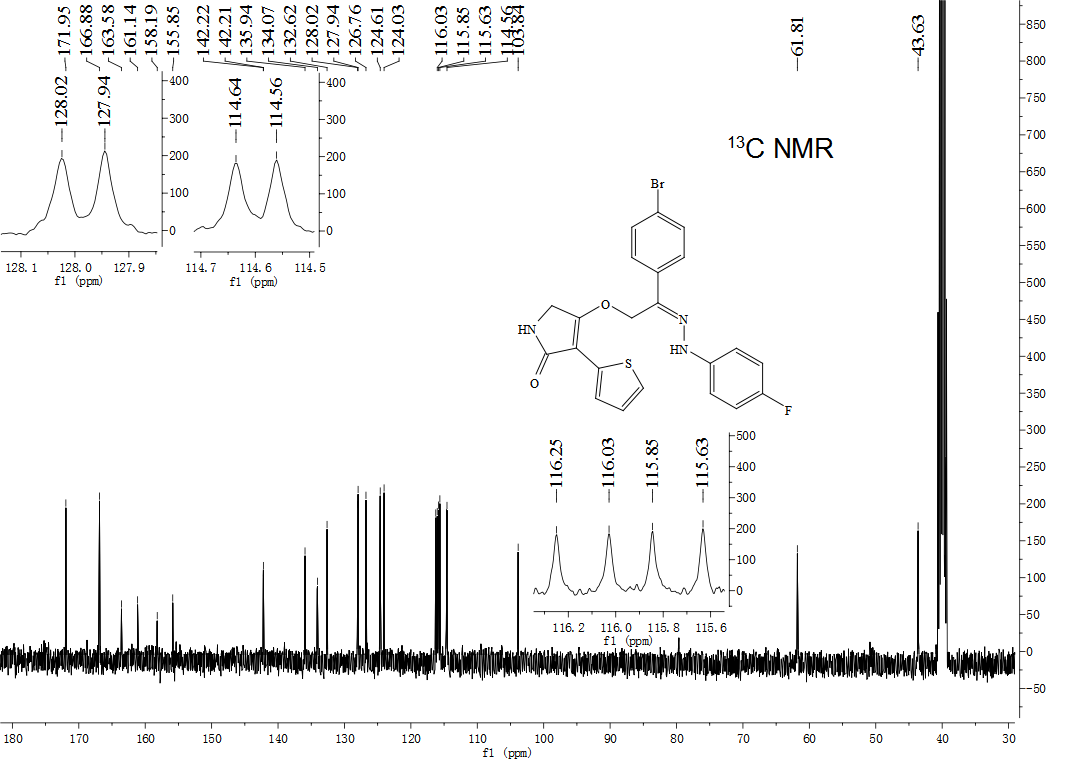
**

Fig. S71 ^13^C NMR sprectrum of title compound **5r**

**
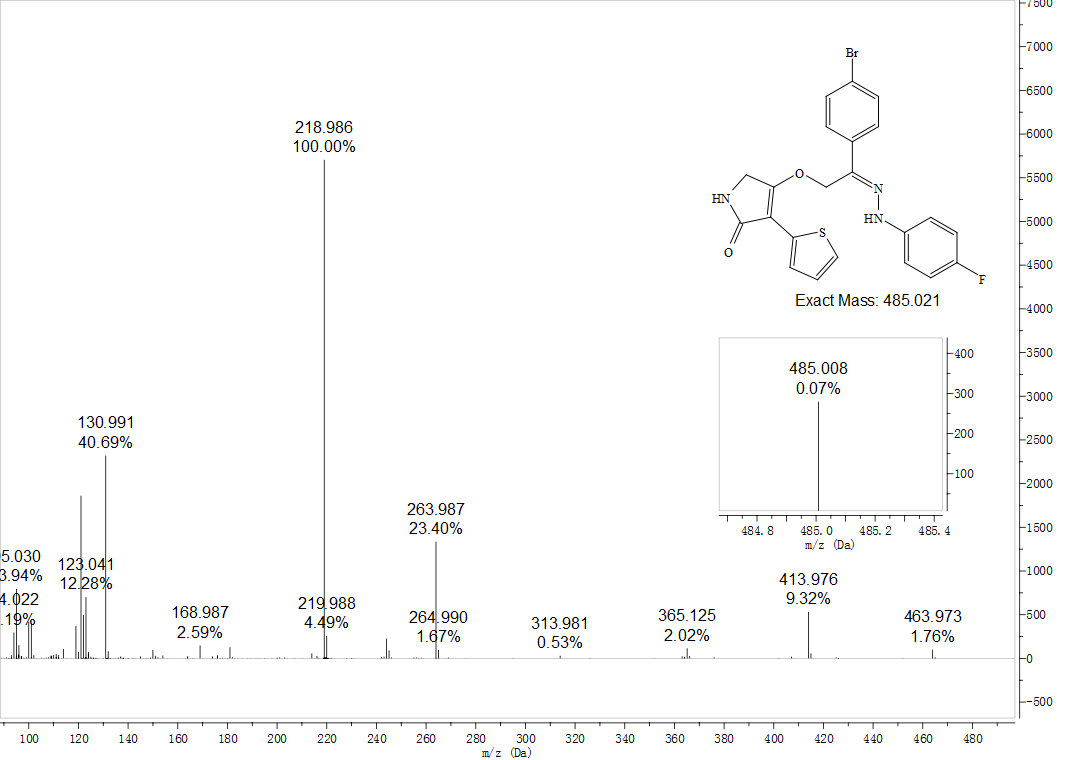
**

Fig. S72 EI-MS sprectrum of title compound **5r**

Fig. S73 IR sprectrum of title compound **5s**

**
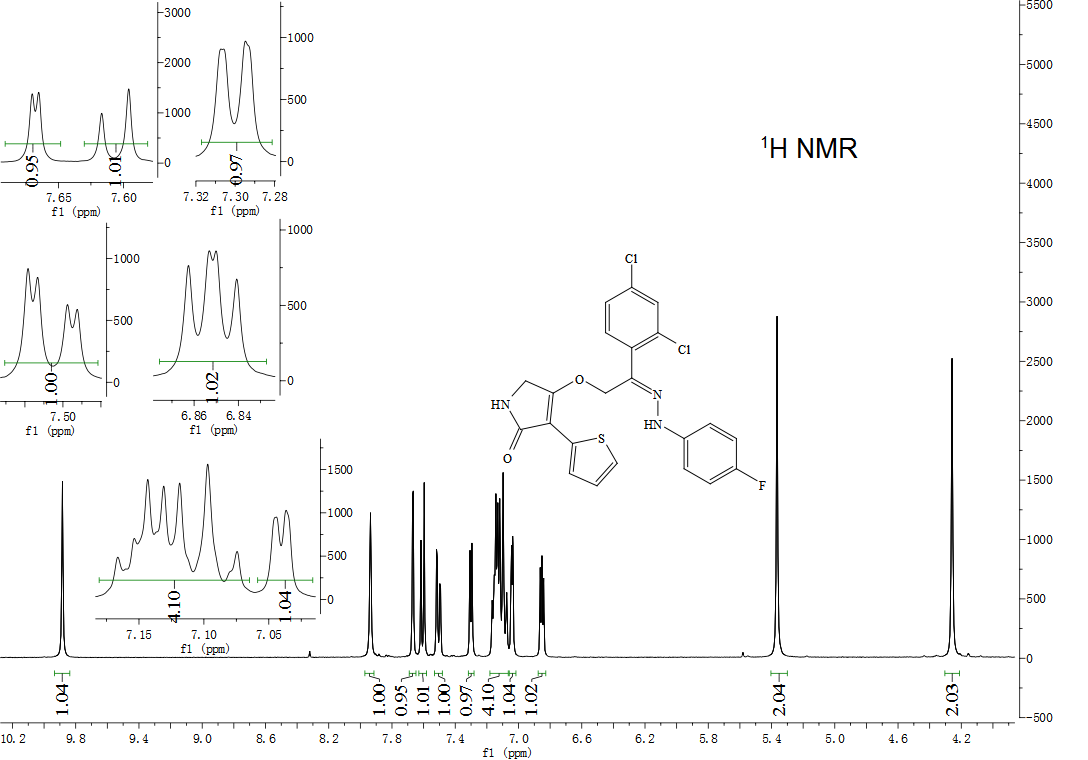
**

Fig. S74 ^1^H NMR sprectrum of title compound **5s**

**
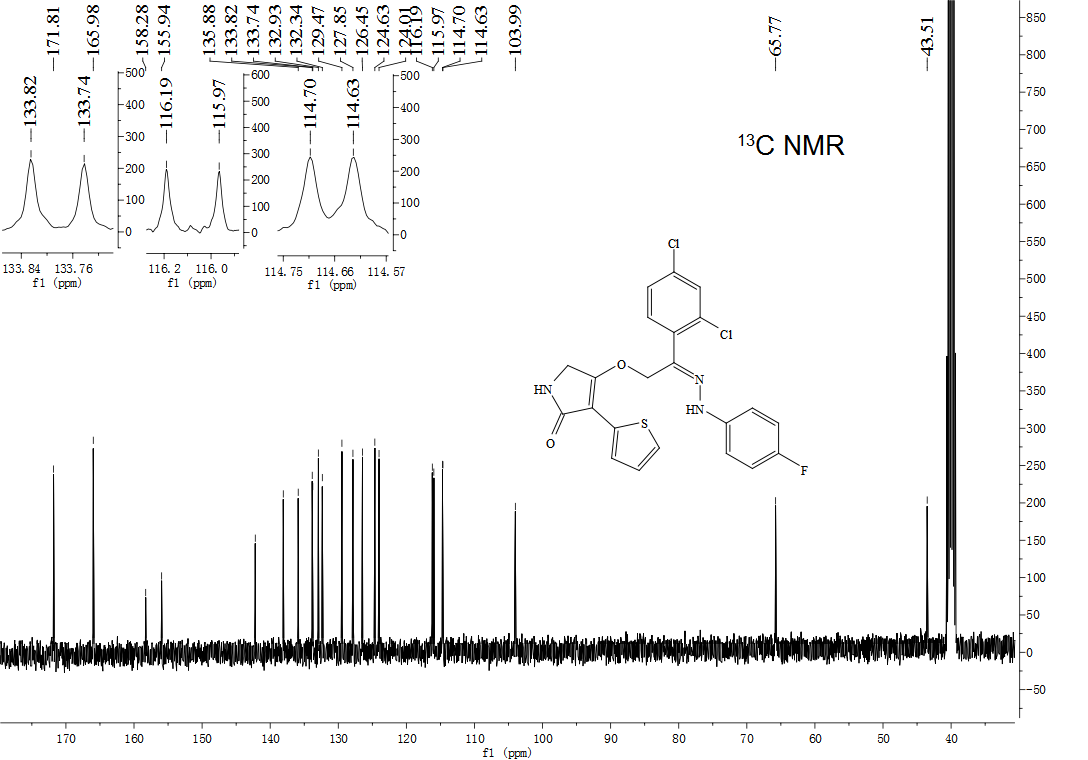
**

Fig. S75 ^13^C NMR sprectrum of title compound **5s**

**
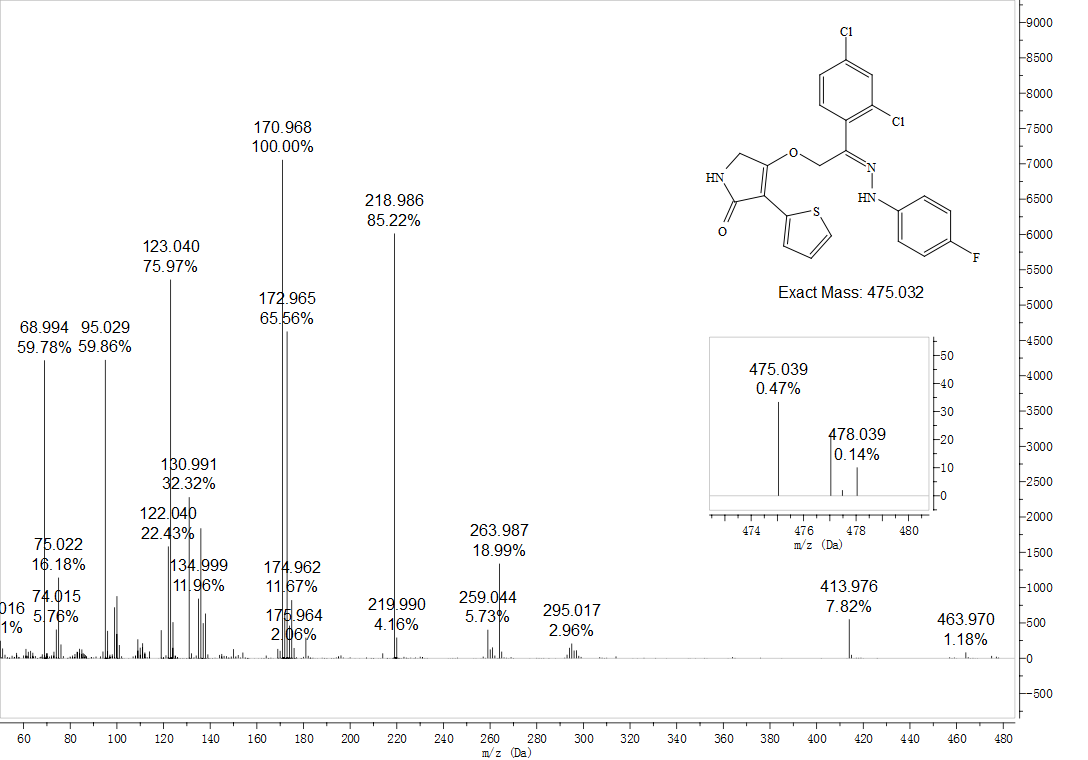
**

Fig. S76 EI-MS sprectrum of title compound **5s**

Fig. S77 IR sprectrum of title compound **5t**

**
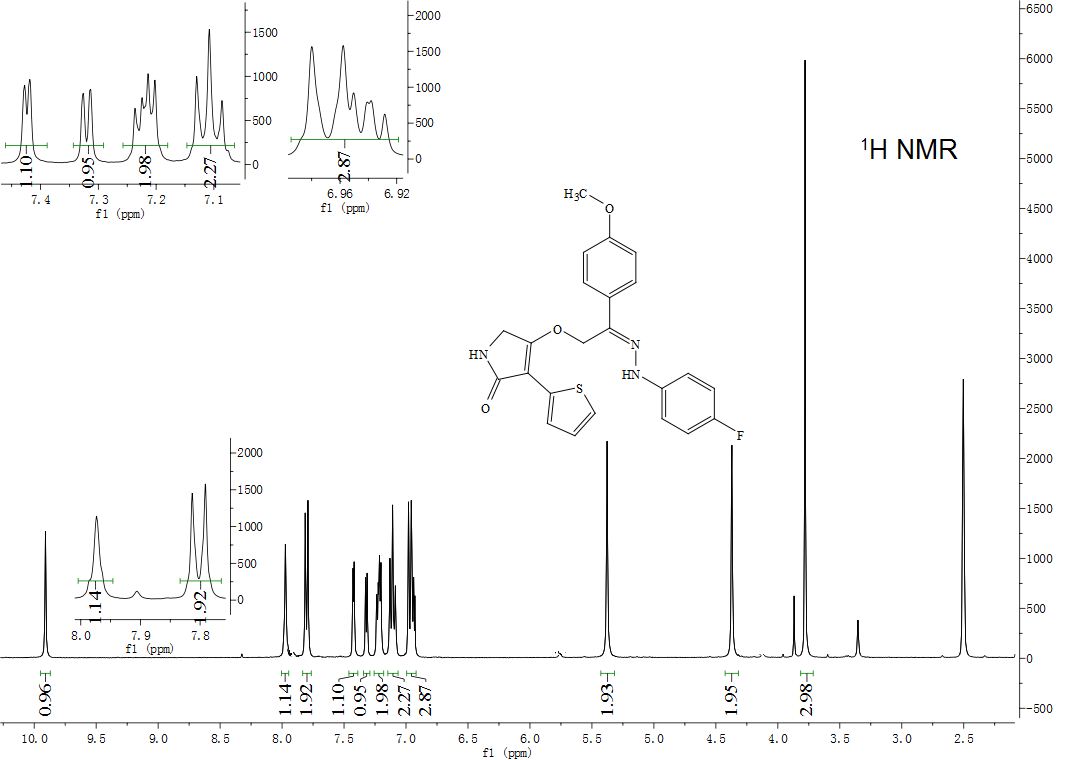
**

Fig. S78 ^1^H NMR sprectrum of title compound **5t**

**
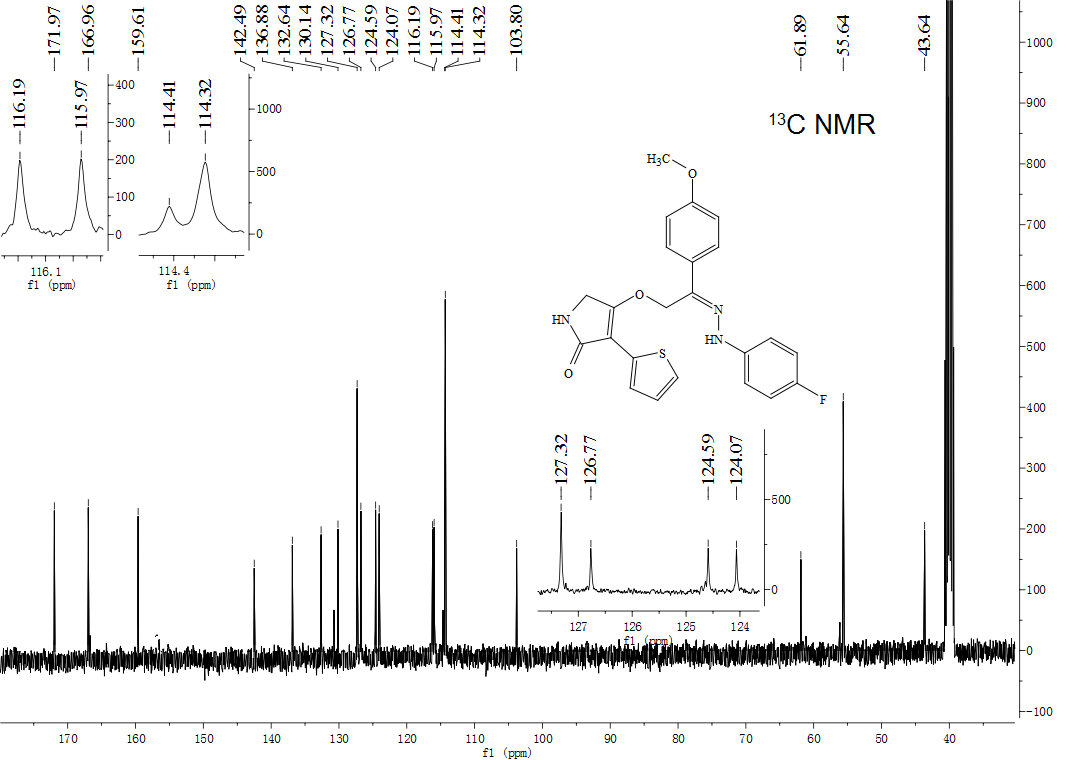
**

Fig. S79 ^13^C NMR sprectrum of title compound **5t**

**
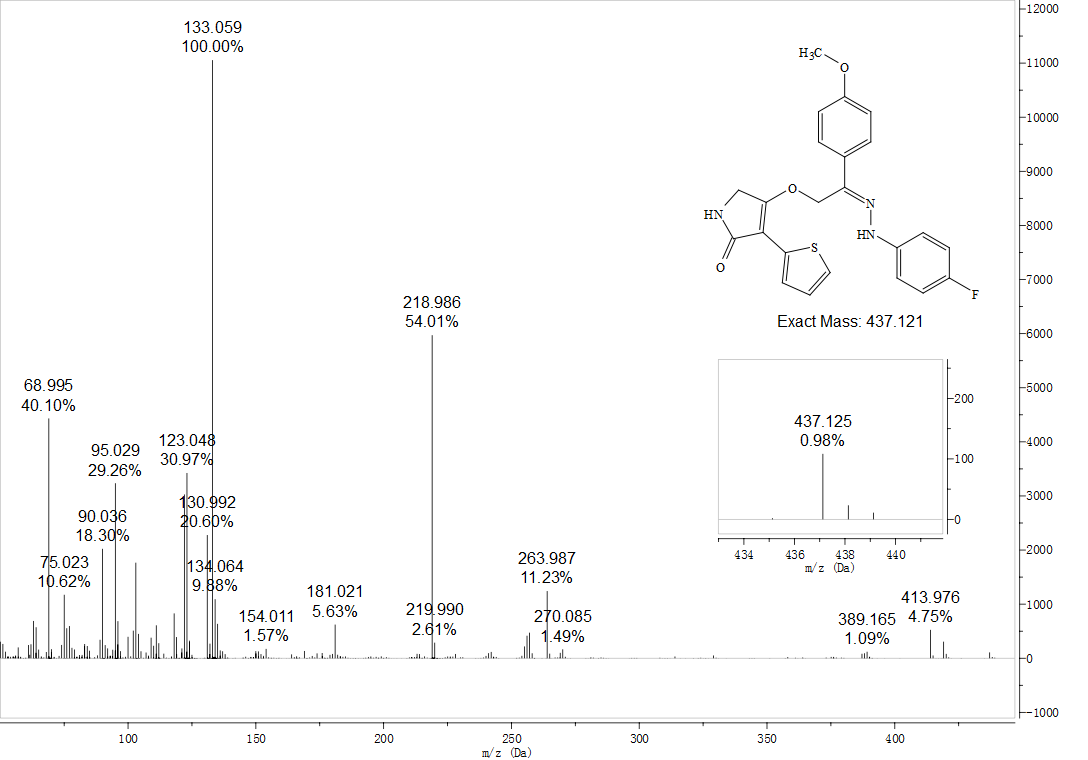
**

Fig. S80 EI-MS sprectrum of title compound **5t**

Fig. S81 IR sprectrum of title compound **5u**

**
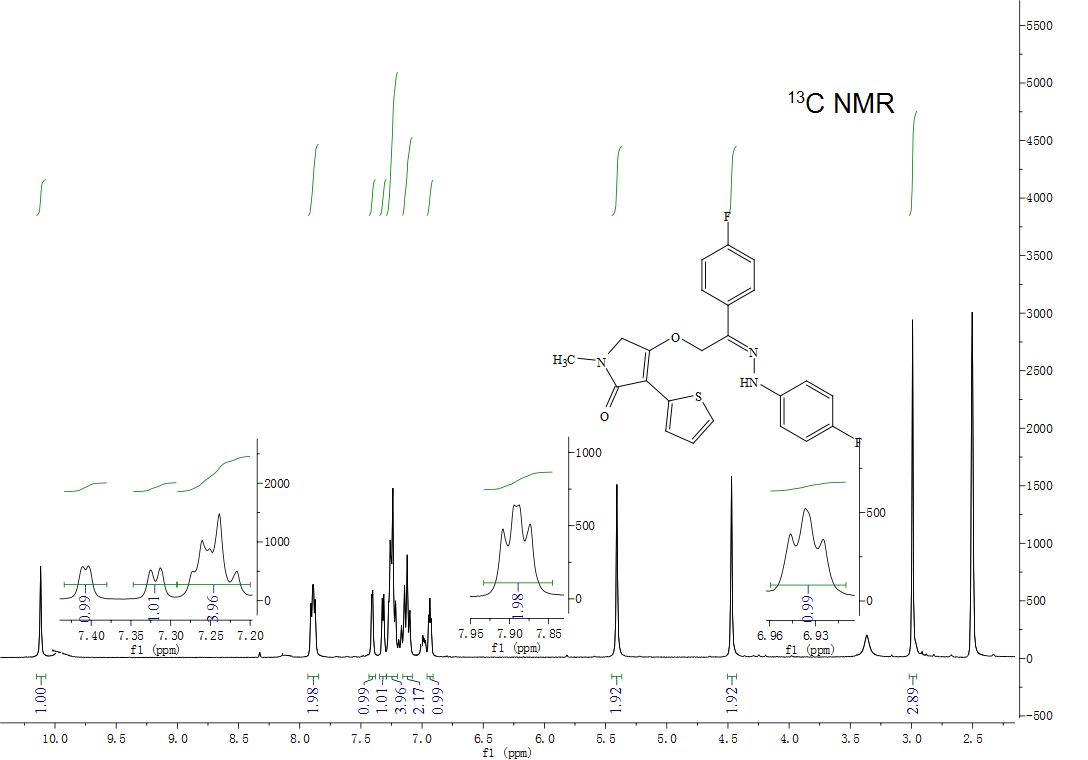
**

Fig. S82 ^1^H NMR sprectrum of title compound **5u**

**
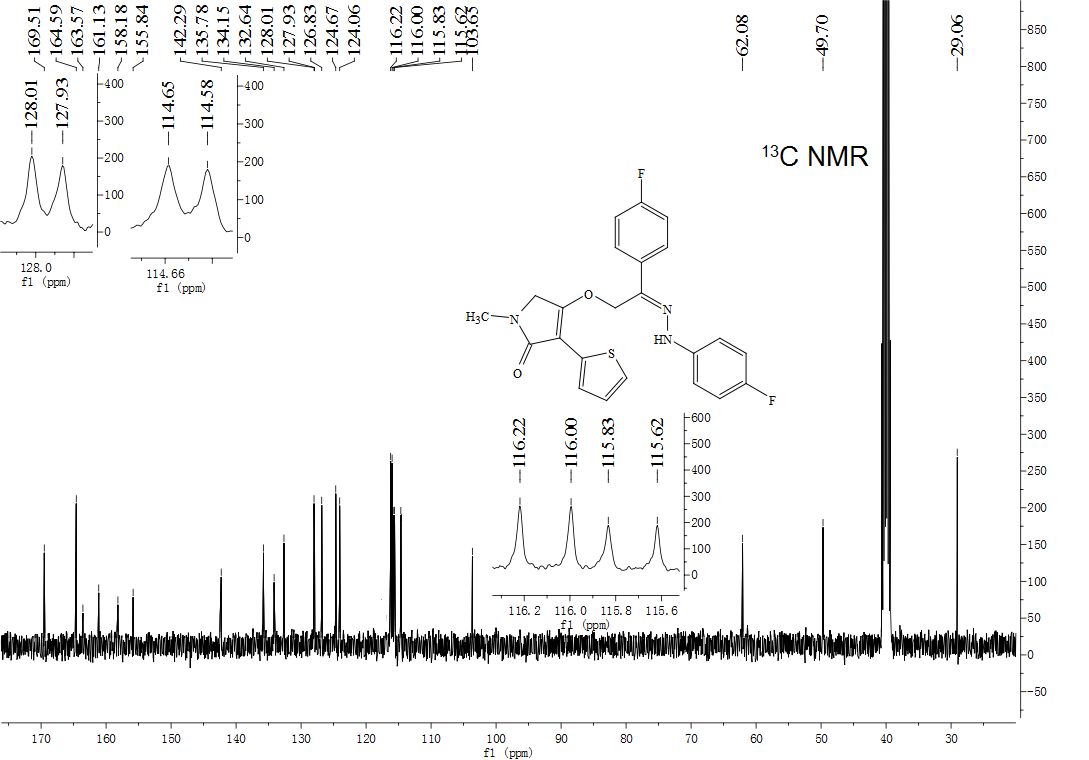
**

Fig. S83 ^13^C NMR sprectrum of title compound **5u**

**
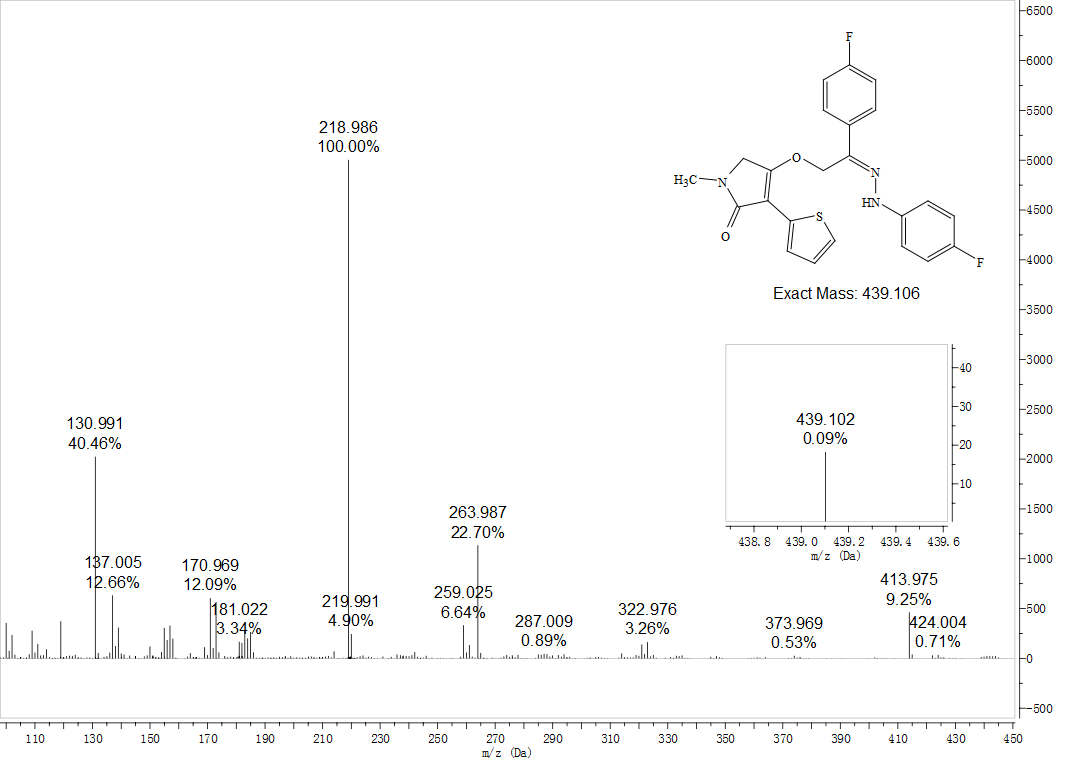
**

Fig. S84 EI-MS sprectrum of title compound **5u**

Fig. S85 IR sprectrum of title compound **5v**

**
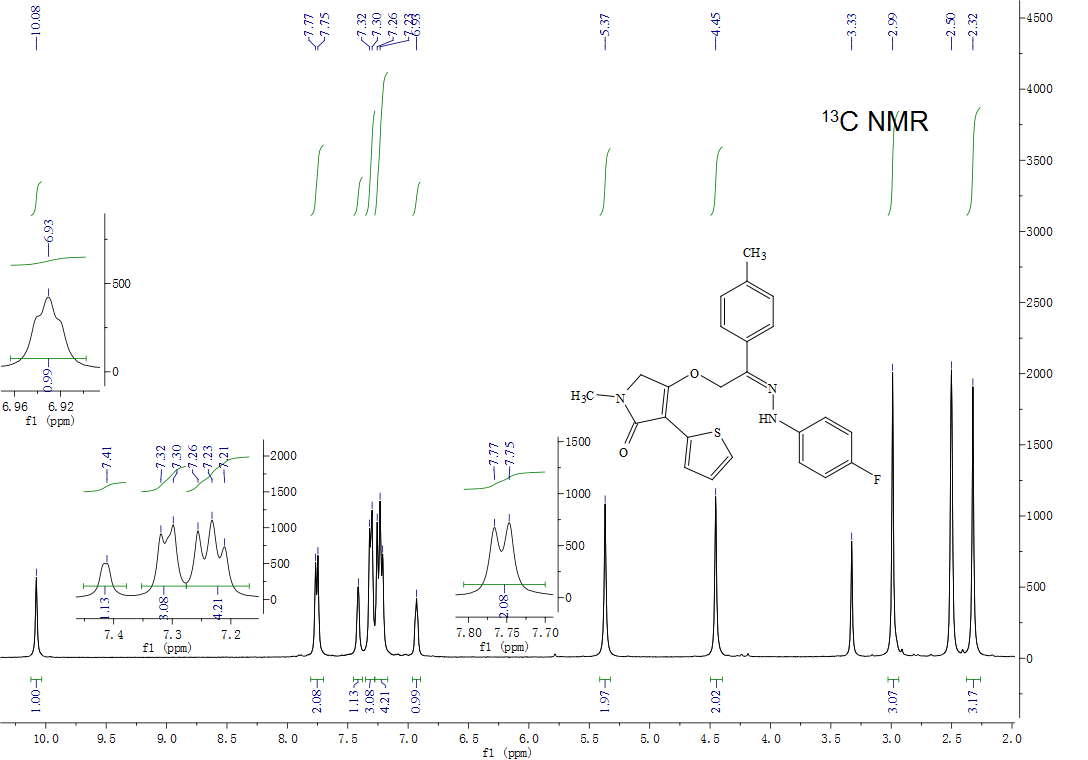
**

Fig. S86 ^1^H NMR sprectrum of title compound **5v**

**
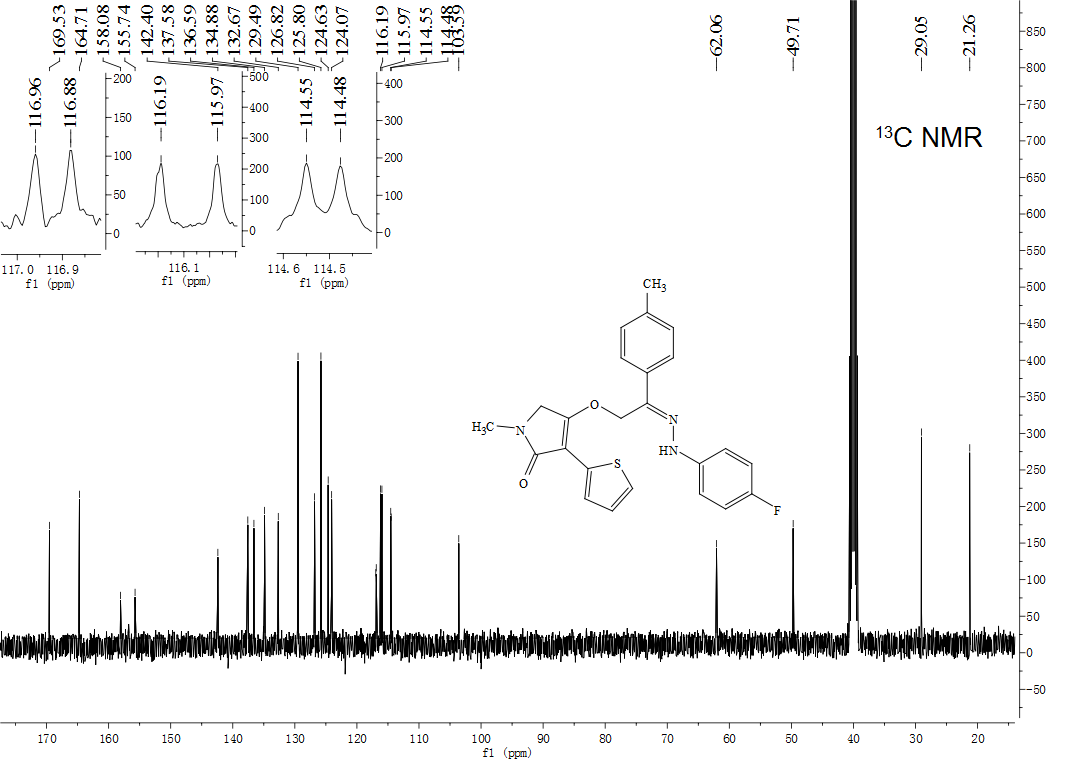
**

Fig. S87 ^13^C NMR sprectrum of title compound **5v**

**
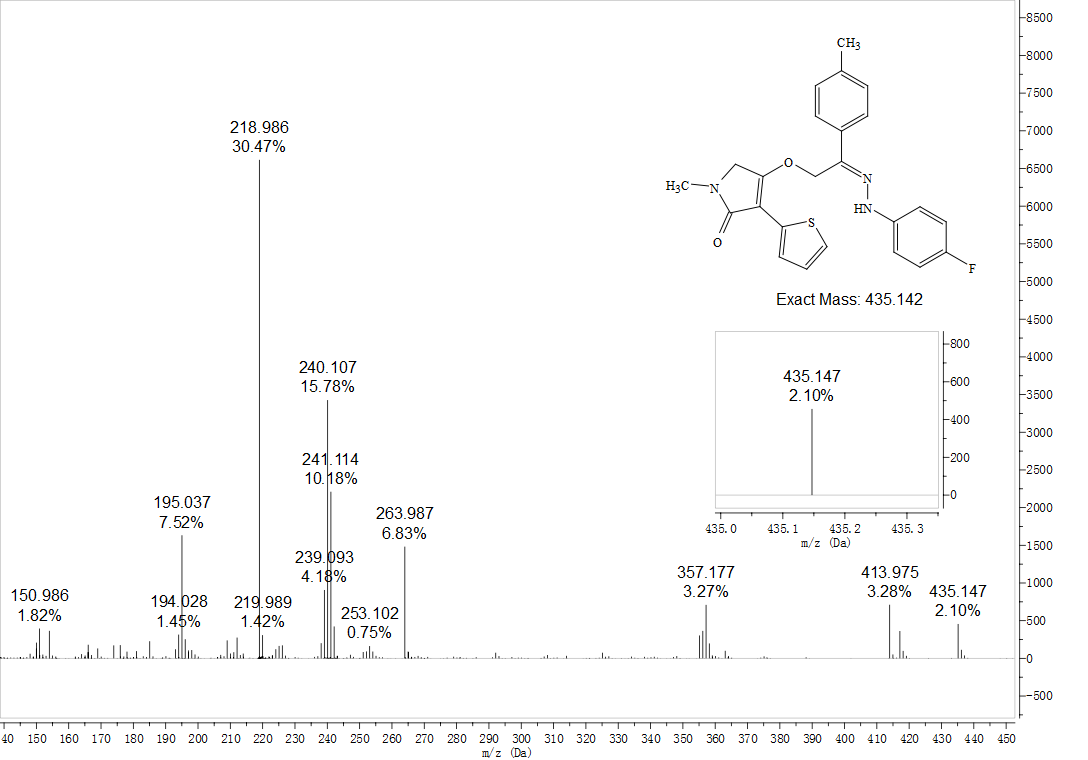
**

Fig. S88 EI-MS sprectrum of title compound **5v**

Fig. S89 IR sprectrum of title compound **5w**

**
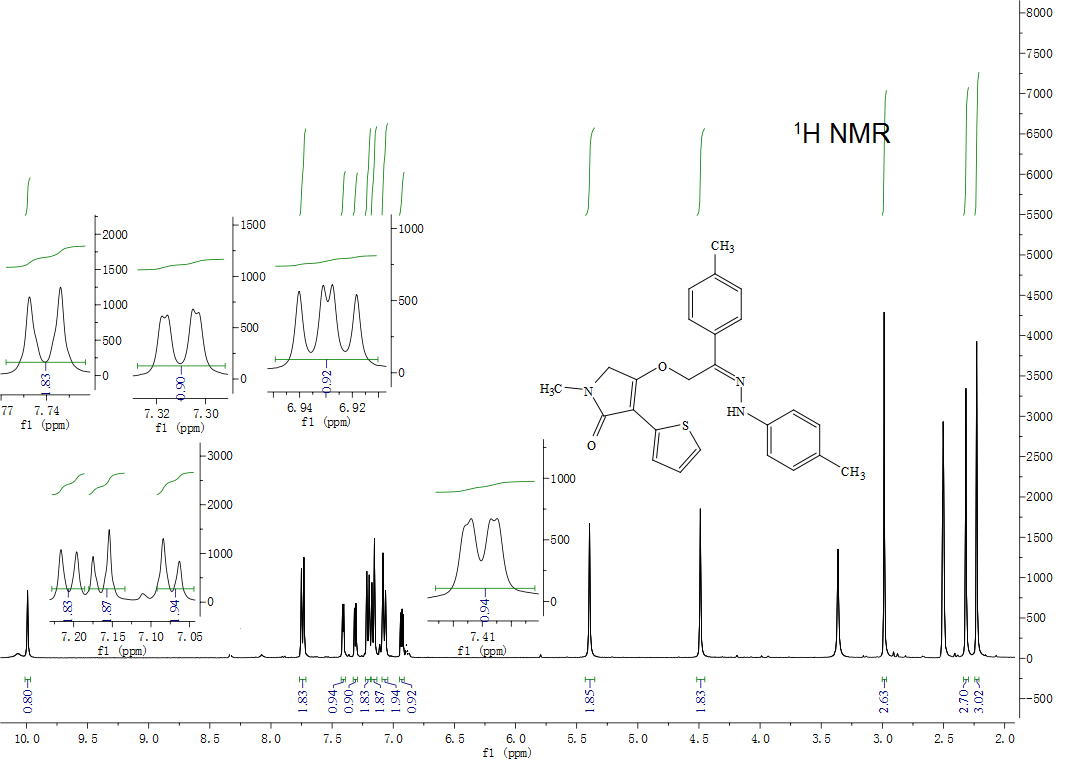
**

Fig. S90 ^1^H NMR sprectrum of title compound **5w**

**
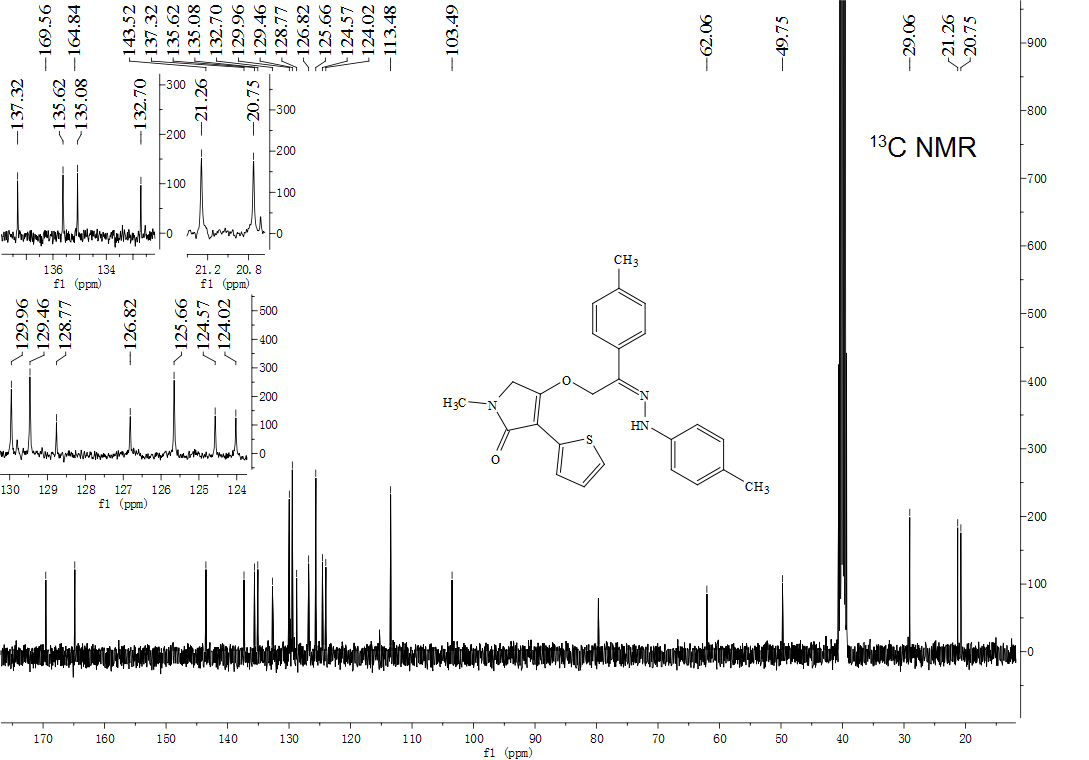
**

Fig. S91 ^13^C NMR sprectrum of title compound **5w**


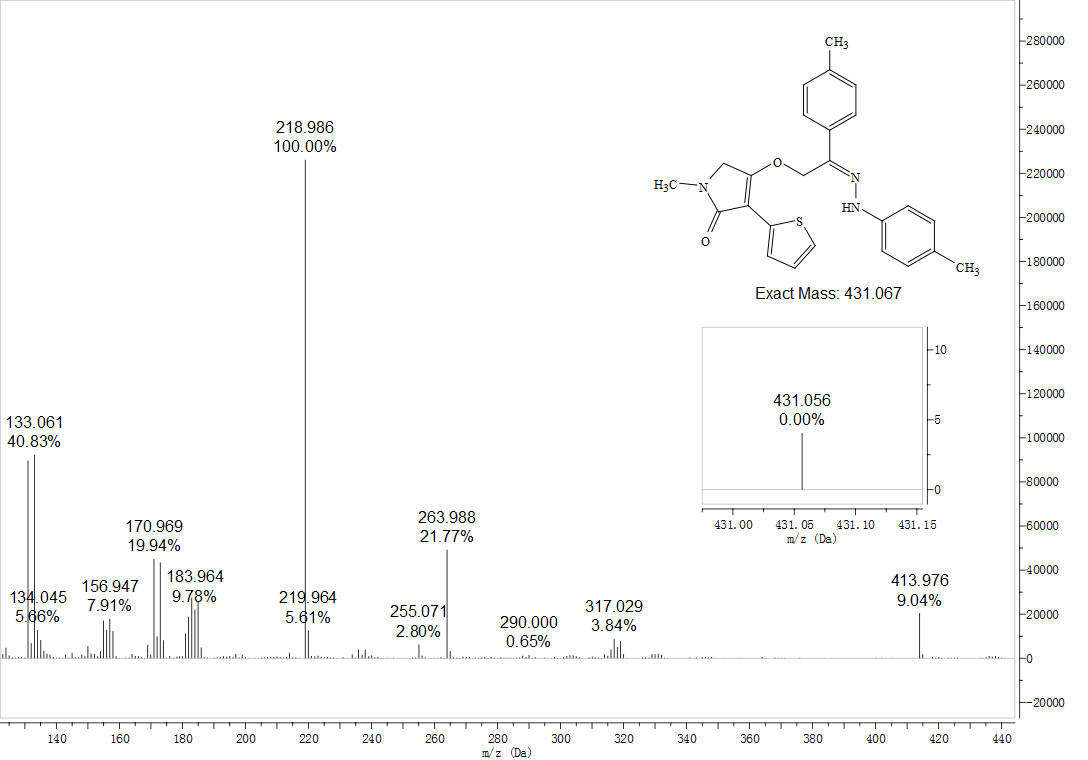


Fig. S92 EI-MS sprectrum of title compound **5w**
